# Prognostic value analysis of cholesterol and cholesterol homeostasis related genes in breast cancer by Mendelian randomization and multi-omics machine learning

**DOI:** 10.3389/fonc.2023.1246880

**Published:** 2023-11-07

**Authors:** Haodong Wu, Zhixuan Wu, Daijiao Ye, Hongfeng Li, Yinwei Dai, Ziqiong Wang, Jingxia Bao, Yiying Xu, Xiaofei He, Xiaowu Wang, Xuanxuan Dai

**Affiliations:** ^1^ Department of Breast Surgery, The First Affiliated Hospital of Wenzhou Medical University, Wenzhou, China; ^2^ Department of Burns and Skin Repair Surgery, The Third Affiliated Hospital of Wenzhou Medical University, Ruian, Zhejiang, China; ^3^ Key Laboratory of Clinical Laboratory Diagnostics (Ministry of Education), The First Affiliated Hospital of Wenzhou Medical University, Wenzhou, China; ^4^ Medical Research Center, The First Affiliated Hospital of Wenzhou Medical University, Wenzhou, China

**Keywords:** Mendelian randomization, breast cancer, immune microenvironment, cholesterol homeostasis, prognosis prediction, machine learning method

## Abstract

**Introduction:**

The high incidence of breast cancer (BC) prompted us to explore more factors that might affect its occurrence, development, treatment, and also recurrence. Dysregulation of cholesterol metabolism has been widely observed in BC; however, the detailed role of how cholesterol metabolism affects chemo-sensitivity, and immune response, as well as the clinical outcome of BC is unknown.

**Methods:**

With Mendelian randomization (MR) analysis, the potential causal relationship between genetic variants of cholesterol and BC risk was assessed first. Then we analyzed 73 cholesterol homeostasis-related genes (CHGs) in BC samples and their expression patterns in the TCGA cohort with consensus clustering analysis, aiming to figure out the relationship between cholesterol homeostasis and BC prognosis. Based on the CHG analysis, we established a CAG_score used for predicting therapeutic response and overall survival (OS) of BC patients. Furthermore, a machine learning method was adopted to accurately predict the prognosis of BC patients by comparing multi-omics differences of different risk groups.

**Results:**

We observed that the alterations in plasma cholesterol appear to be correlative with the venture of BC (MR Egger, OR: 0.54, 95% CI: 0.35-0.84, p<0.006). The expression patterns of CHGs were classified into two distinct groups(C1 and C2). Notably, the C1 group exhibited a favorable prognosis characterized by a suppressed immune response and enhanced cholesterol metabolism in comparison to the C2 group. In addition, high CHG score were accompanied by high performance of tumor angiogenesis genes. Interestingly, the expression of vascular genes (CDH5, CLDN5, TIE1, JAM2, TEK) is lower in patients with high expression of CHGs, which means that these patients have poorer vascular stability. The CAG_score exhibits robust predictive capability for the immune microenvironment characteristics and prognosis of patients(AUC=0.79). It can also optimize the administration of various first-line drugs, including AKT inhibitors VIII Imatinib, Crizotinib, Saracatinib, Erlotinib, Dasatinib, Rapamycin, Roscovitine and Shikonin in BC patients. Finally, we employed machine learning techniques to construct a multi-omics prediction model(Risklight),with an area under the feature curve (AUC) of up to 0.89.

**Conclusion:**

With the help of CAG_score and Risklight, we reveal the signature of cholesterol homeostasis-related genes for angiogenesis, immune responses, and the therapeutic response in breast cancer, which contributes to precision medicine and improved prognosis of BC.

## 1 Introduction

According to the World Health Organization (WHO) report in 2021, breast cancer (BC) has become the most prevalent tumor in the world with the increasing incidence (1). Attribute to the progress of surgical treatment and the application of immunotherapy, its survival rate is also higher than other tumors, but there is still a high recurrence rate, and the recurrence rate of patients who receive postoperative radiotherapy can reach 15% within 10 years (2). Therefore, it is particularly important to explore techniques and biomarkers for early identification and prevention of recurrence.

In addition to the effects at the genetic level, some studies have pointed out that the disruption of cellular cholesterol levels’ dynamic balance can lead to cancer occurrence and a series of diseases (3). Elevated serum cholesterol is associated with the risk of melanoma, prostate cancer, endometrial cancer, non-Hodgkin’s lymphoma, and breast cancer (3–5). Hypercholesterolemia has been identified as a comorbidity of obesity, becoming an independent risk factor for breast cancer in postmenopausal women. Dysregulation of cholesterol homeostasis can also lead to ferroptosis resistance, thereby increasing tumor tumorigenicity and metastatic capacity (6). However, most current studies have focused on determining the role of serum cholesterol or liver cholesterol in the progression and prognosis of BC (7, 8), while neglecting the involvement of cholesterol homeostasis-related genes (CHGs) in tumorigenesis.

Furthermore, the tumor microenvironment (TME) has garnered increasing attention (9). Tumor growth environment is a complex tissue environment, which is closely related to tumor growth, invasion, metastasis, and other functions. Under the induction of tumor cells, stromal cells in TME lead to increased angiogenesis and immune escape of tumor cells. The mechanism of immune cells such as T cells and tumor-associated macrophages (TAMs) involved in this process has attracted many scholars to explore, which means that TME can become a potential therapeutic target (10). At the same time, it has also been found that intracellular cholesterol metabolism has an important impact on the tumor-inhibitory effect of CD8+ T cells (11). However, the precise mechanisms underlying the interaction between TME and cholesterol metabolism as well as tumor immune evasion remain elusive.

Hence, we conducted a comprehensive analysis of the expression of CHGs and its impact on the tumor microenvironment (TME), disease progression, treatment response, and prognosis in breast cancer (BC) patients. Leveraging CAG_score and multi-omics machine learning techniques, we developed a robust model that accurately predicts both prognostic risk and immunotherapy efficacy for BC study will contribute to enhancing the rationalization of immunotherapeutic approaches in breast cancer.

## 2 Materials and methods

### 2.1 Mendelian randomization analysis

To assess the potential connection between cholesterol and the risk of breast cancer, genetic data on cholesterol (met-a-307, sample Size 7,813, number of SNPs 2545,608)and breast cancer(ieu-a-1132, ER+ Breast cancer (Oncoarray), sample size 833691, number of SNPs 10680275) were searched and obtained from the IEU Open GWAS project(https://gwas.mrcieu.ac.uk/). The data then were briefly collated and subjected to a two-sample Mendelian randomization (2-SMR) analysis. Mendelian randomization-Egger (MR-Egger) method analyses were the main way performed along with the inverse variance-weighted (IVW) method analysis, Weighted-median method analysis, Weighted mode method analysis and Simple mode method analysis (12).

### 2.2 Download of the BC dataset and acquisition of cholesterol homeostasis-associated genes

The basic information on breast cancer RNA sequencing transcriptome data, CNV files, somatic mutation data, and clinicopathologic data were acquired from the publicly available TCGA database (http://xena.ucsc.edu/). Microarray dataset GSE58812 was downloaded from the GEO database (https://www.gov/geo/). A total of 1324 breast cancer samples were analyzed in this study. 1097 patients with a survival time greater than 30 days and 120 normal tissue samples were selected from the TCGA-BRCA cohort. The GSE58812 cohort contains 107 samples of breast cancer patients. The 73 Cholesterol homeostasis genes (CHGs) and 36 Angiogenesis genes (AAGs) were retrieved from the MSigDB team (Hallmark Gene set) as indicated in **Table S1**.

### 2.3 Consensus clustering analysis of CHGs

9 CHGs were obtained with univariate Cox regression (UniCox) analysis. Consensus clustering was used to identify different cholesterol homeostasis-related patterns by the k-means algorithms with 1000 repetitions (13). The distinction in clinical characteristics between the C1 and C2 groups was assessed using a Chi-square test. Differences in the biological function of these patterns were investigated using Genetic Set Variable Analysis (GSVA) (14). OS time and OS state of various modes were compared using the Kaplan-Meier method (15). Additionally, we explored the association between molecular patterns of cholesterol homeostasis genes, clinical features, and survival differences.

### 2.4 Landscape of tumor immune environment in different subgroups of breast cancer

The “Estimation” R package was used to present the proportion of immune cells and stromal cells in BC by analyzing gene expression, which can further calculate the tumor purity (16). Abundance of 23 specific immune cell subtypes was measured in tumors with the CIBERSORT algorithm to reveal the infiltration of immune cells (17). We predicted the sensitivity of immunotherapy by comparing the expression levels of several immune checkpoints among different subgroups. Moreover, the degree of immune cell infiltration in tumor and normal samples was determined by single sample Gene Set Enrichment Analysis (ssGSEA analysis) (18).

### 2.5 Identification of DEGs and cholesterol homeostasis-related genes

Using the “limma” package, we acquired DEGs for breast cancer in the TCGA dataset. DEGs should comply with the | log2 fold change (FC) | ≥ 0.5, p< 0.05. Pearson correlation analysis was used to obtain genes that were related to Cholesterol homeostasis, with |cor|≥ 0.6.

### 2.6 Prognostic score of cholesterol homeostasis

A CAG_Score was established to quantitatively evaluate the state of cholesterol homeostasis for individual BC patients. Firstly, we performed uniCox analysis and multi-factor Cox analysis (mulCox) for CHG-related genes to search for which has significant prognostic value. Then, we integrated OS time, OS, and gene expression data with the “glmnet” package and developed the CAG_Score by the Lasso Regression Algorithm (19).


$$\text{CAG score}=\;\sum_{\text{n}}\text{Coefficient of gene(n) }\times \text{ Expression of gene (n)}$$

The median CAG_score was adapted to classify breast cancer patients into low-risk and high-risk groups.

### 2.7 Construction of cholesterol homeostasis relevant nomograph

A CAGs-related nomograph was established to describe the clinical features and risk score of BC patients, as well as the clinical prediction of 3-year,4-year, and 5-year survival status. Calibration curves were generated to identify the accuracy of the predictive effect.

### 2.8 Drug sensitivity analysis and quantitative RT-PCR

The IC50 of commonly used clinical drugs was numerically analyzed by the “pRRophic” package in order to compare the chemotherapy effects of different risk groups (20). Total RNA of breast cancer cells (MDA-MB-231, MCF-7, SKBR-3) and normal breast cells (MCF-10A) were prepared by TRIzol reagent (Thermo Fisher Scientific, Waltham, USA). cDNA was synthesized with TOROIVD qRT Master Mix kit (TOROIVD, shanghai, China) according to the manufacturer’s instructions. The qRT-PCR was performed using the TOROGreen qPCR Master Mix kit (TOROIVD, shanghai, China) on the ABI 7500 real-time fluorescence quantitative PCR system (Thermo Fisher Scientific). All sequences of primers used are shown in **Table S2**.

### 2.9 Development of a multi-omics machine learning model to predict the prognosis and microenvironment of breast cancer

The TCGA cohort was divided into a training cohort (n=824) and a test cohort (n=206) randomly. We defined BC prognostic risk markers as characteristic mRNA, lncRNA, and miRNA in the TCGA cohort. Screening for characteristic mRNAs, miRNAs, and lncRNAs based on high-risk score and low-risk score, for each type of data, the top 100 most relevant features were retained as BC-specific risk markers according to the P-value. Then, we performed lasso regression for further feature filtering and reduced the number of markers to 20 for each type of data. With 20 makers per molecular layer, we created a risk predictor of each single molecule layer with three machine learning models, such as Light GBM, Logistic regression, and Random forest (21). Finally, based on 60 BC-specific markers from three data types, we developed a LightGBM model (RiskLight) to distinguish breast cancer patients with different prognostic risks associated with dysregulated cholesterol homeostasis.

### 2.10 Statistical analysis

In the statistical analysis, p<0.05 was considered statistically significant. The t-test is used for the analysis of normally distributed data, while the Wilcoxon rank sum test is used for the analysis of abnormally distributed data. In addition, Pearson correlation analysis or Spearman analysis was used to describe the relationship between two numerical variables. The above algorithms are all implemented in R Software (version 4.1.2).

## 3 Results

### 3.1 Clinical and mutations data of CHGs in BC

**Figure 1:**
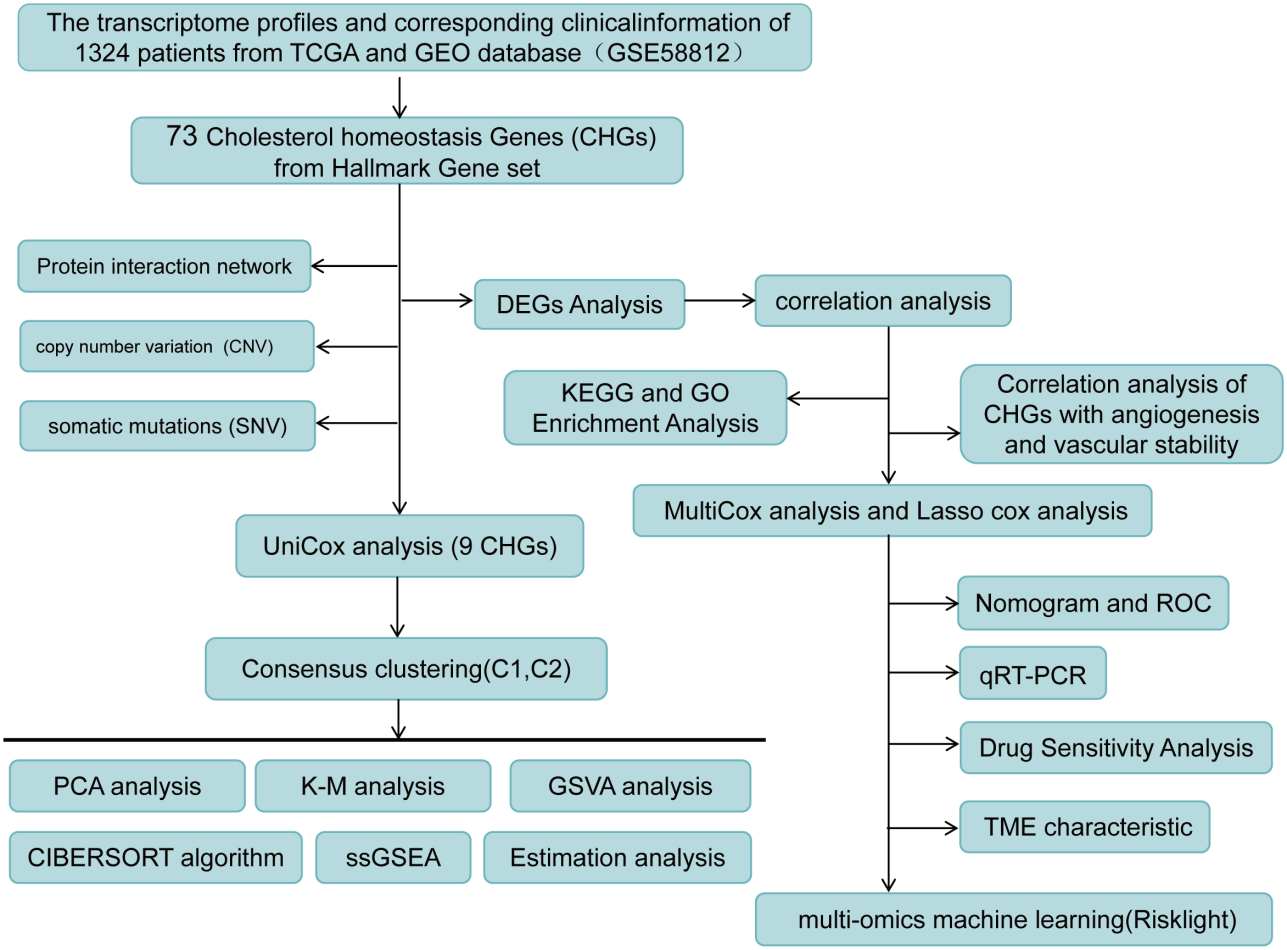
Flow chart of research design.

**Figure 2:**
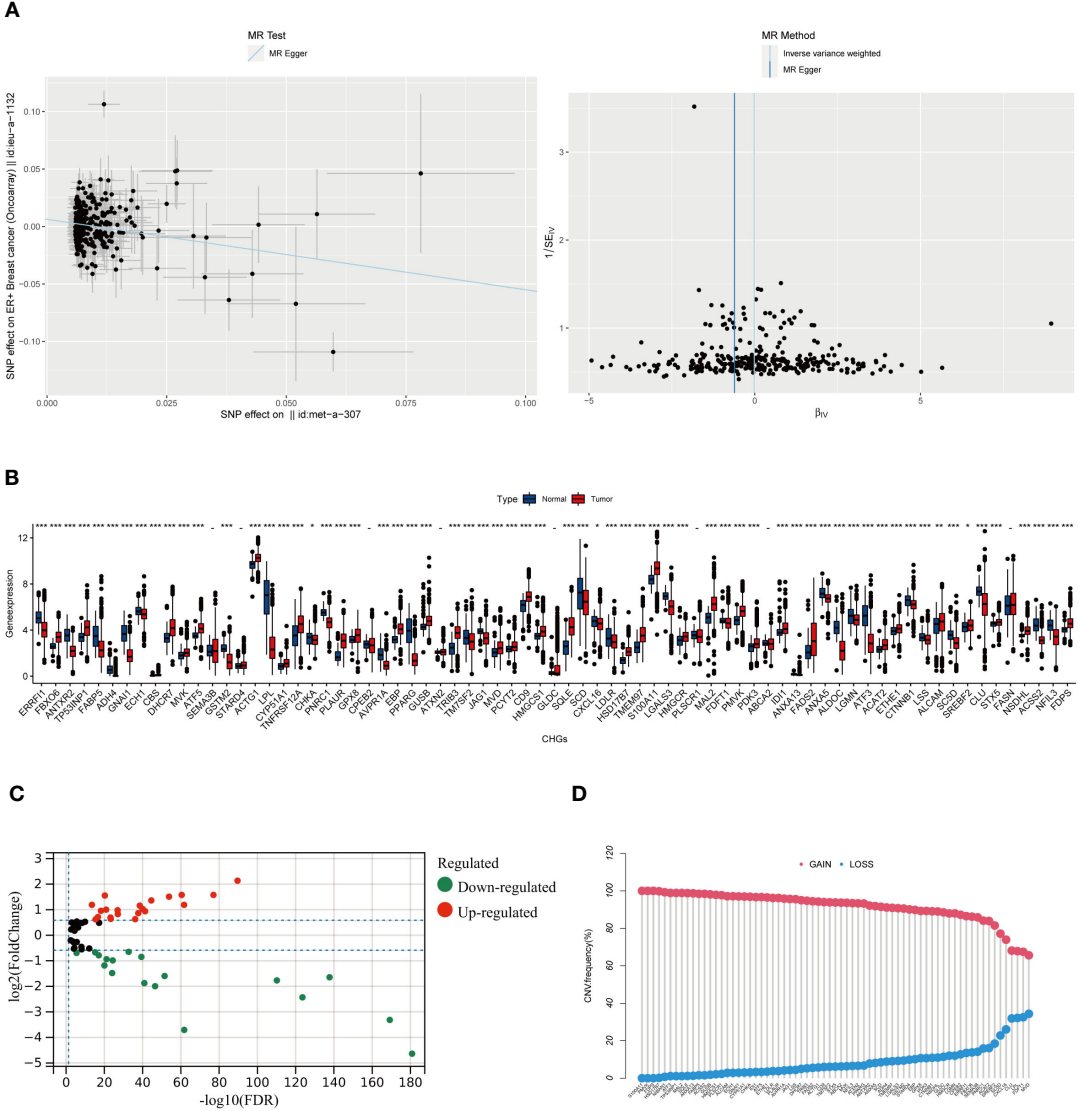
The result of Mendel randomized model and the Molecular Characteristics of CHGs in BC. **(A)** Association between cholesterol and ER+ breast cancer risk overall.MR-analyses are derived using random effect Ivw, MR-Egger, weighted median and mode.(MR Egger, OR: 0.54, 95% CI: 0.35-0.84, p<0.006). **(B)** Distribution of CHGs between BC and normal tissues. (p>0.05 -; p< 0.05 *; p< 0.01 **; p< 0.001 ***). **(C)** Volcano map of 63 DEGs (log2 fold change. (FC)|≥0.5, p-value<0.05). **(D)** Incidence rate of CNV gain, loss, and non-CNV among CHGs.

The flow chart of the research design is shown in **Figure 1**. To evaluate the role of cholesterol in the occurrence of breast cancer, the Mendelian randomisation-Egger (MR-Egger) method was used first in the main MR analysis, as the detailed results are presented in **Figure 2A** (MR Egger, OR: 0.54, 95% CI: 0.35-0.84, p<0.006). This means that cholesterol levels may be a risk factor for breast cancer. We obtained 73 genes for cholesterol homeostasis from the MSigDB database and verified the expression levels of 73 CHGs in tumor specimens and normal control in the TCGA-BC cohort (**Figure 2B**). 63 CHGs had differential expression (**Figure 2C**). Correlations between 73 CHGs were analyzed with the String website (**Table S3**). Protein interaction network (PPI) was constructed by Cytoscape software to explore the interactions between CHGs (22). And we identified SCD, PPARG, CTNNB1, FDPS, LDLR, ACSS2, FDFT1, FADS2, HMGCR, SREBF2, ACTG1 and HMGCS1 as the vital genes of cholesterol homeostasis (**Figure S1**). We calculated the CNV mutation rate of CHGs, **Figure 2D** shows the results. In addition, we determined the incidence of SNV of 73 CHGs in BC, and 142 out of 981 BC samples (14.46%) showed mutations, which indicated that the mutation rate of 73 CHGs was less than 1% (**Figure S2**).

### 3.2 Generation of cholesterol homeostasis subgroups in BC

**Figure 3:**
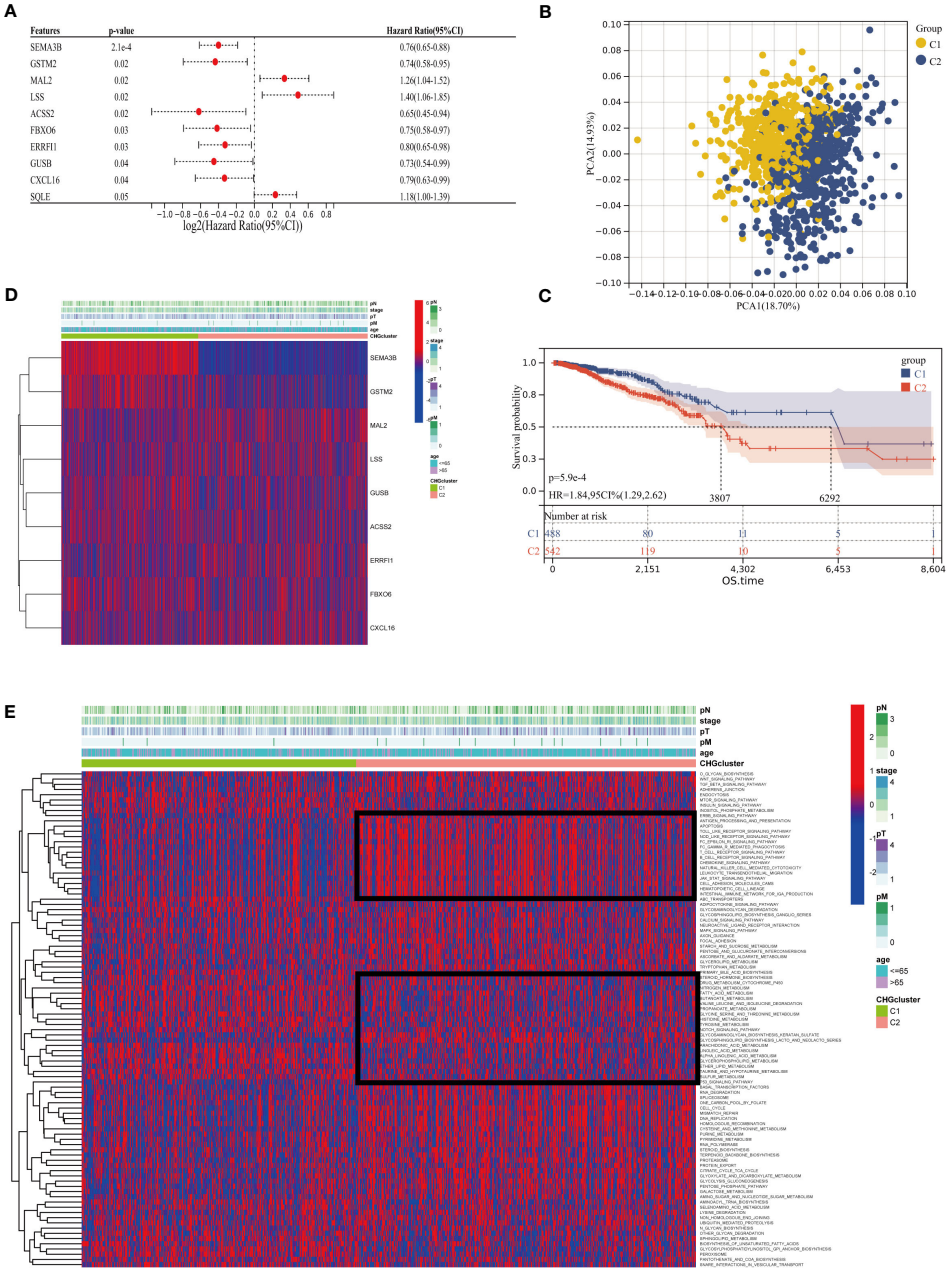
Cluster analysis of cholesterol homeostasis subgroups. **(A)** Univariate Cox regression (uniCox) analysis for CHGs (p<0.05 is considered significant). **(B)** PCA analysis showed the distribution of the two clusters. **(C)** Survival curve between different clusters. **(D)** Expression of prognostic genes and the presentation of clinical features in different clusters. **(E)** The heatmap of biological function and signaling pathway in two groups.

Generation of a subset of genes related to cholesterol homeostasis regulation in BC to reveal the relationship between cholesterol homeostasis regulation and tumorigenesis. 1097 BC patients of TCGA-BC were included in this study, and uniCox analysis revealed 9 CHGs with prognostic significance (**Figure 3A**). To determine the relationship between CHGs expression patterns and BC subtypes, consensus cluster analysis was used to classify BC patients according to prognostic genes. When the clustering variable was 2, BC patients were well divided into the C1 group (n=510) and the C2 group (n=587) (**Figure S3**). PCA analysis showed significant subpopulation differentiation in samples (**Figure 3B**). KM analysis revealed that cluster C2 showed a worse prognostic status (**Figure 3C**).The clinical features distinguishing the C1 and C2 groups are presented in **Table S4**. In addition, the relationship between gene expression and clinical features of the two clusters was shown in **Figure 3D**. The heatmap indicated that the expression level of CHGs had a significant correlation with the clinical characteristics, and the genetic characteristics of the C2 subcluster were associated with distant tumor metastasis. The biological functions and signaling pathways of tumor cells were compared by the GSVA algorithm, and the findings showed that the C2 subcluster performed obvious immune pathway characteristics, lipid metabolism, and sterol metabolism-related pathways were down-regulated, and cancer metastasis-related pathways were significantly different as well (**Figure 3E**). This suggests that dysregulation of cholesterol metabolism is closely associated with tumor immunity and the development of tumors.

### 3.3 Characteristics of the TME in different subgroups

**Figure 4:**
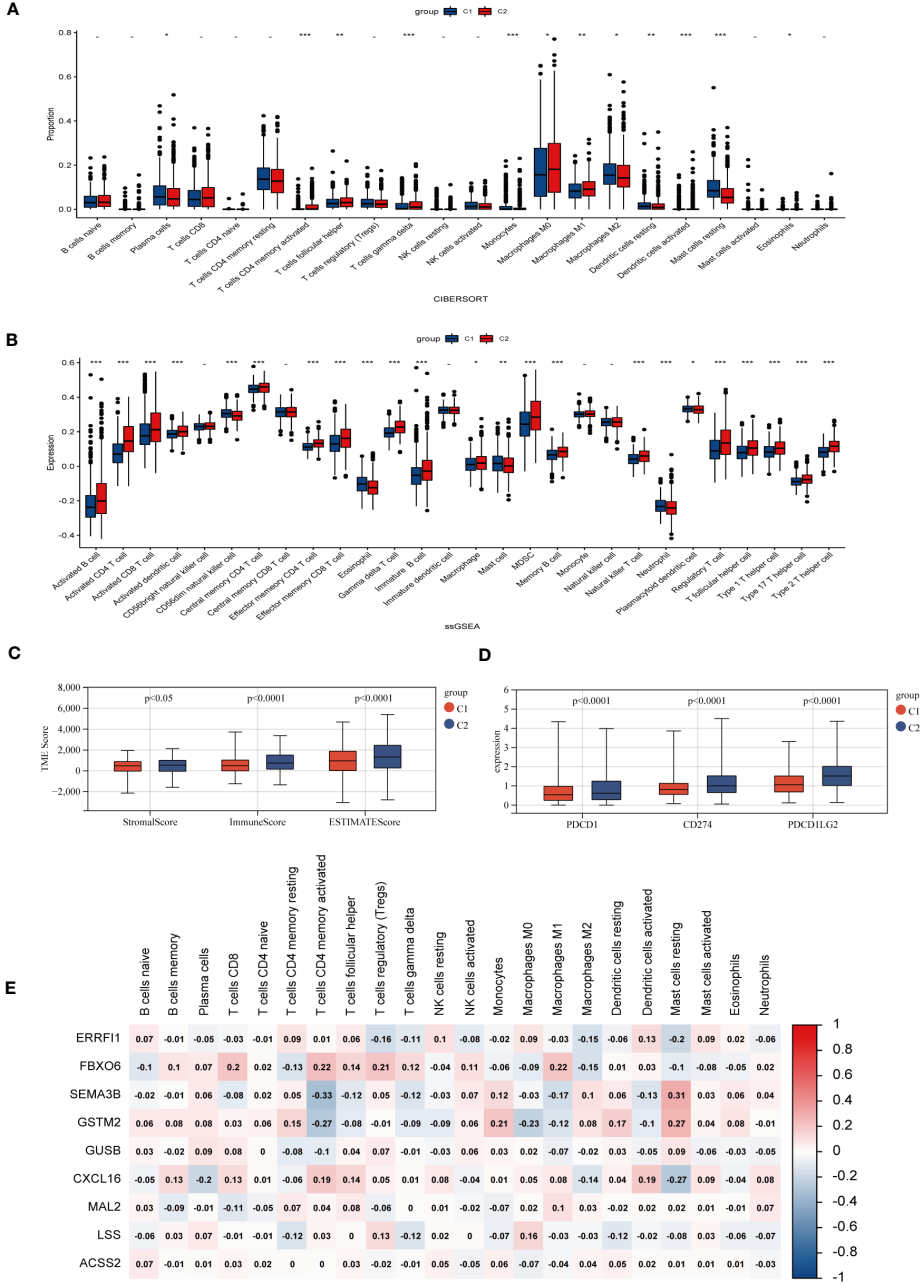
Characteristic of TME in two BC subgroups. **(A)** Abundances of 23 infiltrating immune cells in two BC subpopulations. **(B)** Enrichment score of 23 immune cells for each BC sample by ssGSEA analysis. (p>0.05 -; p< 0.05 *; p< 0.01 **; p< 0.001 ***). **(C)** Expression levels of immune checkpoints (PD-1, PD-L1, and CTLA-4) of different subgroups. **(D)** Immune infiltration scores for different groups. **(E)** Correlation of clustering genes with 23 immune cells.

Investigating the infiltration extent of 23 human immune cells in both clusters by the CIBERSORT algorithm (**Figure 4A**), we found that the content of Macrophage M0, Macrophage M1, activated Dendritic cell and T cell include activated CD4 positive memory T cell, helper T cell, gamma and delta T cell were significantly higher in group C2, whereas Plasma cell, macrophage M2, resting dendritic cell mast cells behaved in an opposite way. Inter-individual differences in 23 immune cells were assessed by the “ssGSEA” algorithm and the number was generally higher in the C2 group (**Figure 4B**). The TME scores exhibited that patients in cluster C2 had a higher abundance of immune and matrix components (**Figure 4C**). In addition, PD-1, PD-L1, and CTLA-4 were shown a similar increase in cohort C2, which represents the critical expression status of the immune checkpoints (ICP) (**Figure 4D**). Meanwhile, the correlation analysis between CHGs and immune cells displayed that FBXO6, SEMA3B, GSTM2, and CXCL16 were correlated with immune cell abundance (**Figure 4E**).

### 3.4 Potential biological activity of cholesterol homeostasis gene, correlation analysis between CHGs and angiogenesis

**Figure 5:**
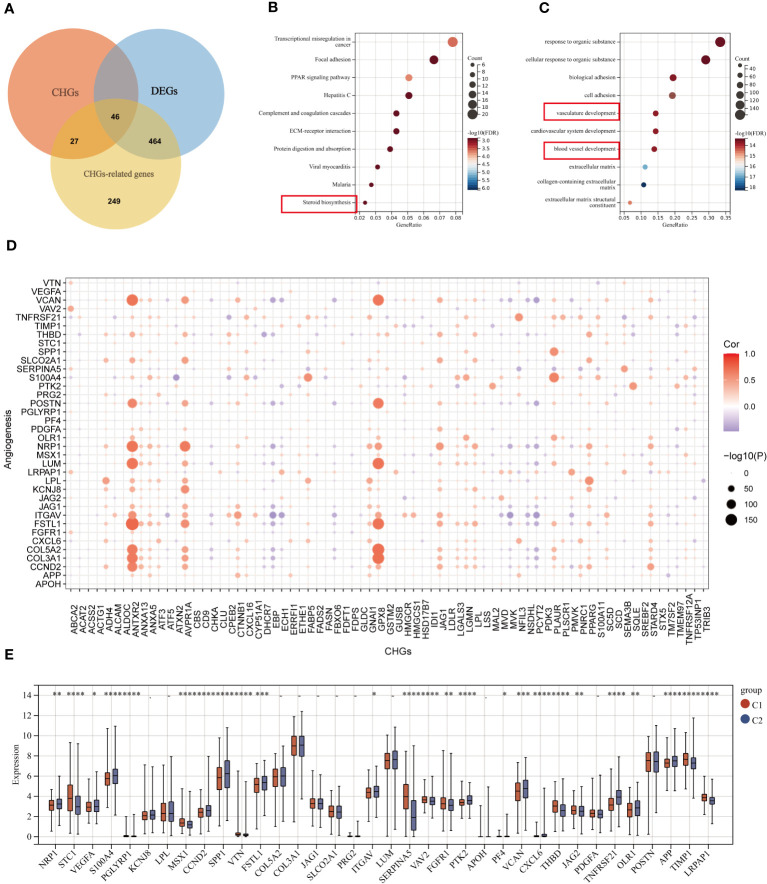
Correlation analysis of CHGs and angiogenesis. **(A)** Acquisition of cholesterol homeostasis-related DEGs (log2 fold change (FC)|≥0.5, p-value<0.05). **(B, C)** GO and KEGG enrichment analyses of cholesterol homeostasis-related DEGs among two subgroups. **(D)** Correlation analysis of CHGs and AAGs. **(E)** Expression levels of 36 AAGs between C1 and C2. *p < 0.05; **p < 0.01; ***p < 0.001; ****p< 0.0001.

The Pearson correlation algorithm was applied to analyze CHGs, resulting in 510 highly correlated DEGs (**Figure 5A**). Functional enrichment analysis of these DEGs was then performed to demonstrate the potential biological activity of cholesterol homeostasis genes. KEGG and GO analysis revealed an enrichment of cancer and metastasis-related pathways as well as blood vessel development and sterol metabolism, which suggested that cholesterol homeostasis is closely related to angiogenesis (**Figures 5B, C**). To reveal the association between cholesterol homeostasis and angiogenesis, we obtained 36 angiogenic genes (AAGs) from MsiGDB and explored the correlation between CHGs and AAGs. The results were as expected, especially when ANTXR2, GPX8, and AVPR1A were strongly associated with angiogenesis (**Figure 5D**).

**Figure 6:**
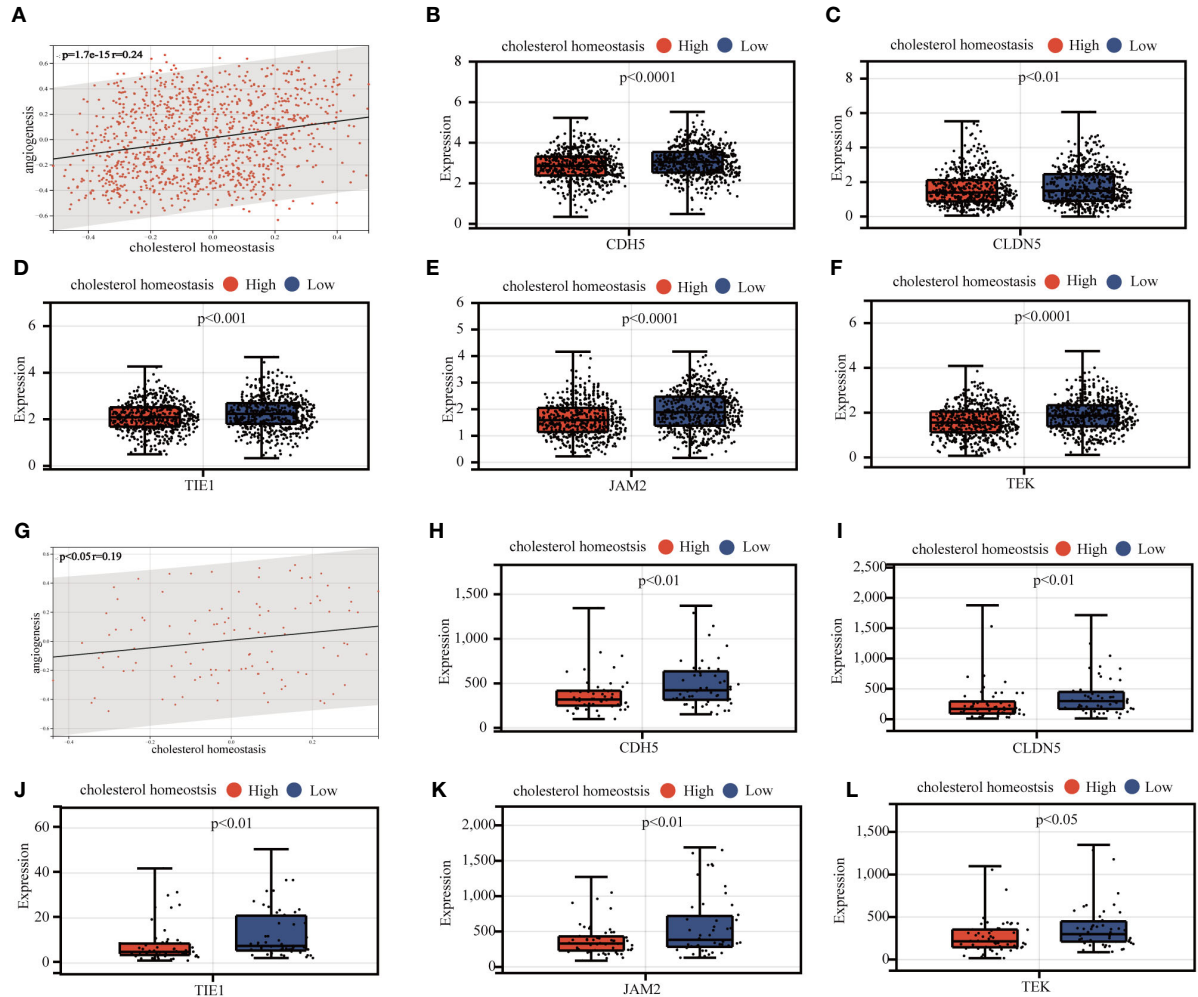
Analysis of the correlation between cholesterol homeostasis and vascular stability. **(A)** Association analysis of cholesterol homeostasis score and angiogenesis score. **(B-F)** Association between expression levels of vasostability genes and cholesterol homeostasis scores. **(G-L)** Validation of the above results in the GEO 58812 queue.

Subsequently, we examined the expression of AAGs in groups C1 and C2 (**Figure 5E**), as well as in tumor and normal tissues (**Figure S4**); however, a significant discrepancy exists. The GSVA algorithm was used to evaluate the cholesterol homeostasis score (CHG score) and angiogenesis score (AAG score) of TCGA BC samples based on 73 CHGs and 36 AAGs. And cholesterol homeostasis scores were positively correlated with angiogenesis scores in the TCGA-BC cohort (**Figure 6A**). Moreover, the cholesterol homeostasis score and angiogenesis score were compared between the C1 and C2 groups. We found that patients in the C2 group had a worse prognosis with higher cholesterol homeostasis score and angiogenesis score (**Figure S5**). The correlation between vascular stability and cholesterol homeostasis score was also validated in addition. The abundance of genes related to vascular stability (CDH5, CLDN5, TIE1, JAM2, TEK) indicated that the group with a lower cholesterol score had higher vascular stability (**Figures 6B-F**), while low vascular stability often promotes cancer growth (23–27). All the findings were verified in the GSE58812 cohort (**Figures 6G-L**).

### 3.5 Development and validation of the prognostic CAG_score

**Figure 7:**
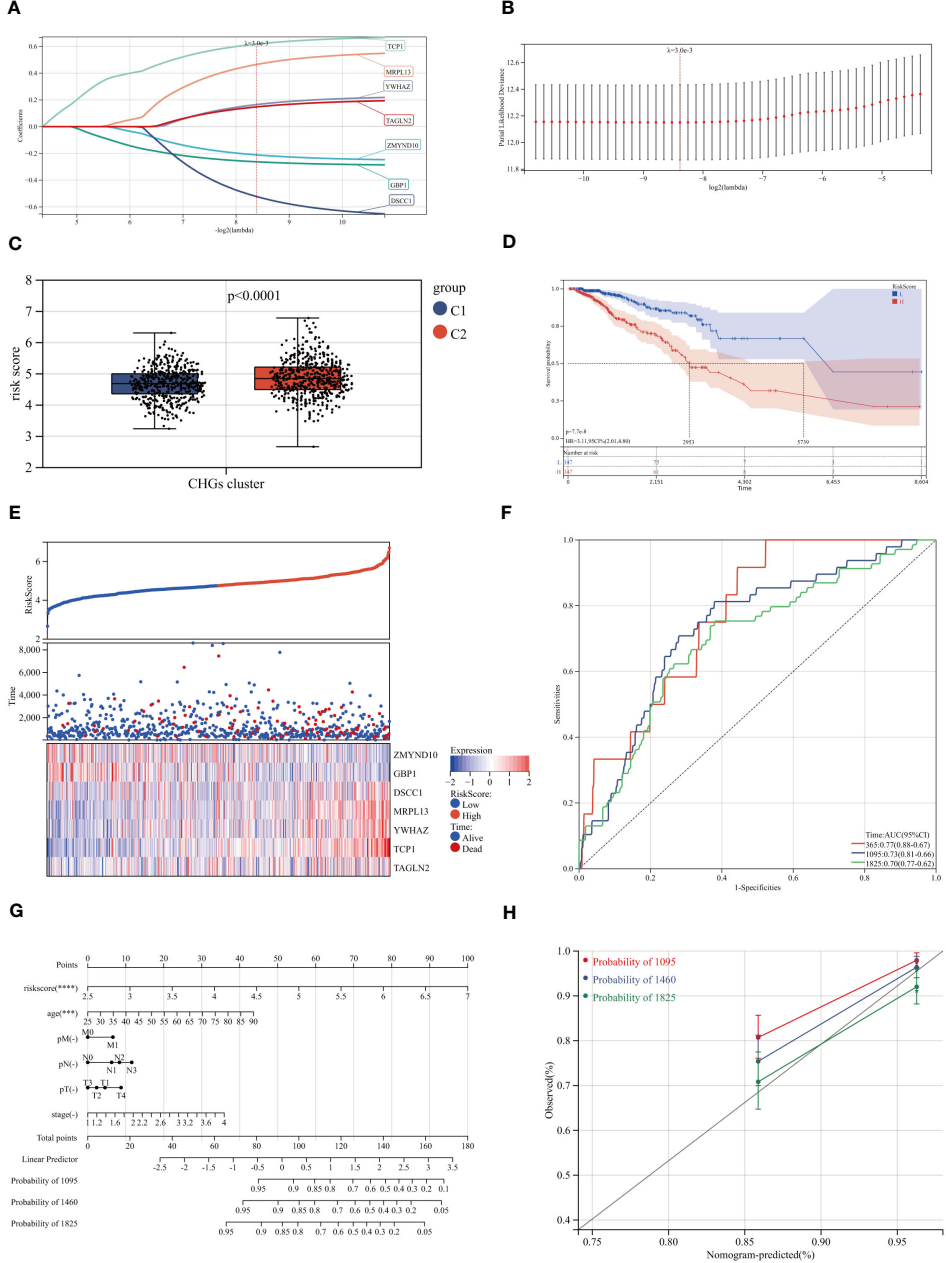
Construction of the CAG_score. **(A, B)** The LASSO analysis and determine the optimal LASSO settings. **(C, D)** Survival curves for groups and comparison of risk scores for different clusters. **(E)** Distribution of the risk score and BC patients. Dot plot of survival status. Heat maps of 7 cholesterol homeostasis-related gene expression of high- and low- risk groups. **(F)** ROC curve of the training group, the AUC values of 1, 3 and 5 years were 0.77, 0.73 and 0.70, respectively. **(G)** A nomogram for predicting the 3-, 4-, and 5-year OS for BC patients in TCGA cohort. **(H)** Calibration curves of the nomogram.


Considering that cholesterol homeostasis is closely connected with angiogenesis, we developed a prognostic CAG_score based on genes related to cholesterol homeostasis. The BC patients were randomly assigned to the training cohort (n=731) or the test cohort (n=366). We performed UniCox analysis of 786 cholesterol-related genes, and 49 DEGs with prognostic significance (logFC>0.5, P<0.05). Subsequently, LASSO and multi-Cox analyses were performed on 49 DEGs to establish the most suitable prediction model. We set the Lambda value to 0.00298971135072249 and finally obtained 7 genes (**Figures 7A, B**).

$$\begin{array}{l} \text{CAG\_score}=-0.21106035029347*\text{ZMYND}10-0.262724856118755*\text{GBP}1-\\ 0.522741360511683*\text{DSCC}1+0.465453655395411*\text{MRPL}13+0.16530191756177*\\ \text{YWHAZ}+0.617851278765801*\text{ TCP}1+0.147920101131816*\text{ TAGLN}2 \end{array}$$

In the scoring model established by CAGs, we found that higher scores were associated with a worse survival rate and higher mortality rate (**Figures 7C, D**). The model genes also showed a trend with increasing CAG_score (**Figure 7E**). To evaluate the robustness of the CAG_score, we compared the CAG_score from the test to the whole cohort, and the results showed an excellent performance of the CAG_ score in assessing the prognosis of BC patients (**Figures 7F**, **S6**). **Figure S7** shows the distribution of CHGs and AAGs in the two CAG_score clusters. We found significant differences in gene expression in both groups.

### 3.6 Construction of a nomogram to predict patient prognosis

Through the analysis of clinical indicators, we established a nomogram to predict 3, 4, and 5-year OS in BC patients (**Figure 7G**). The calibration curve shows that the method has a high forecasting accuracy (**Figure 7H**). Meanwhile, the R package “Rms” was conducted to integrate data on survival time, survival status, and 6 characteristics, and a nomogram was built using the Cox method to assess the prognostic value of these characteristics in a sample of 1030. The overall C-index of the model was: 0.783437290915762, 95%CI(0.740754644512089-0.826119937319435), p value=1.00165584281093e-38.

### 3.7 Assessment of TME characteristic in different groups

**Figure 8:**
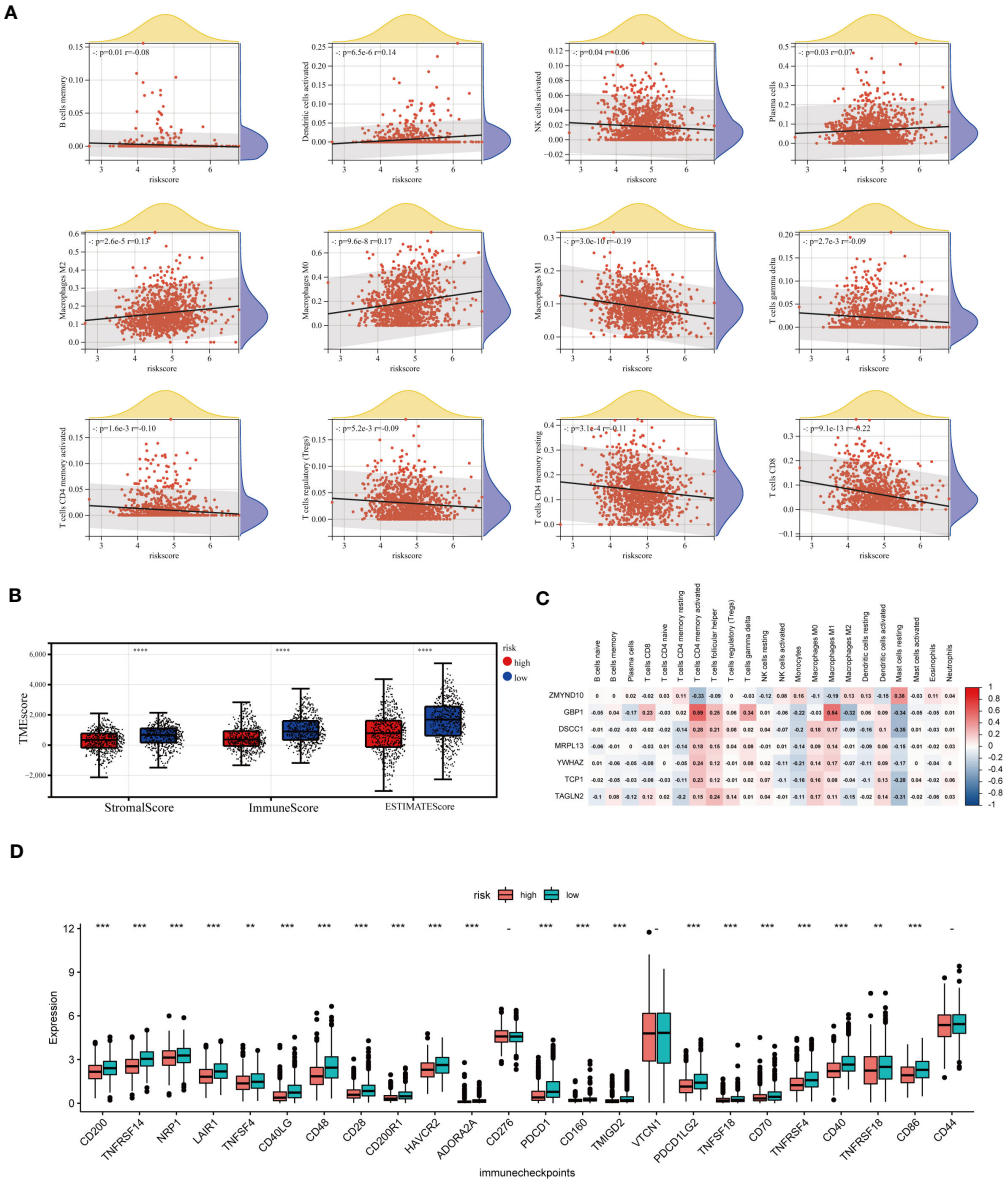
TME analysis of different risk score groups. **(A)** Correlations between CAG_score and immune cell types. **(B)** Immune infiltration scores for different groups. **(C)** Association of prognostic model genes with 23 immune cells. **(D)** Expression levels of 24 immune checkpoints in different subgroups. **p < 0.01; ***p < 0.001; ****p< 0.0001.

As mentioned above, CAG_score was positively correlated with the abundance of Macrophage M0, Macrophage M2, Plasma cell, and activated Dendritic cell, while CD8+ T cell, T cell gamma delta, activated or dormant CD4+ memory T cell, B memory cell, regulatory T cell, activated NK cell, macrophage M1 were negatively correlated with CAG_score (**Figure 8A**). In addition, there was a direct correlation between the CAG_ score and the TME score (**Figure 8B**). We explored the relevance between prognostic marker genes and 23 immune cells. We concluded that T cells and Macrophages are closely associated with the selected genes (**Figure 8C**). Furthermore, We evaluated the expression of ICPs in groups of different prognostic features. **Figure 8D** shows that the expression of 24 ICPs was inconsistent in both risk subgroups. The low-risk group showed a higher level of ICPs expression.

### 3.8 Drug sensitivity analysis

**Figure 9:**
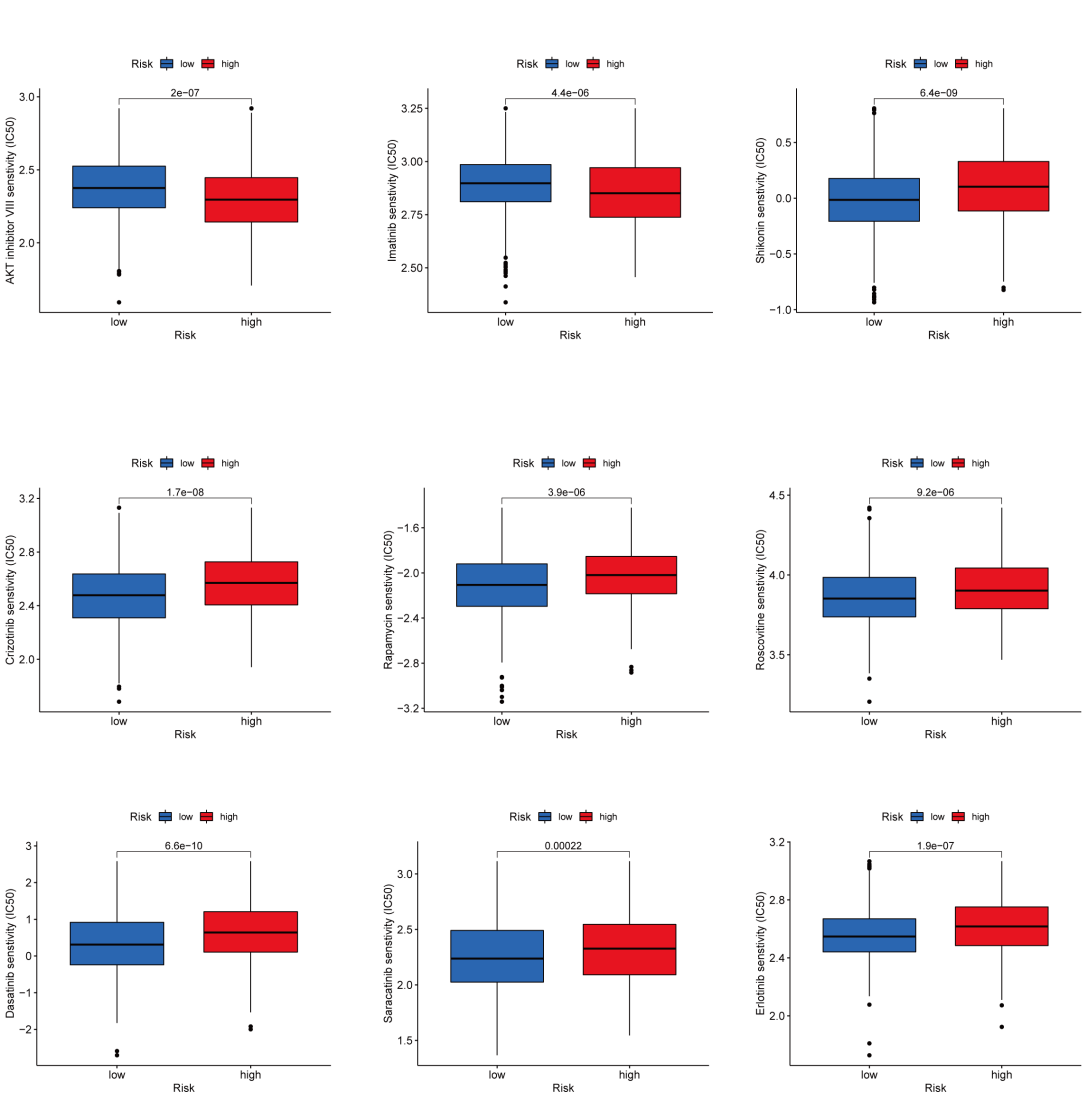
Drug Sensitivity Analysis Prediction of clinically common drug susceptibility in patients with different CAG scores.

It is a meaningful research direction to select and guide the appropriate immunotherapy regimen for the patient (28). To examine the role of CAG_scores in clinical diagnosis, we evaluated the IC50 for 138 Common drugs in TCGA-BC patients. The results showed that BC patients with higher CAG_ scores were more sensitive to the AKT inhibitors VIII and Imatinib, while patients with low CAG_ scores responded better to Crizotinib, Saracatinib, Erlotinib, Dasatinib, Rapamycin, Roscovitine and Shikonin (**Figure 9A**).

### 3.9 The results of qRT-PCR in several breast cancer cell lines

**Figure 10:**
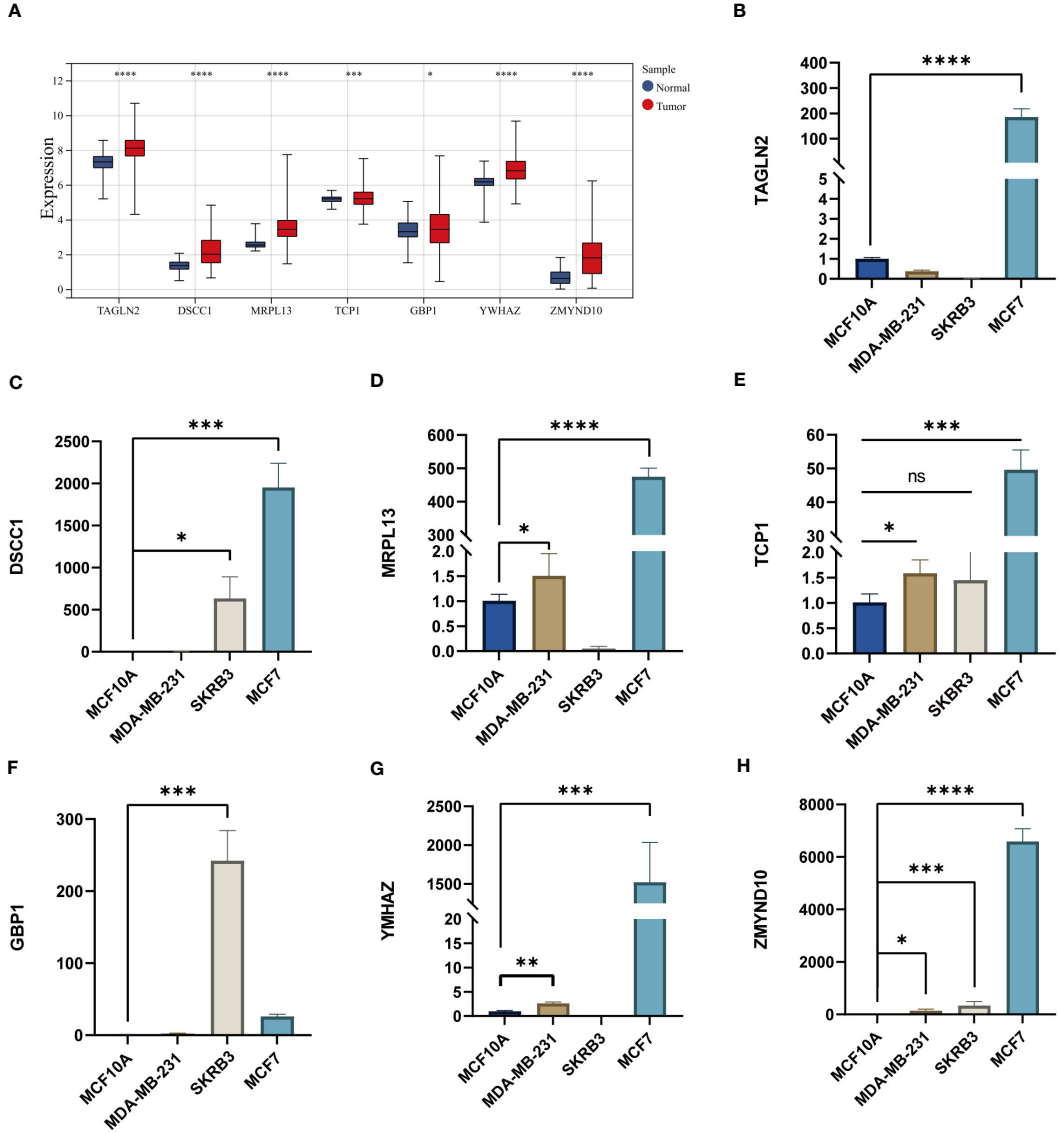
**(A)** The expression levels of CAG_score genes in TCGA BC cohort. **(B-H)** The mRNA levels of CAG_score genes in breast cancer cell lines. (*P <0.05, **P <0.01, *** P < 0.001, **** P < 0.0001; ns, nonsignificant).

We detected the RNA expression of the CAG_score genes in breast cancer cell lines. Our results indicate that all genes were highly expressed in MDA-MB-231, MCF-7 and SKBR-3 cell lines (**Figures 10A-H**), which was consistent with our prediction.

### 3.10 Multidimensional data features for different risk groups and multi-omics machine learning to build prognostic models

**Figure 11:**
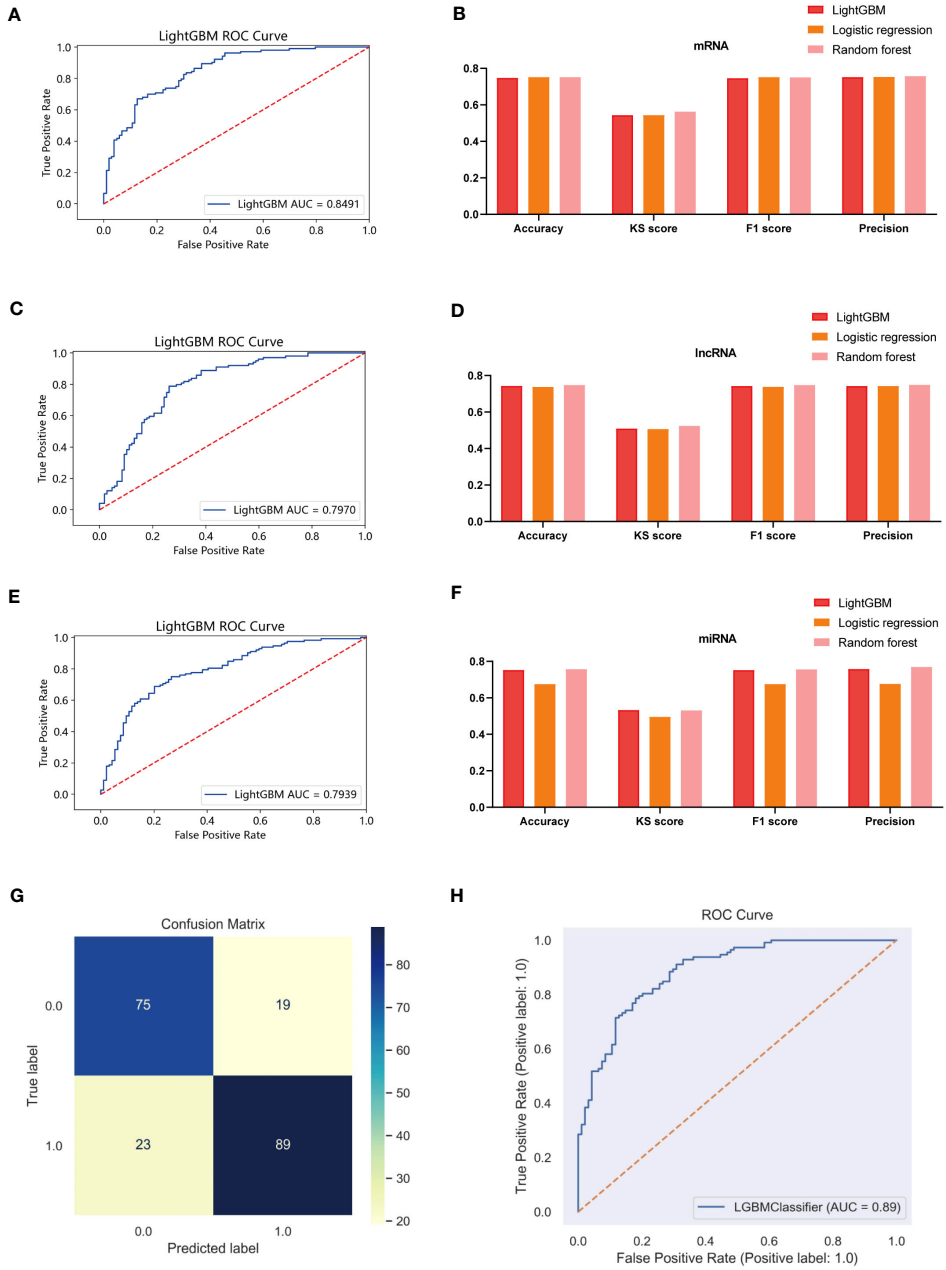
Multi-model comparison based on single molecular plane of characteristic mrna, mirna and lncrna. **(A)** ROC curve of mRNA risk model (test set, n=380). **(B)** Accuracy, KS score, F1 score and Precision of mRNA different risk model. **(C)** ROC curve of lncRNA risk model (test set, n=380). **(D)** Accuracy, KS score, F1 score and Precision of lncRNA different risk model. **(E)** ROC curve of miRNA risk model (test set, n=380). **(F)** Accuracy, KS score, F1 score and Precision of miRNA different risk model. **(G)** A confounding matrix for predicting patient prognostic risk with multi-omics data using the test set (N = 380) **(H)** ROC curve of Risklight (test set, n=380).

We have demonstrated the significance of metabolic regulation associated with cholesterol homeostasis as an immune micro-environmental factor in our study. To further identify molecular signatures associated with prognostic risk at the multi-omics level, we conducted an analysis of associations between three molecular layers (mRNA, miRNA, lncRNA) and high-low risk for each type of data, the top 100 most relevant features were retained as BC-specific risk markers according to the P-value (**Figure S8A**). We used the Light GBM framework to integrate multi-omics features to develop high- and low-risk prediction models as a way to emulate the tumor micro-environment in which cholesterol homeostasis is dysregulated. As a result, the three risk predictors based on the single molecular layer performed well in predicting high and low risk in the test cohort (AUC=0.8491 for the mRNA model, AUC=0.7939 for the lncRNA model, and AUC=0.7970 for the miRNA model) (**Figures 11A, C, E**). We also compared the superiority of random forest and logistic regression models with the Lightgbm model (**Figures 11B, D, F**). The results show that all three algorithms exhibit consistent results. Finally, we integrated 3 risk predictors, based on the LightGBM algorithm combined with multi-omics data to develop an integrated model (Risklight) for predicting cholesterol homeostasis-related risk patterns. Risklight is superior to all risk predictors based on single molecular layers (AUC=0.89) (**Figures 11G, H**).

## 4 Discussion

Locoregional and systemic therapies of BC have progressed substantially over the past years, at the same time, precision treatment has become a major focus on the treatment of BC. Since the importance of developing effective therapies has been noticed, it is still necessary to define the risk factors of BC and exploit this information to formulate chemopreventative strategies and improve lifestyles that can help to reduce the burden of BC. Although the results of our MR analysis suggest that cholesterol levels are a risk factor for BC, the exact mechanism of its occurrence remains unknown. It is still necessary to investigate the characteristics of cholesterol homeostasis genes and their potential biological activity in BC.

Our study quantified the cholesterol score of each BC patient’s sample by utilizing a set of cholesterol homeostasis genes and evaluated different patterns of cholesterol homeostasis in BC. It showed significant differences in immune infiltration, functional enrichment, and clinical outcomes in different cholesterol gene expression pattern groups. ACT and ICI therapies, as we all know, are the success of cancer immunotherapy (29). There is no doubt about that that immune cells, particularly T cells, can be harnessed to eliminate tumor cells (30). The presence of TIL, especially CTL, is positively correlated with the survival rate of various cancer patients (31). Unexpectedly, despite having higher levels of CD8+T cells, including CTL, the C2 cluster exhibited a poorer prognosis and stronger features of distant tumor metastasis with downregulation of multiple metabolisms including sterol metabolism and fatty acid metabolism. Previous researches show that cholesterol metabolism plays a critical role in activation, proliferation, and effector function of CD8+ T cell (32). We imply that the downregulation of sterol and lipid metabolism reduces the effector function of CTL, making the C2 subgroup have a poorer prognosis with high levels of immunity levels (33). Not surprisingly, the C1 subpopulation has a well-prognostic with low levels of immune under high levels of sterol and lipid metabolism. Moreover, cholesterol homeostasis genes FBXO6, SEMA3B, GSTM2 and CXCL16 were associated with immune cell abundance. In previous studies, FBXO6 and CXCL16 have been shown to be directly related to immunity. Specifically, FBXO6 has been shown to impair the survival of alveolar macrophages by enhancing the degradation of NLRX1 (34), while CXCL16 serves as a critical ligand for CXCR6 that promotes the survival and local expansion of effector CTL in the TME (35). However, the exact role of SEMA3B and GSTM2 in the immune environment is unclear and requires further elucidated. These findings suggest that targeted regulation of cholesterol homeostasis may be a novel approach new immunotherapy in BC.

In addition, cholesterol homeostasis genes (CHGs) exhibit a strong correlation with the development of vasculature. Tumor growth necessitates neovascularization to adequately supply rapidly proliferating tumor cells with oxygen and nutrients (36).. Our study highlights the robust association between angiogenic genes and three specific CHGs: GPX8, ANTXR2, and AVPR1A. Glutathione peroxidase 8 (GPX8) has been demonstrated as crucial for maintaining the invasive phenotype in breast cancer (37); however, direct evidence linking GPX8 to breast cancer tumor angiogenesis is currently lacking. Hence, further investigation is warranted to explore the involvement of GPX8 in angiogenesis within breast cancer. ANTXR2 is a type I membrane protein participant in extracellular matrix homeostasis (38). Cholesterol depletion induces ANTXR2-dependent activation of MMP-2 in glioma cells (39). Down-regulation of ANTXR2 expression inhibits proliferation and capillary network formation in human umbilical vein endothelial cells (HUVECs) (40). Vasopressin receptor 1A (AVPR1A) serves as a pivotal receptor for vasoconstriction (41). Notably, patients with higher CHG expression scores showed reduced expression of the cluster vascular stability genes JAM2, CDH5 (VE calcineurin), CLDN5 (Claudin 5), TIE1, and TEK (TIE2).Under normal conditions, the endothelium of mature capillaries is quiescent, stable, and limits vascular leakage (42). Genetic deletion of JAM2, CLDN5, and CDH5 significantly increases vascular permeability and leads to vascular barrier dysfunction (43). Angiopoietin-1 (Ang-1) acts through its receptors TIE1 and TEK (44), and deletion of TIE1 and TEK ultimately leads to reduced vascular stability (45). A number of pathologic disorders can lead to destabilization of the vascular network, resulting in hyperendothelial permeability, excessive vascular outgrowth and angiogenesis. In turn, overgrowth or aberrant remodeling of blood vessels promotes many diseases, including cancers (46). Abnormalities in the vasculature and the resulting microenvironment accelerate tumor progression and lead to reduced efficacy of chemotherapy, radiotherapy, and immunotherapy (47).Therefore, we need to focus on the in-depth link between cholesterol homeostasis and tumor angiogenesis, which may be a potential node for the treatment of breast cancer.

The development of risk stratification tools concerning cancer survivorship has become a priority for research in clinical practice. We developed a CAG_ score based on CHGs to predict prognostic risk and survival time in BC patients. In general, the higher the CAG_ score is, the worse the prognosis is, combined with a higher risk of death. The CAG_ score is related to the abundance of T lymphocytes (CD4^+^, CD8^+^ T) and Macrophages. CD4^+^ T cells regulate the immune response by producing cytokines (48). CD8^+^ T cells kill pathogens or produce inflammatory factors and cell division molecules (49). With the increase in risk score, the level of T cells showed a downward trend, which meant the level of immunity was decreased, and the patients were more vulnerable to tumors. In addition, the level of M1 macrophages decreased while the level of M2 increased with the increase of CAG_score. Tumor-associated macrophages (TAMs) are considered essential tumor-associated immune cells by promoting tumor growth, invasion, and metastasis, which contain two subtypes with separate functions (50). Typically activated M1 macrophages are known to reduce the survival of tumor cells through direct killing and antibody-dependent cell-mediated cytotoxicity (ADCC) (51).In contrast, M2 macrophages, as TAMs in a narrow sense, can inhibit the immune effect of T lymphocytes and promote tumor angiogenesis, leading to immune escape and tumor progression (52). The prediction of TAMs by the CAG_ score was completely consistent with tumor progression and clinical outcome, which means CAG_ score has a great ability to predict the status of TME in BC patients. Therefore, we recommend that risk stratification of cholesterol metabolism should be considered as a screening test for further investigation, intervention, and support of tumor.

Immune checkpoint inhibitors have been shown to be effective in the treatment of a variety of tumors (53). This can be observed a marked upregulation in the low CAG score group, suggesting that patients with low CAG scores may be more sensitive to immunotherapy. Currently, chemotherapy resistance in BC is getting worse (54). Our study also provides possible susceptibility drugs for patients with different CAG score groups, which could facilitate clinical selective medication.

Nevertheless, the molecular pathways involved in the development of BC have not been elucidated in detail. With multi-omics data exploration, molecular alterations in three different molecular layers (mRNA, miRNA, lncRNA) driven by the tumor microenvironment emerge in different patients. Combining with multi-omics features and the Risklight tool, we further developed a model of risk in BC patients associated with cholesterol homeostasis disequilibrium.

Overall, this study provides valuable insights on the prognosis of breast cancer patients. Reconsideration of alterations in cholesterol homeostasis as potential risk factors for tumor progression is warranted. The intricate relationship between functional changes in cholesterol homeostasis and tumor angiogenesis and immune response remains incompletelyated, necessitating further exploration of the association cholesterol homeostasis genes and angiogenesis as well as immune response. In especially, the roles of SEMA3B and GSTM2 in immunity should be further clarified. Notably, GPX8, a cholesterol homeostasis gene with unknown implications for angiogenesis but exhibiting a strong correlation with it, warrants thorough investigation. Additionally, a more robust prognostic model pertaining to cholesterol must be established, incorporating both the actual levels of patients’ cholesterol and those of cholesterol homeostasis genes. This will significantly enhance the accuracy of breast cancer prognosis models related to cholesterol, bringing them closer to clinical research and practice. Finally, we hope that the secrets of cholesterol homeostasis in breast cancer will increasingly be revealed. That’s why we started this study.

## Data availability statement

Publicly available datasets were analyzed in this study. This data can be found here: http://xena.ucsc.edu/.

## Ethics statement

Ethical approval was not required for the studies on animals in accordance with the local legislation and institutional requirements because only commercially available established cell lines were used.

## Author contributions

HW was the main contributor to the manuscript. All authors read and approved the final manuscript.
