# Supplementary figures and images for: Prognostic value analysis of cholesterol and cholesterol homeostasis related genes in breast cancer by Mendelian randomization and multi-omics machine learning
Source: Front Oncol. 2023 Nov 7;13:1246880. doi: 10.3389/fonc.2023.1246880 (PMC10661325; doi:10.3389/fonc.2023.1246880)

Risk 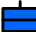 low 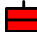 high

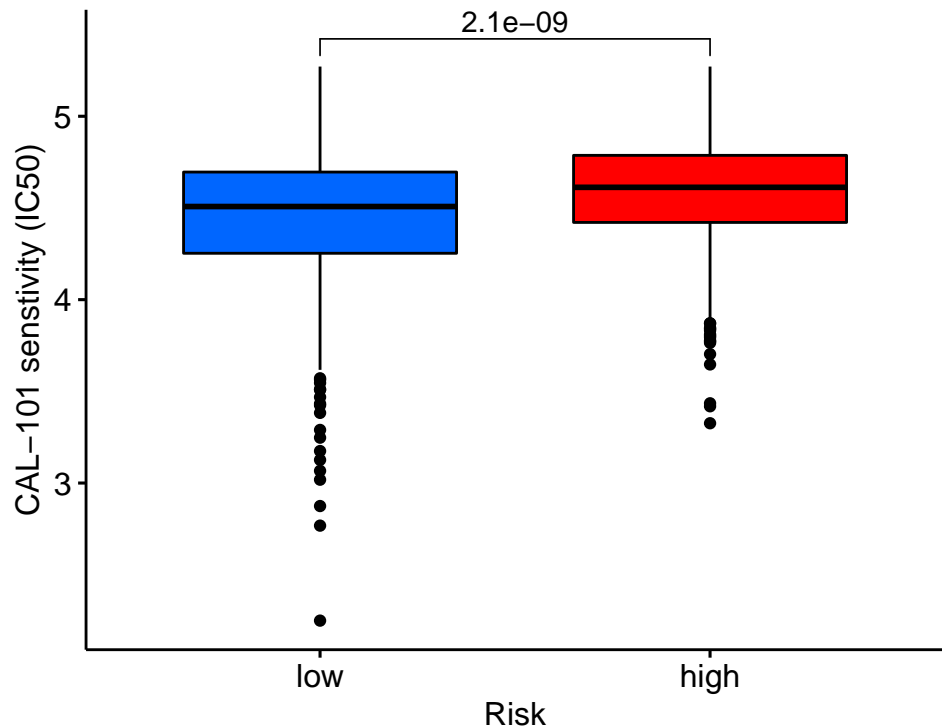

Supplement: Supplementary file 2 [file DataSheet_2.zip › durgSenstivity.CAL-101.pdf]

CGP-60474 sensitivity (IC50)

Risk 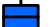 low 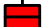 high

$1.5e-11$

low

high

Risk

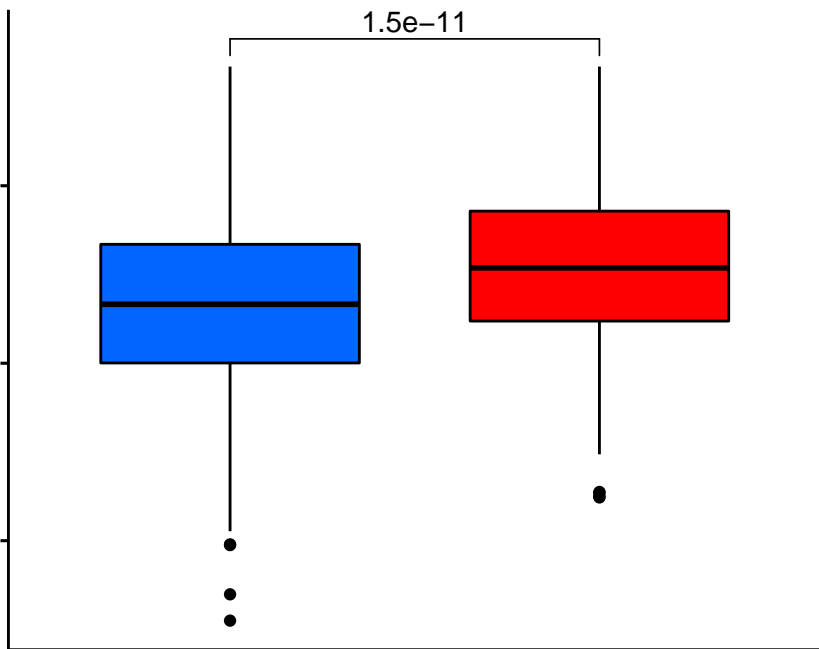

Supplement: Supplementary file 2 [file DataSheet_2.zip › durgSenstivity.CGP-60474.pdf]

Risk 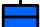 low 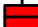 high

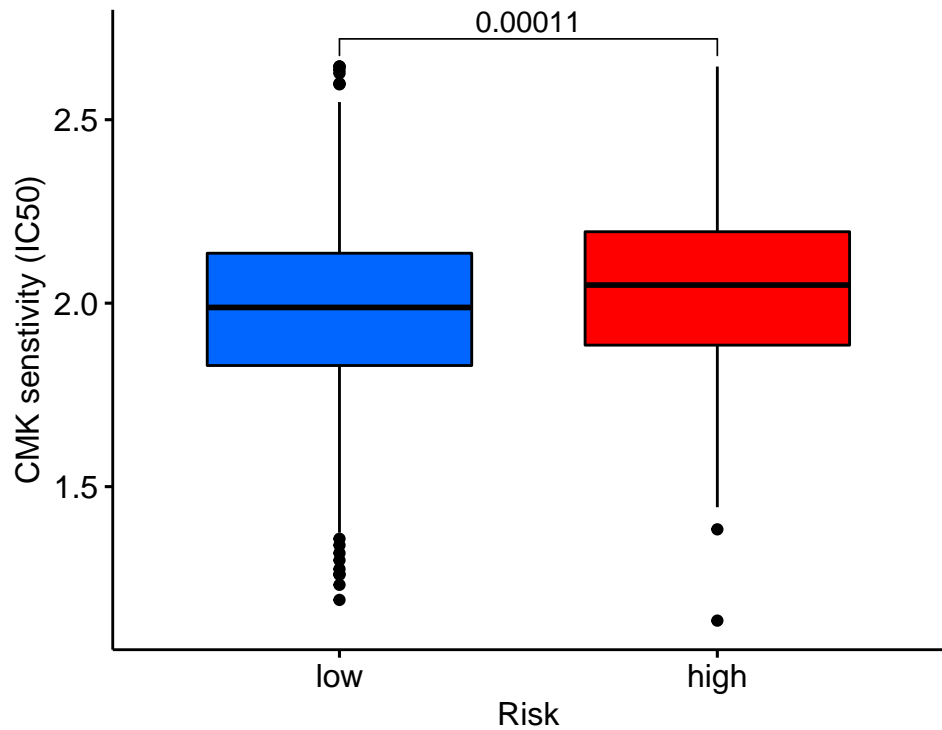

Supplement: Supplementary file 2 [file DataSheet_2.zip › durgSenstivity.CMK.pdf]

Risk 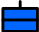 low 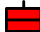 high

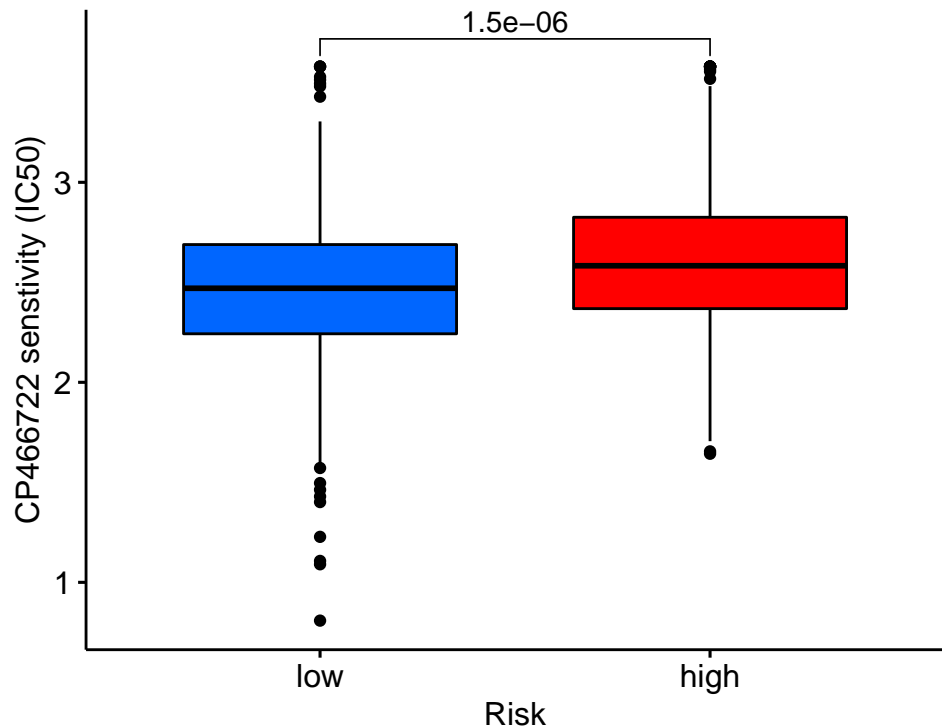

Supplement: Supplementary file 2 [file DataSheet_2.zip › durgSenstivity.CP466722.pdf]

Risk 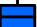 low 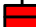 high

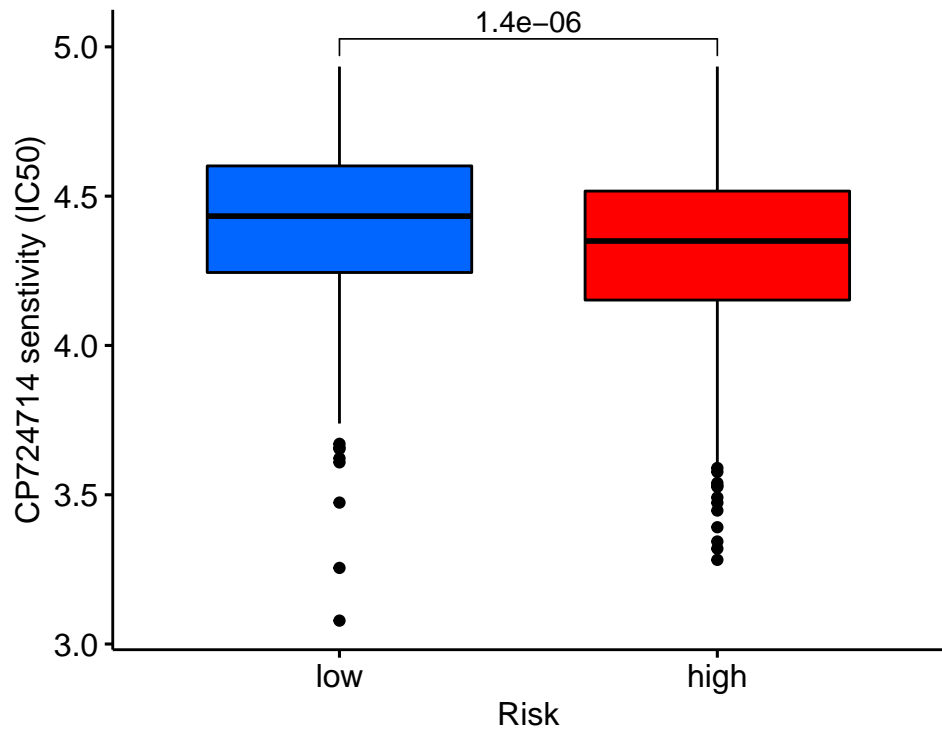

Supplement: Supplementary file 2 [file DataSheet_2.zip › durgSenstivity.CP724714.pdf]

Risk 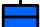 low 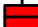 high

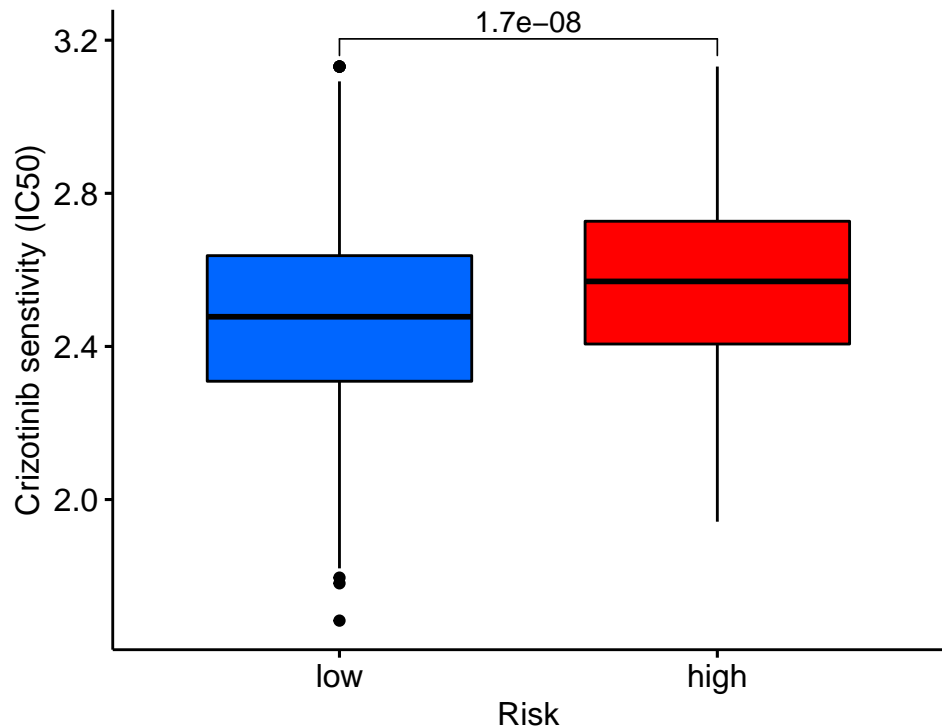

Supplement: Supplementary file 2 [file DataSheet_2.zip › durgSenstivity.Crizotinib.pdf]

Risk 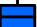 low 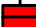 high

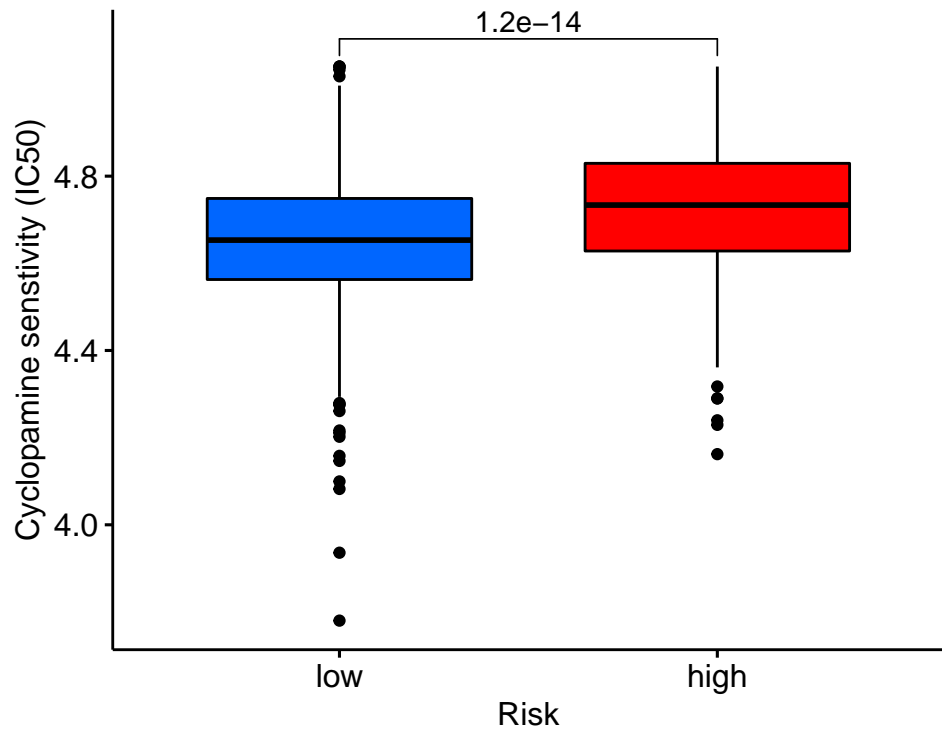

Supplement: Supplementary file 2 [file DataSheet_2.zip › durgSenstivity.Cyclopamine.pdf]

Risk 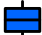 low 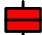 high

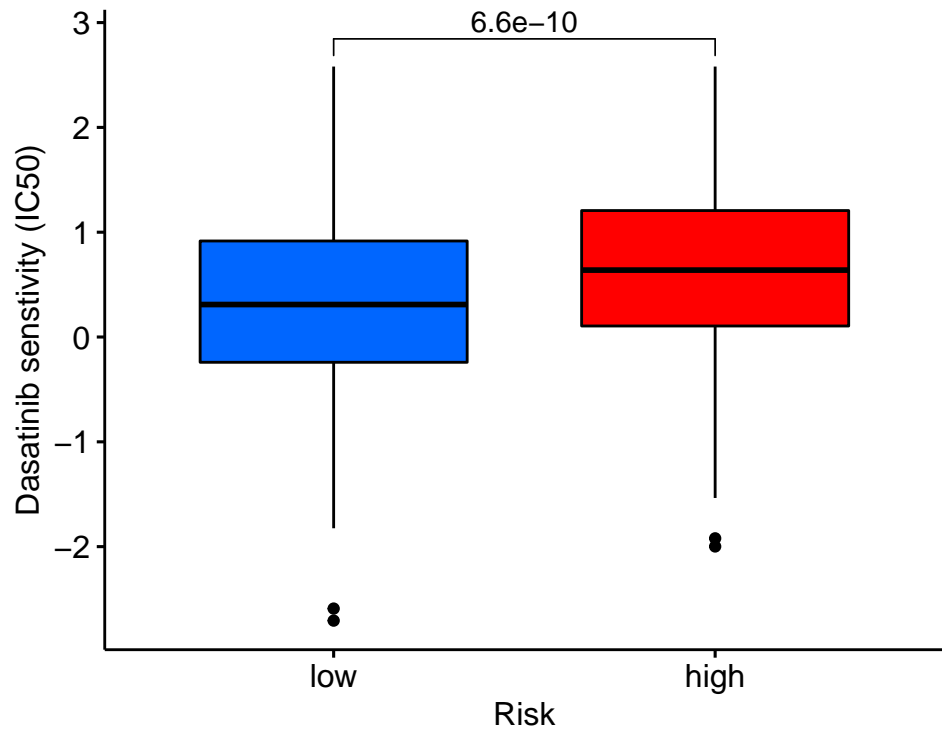

Supplement: Supplementary file 2 [file DataSheet_2.zip › durgSenstivity.Dasatinib.pdf]

Risk 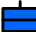 low 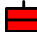 high

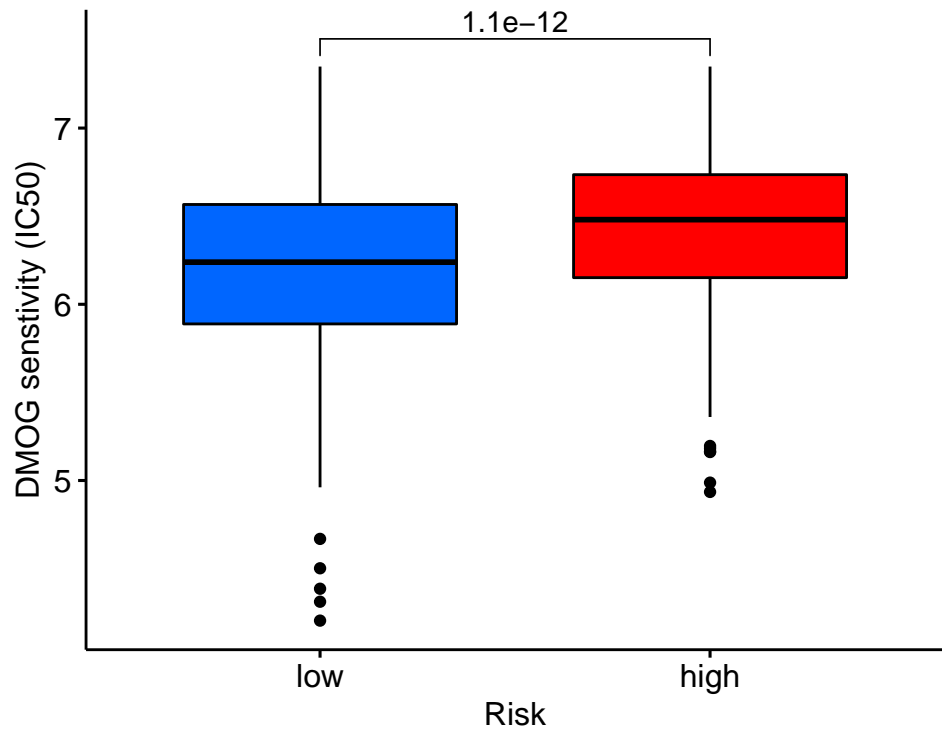

Supplement: Supplementary file 2 [file DataSheet_2.zip › durgSenstivity.DMOG.pdf]

Risk 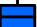 low 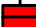 high

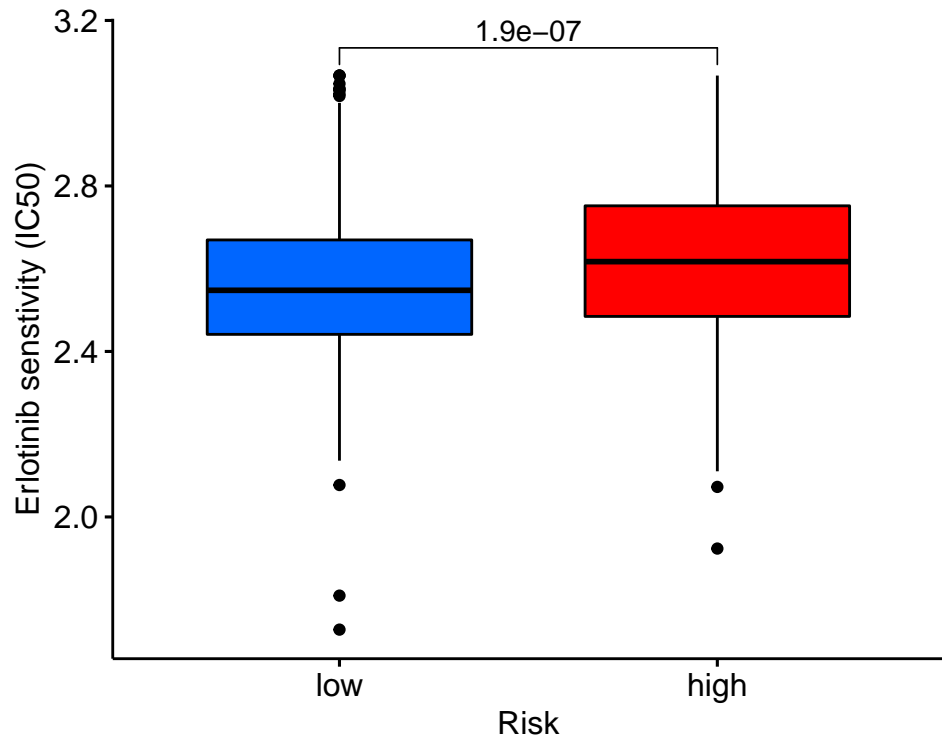

Supplement: Supplementary file 2 [file DataSheet_2.zip › durgSenstivity.Erlotinib.pdf]

Risk 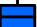 low 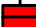 high

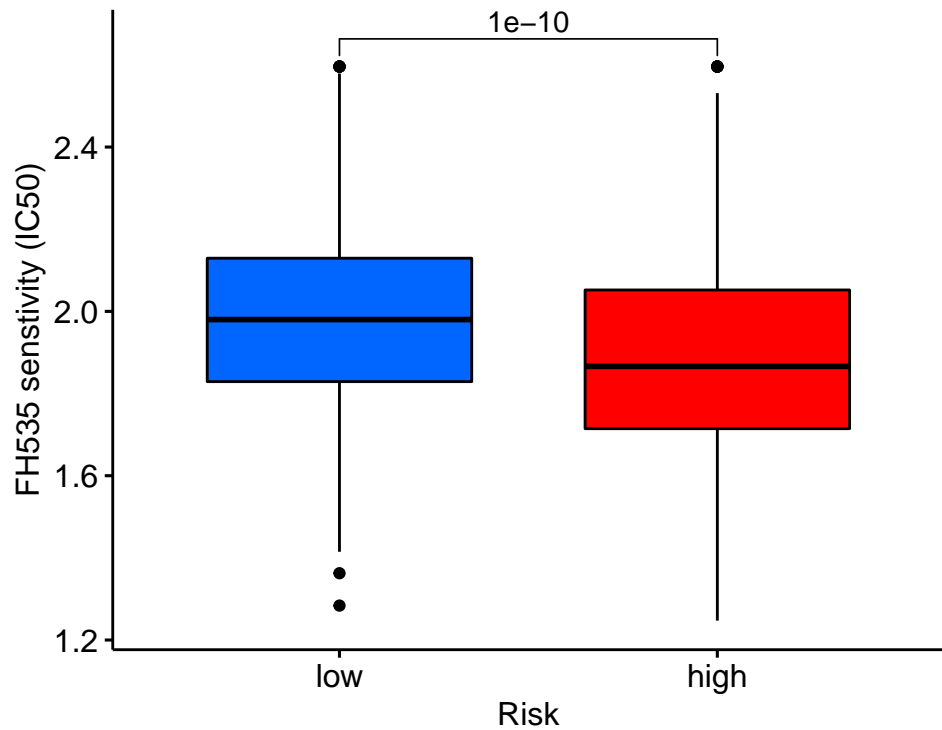

Supplement: Supplementary file 2 [file DataSheet_2.zip › durgSenstivity.FH535.pdf]

Risk 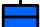 low 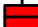 high

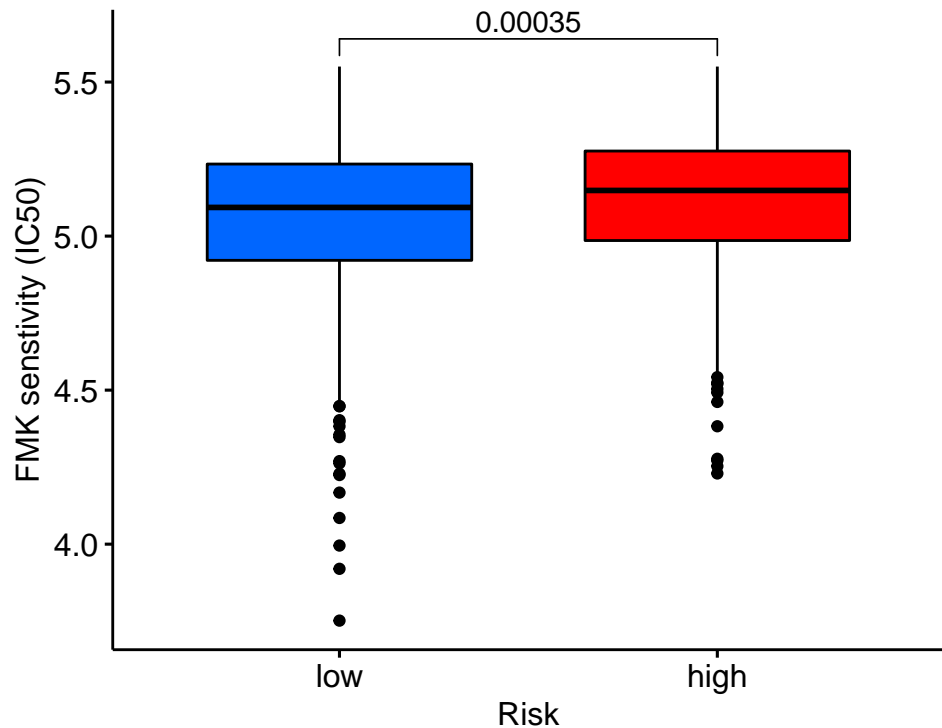

Supplement: Supplementary file 2 [file DataSheet_2.zip › durgSenstivity.FMK.pdf]

Risk 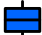 low 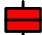 high

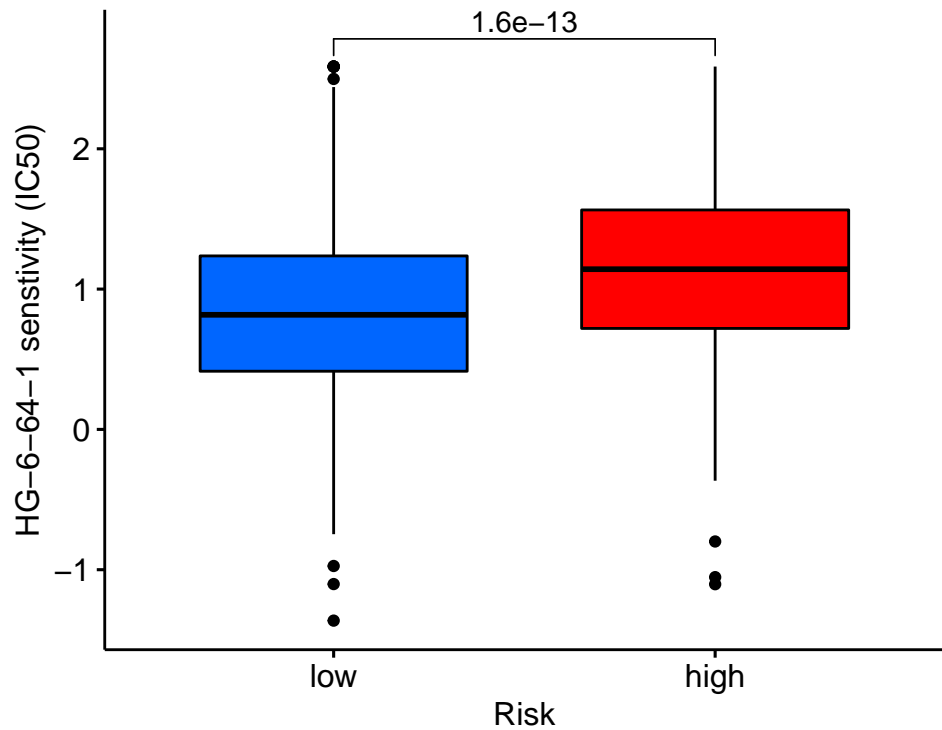

Supplement: Supplementary file 2 [file DataSheet_2.zip › durgSenstivity.HG-6-64-1.pdf]

Risk 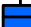 low 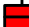 high

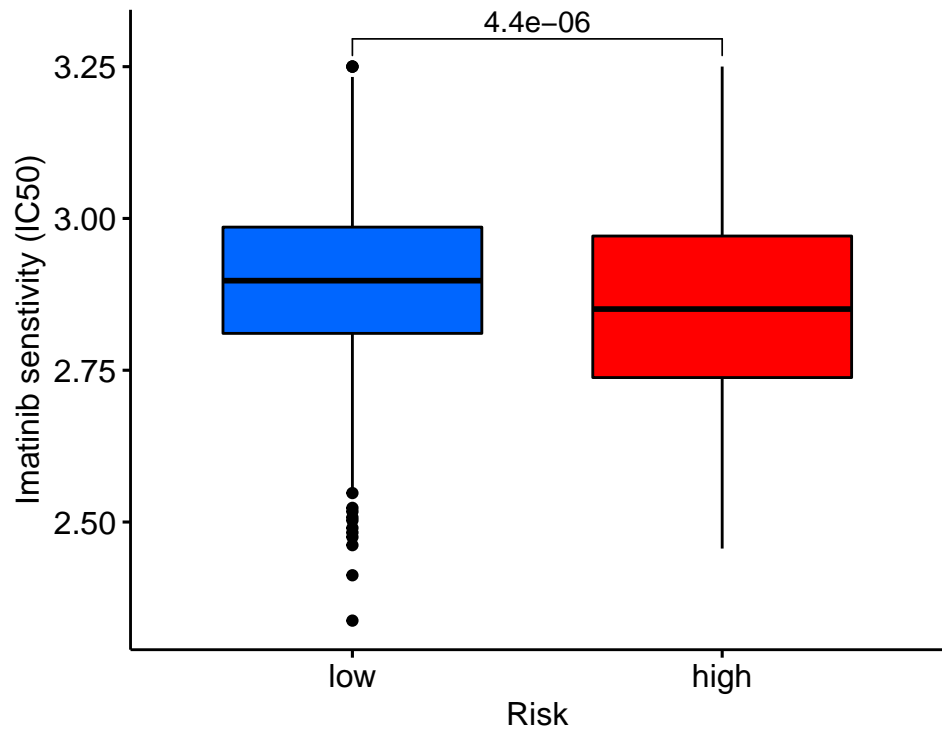

Supplement: Supplementary file 2 [file DataSheet_2.zip › durgSenstivity.Imatinib.pdf]

Risk 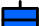 low 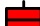 high

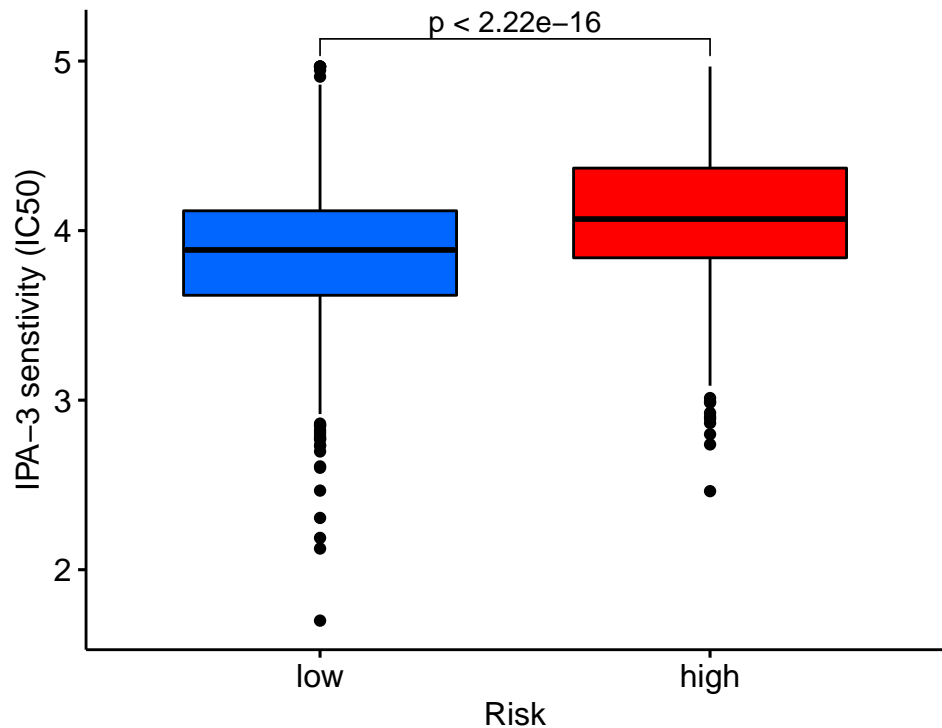

Supplement: Supplementary file 2 [file DataSheet_2.zip › durgSenstivity.IPA-3.pdf]

Risk 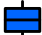 low 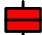 high

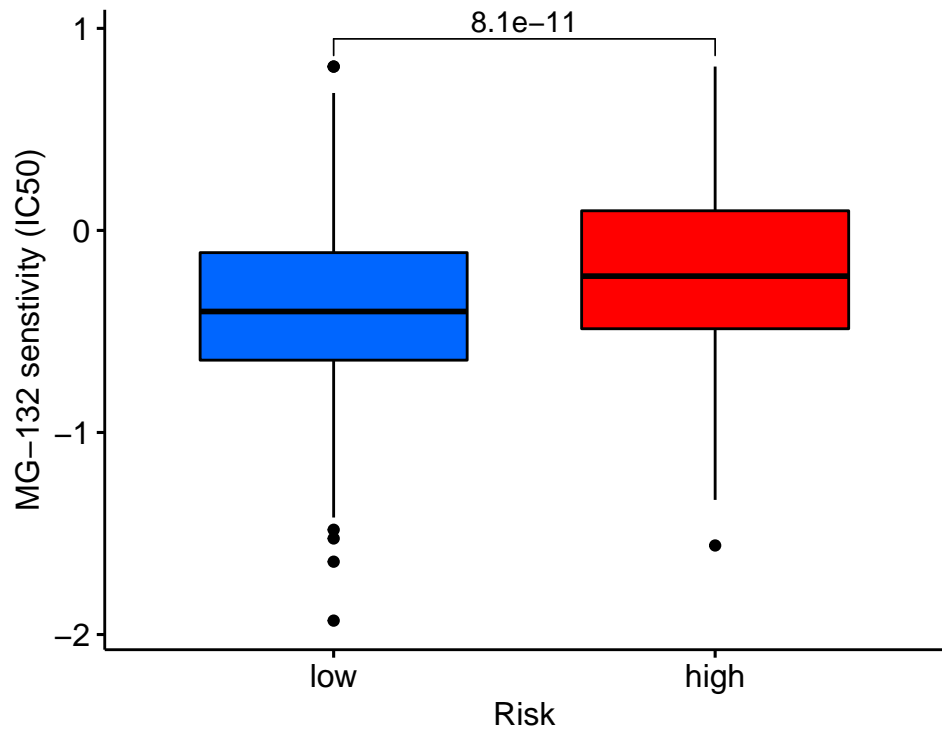

Supplement: Supplementary file 2 [file DataSheet_2.zip › durgSenstivity.MG-132.pdf]

Risk 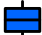 low 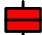 high

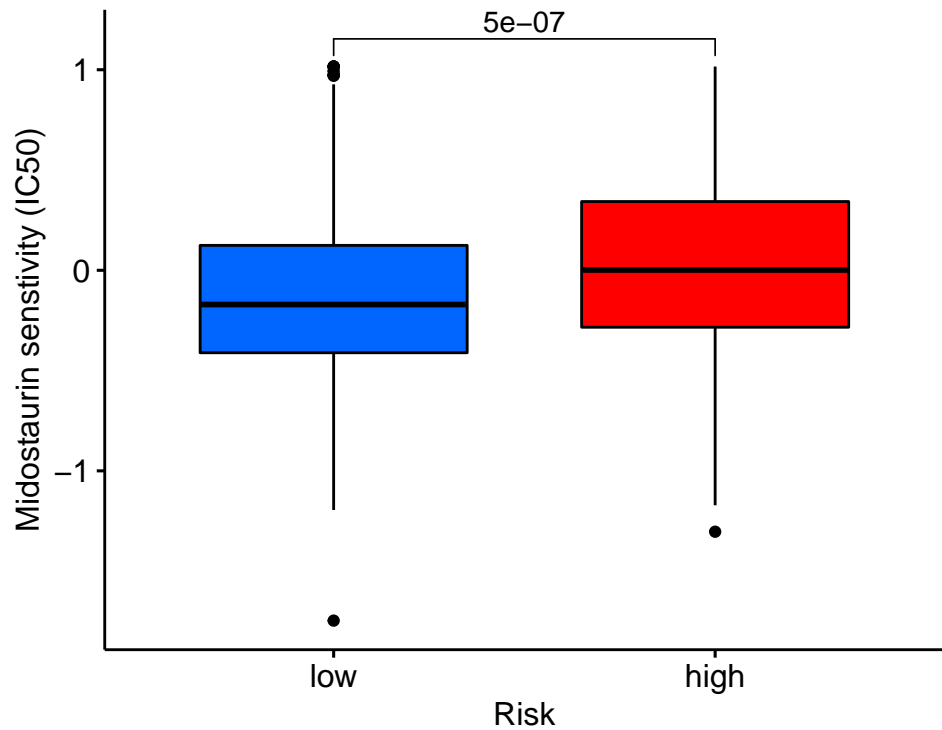

Supplement: Supplementary file 2 [file DataSheet_2.zip › durgSenstivity.Midostaurin.pdf]

Risk 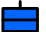 low 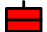 high

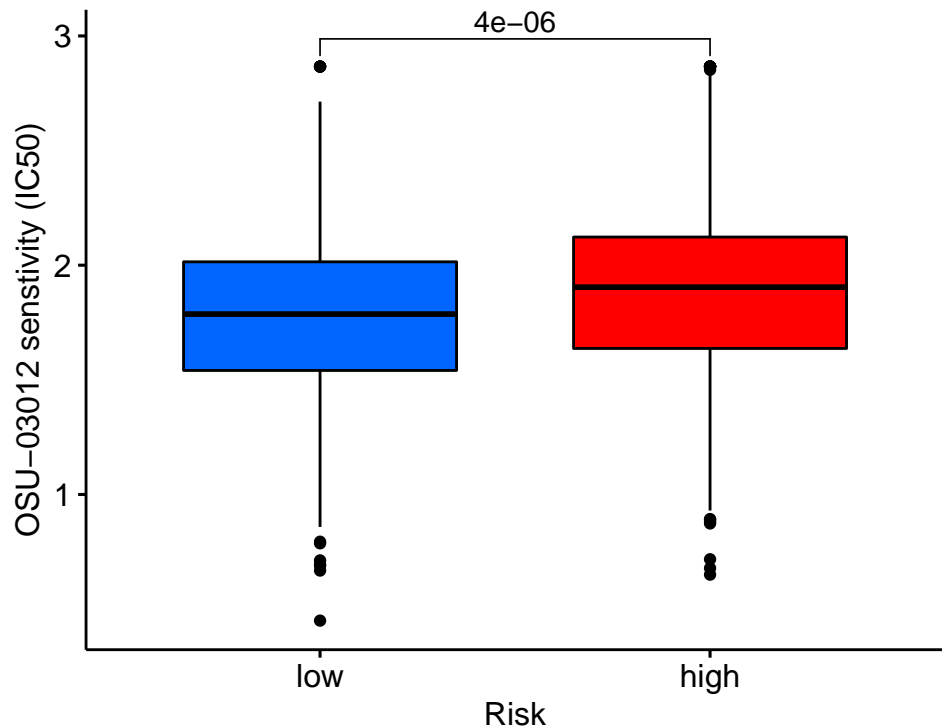

Supplement: Supplementary file 2 [file DataSheet_2.zip › durgSenstivity.OSU-03012.pdf]

Risk 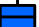 low 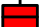 high

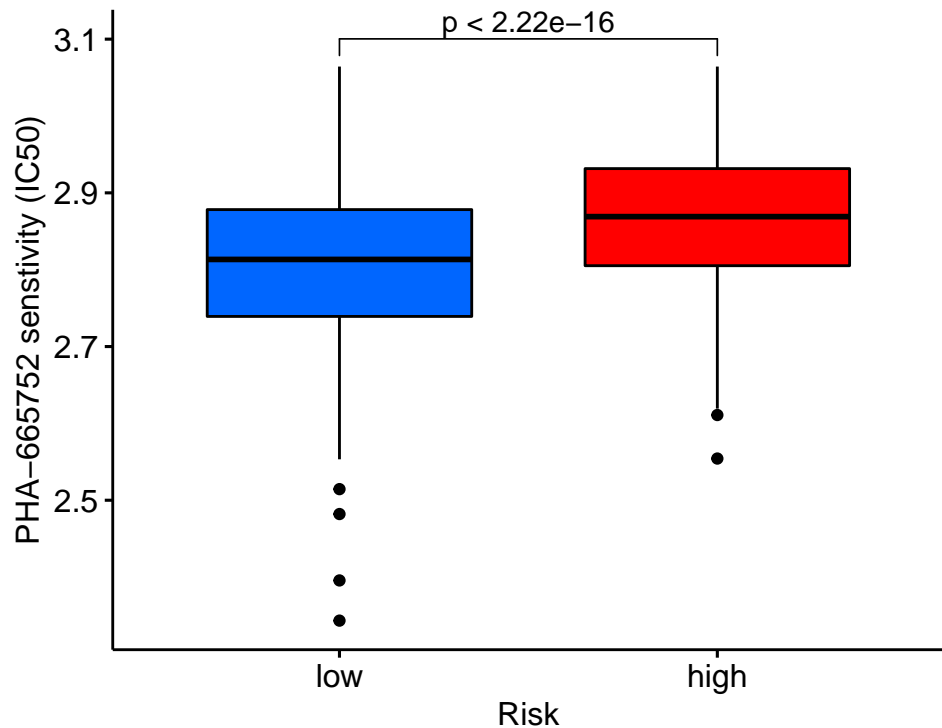

Supplement: Supplementary file 2 [file DataSheet_2.zip › durgSenstivity.PHA-665752.pdf]

Risk 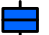 low 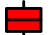 high

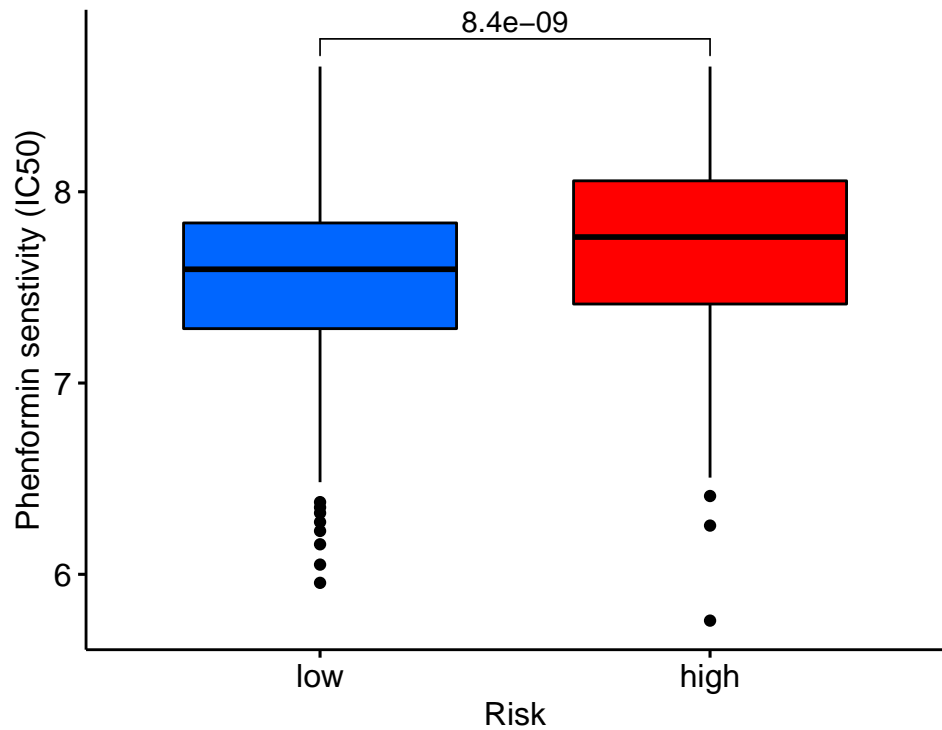

Supplement: Supplementary file 2 [file DataSheet_2.zip › durgSenstivity.Phenformin.pdf]

Risk 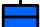 low 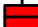 high

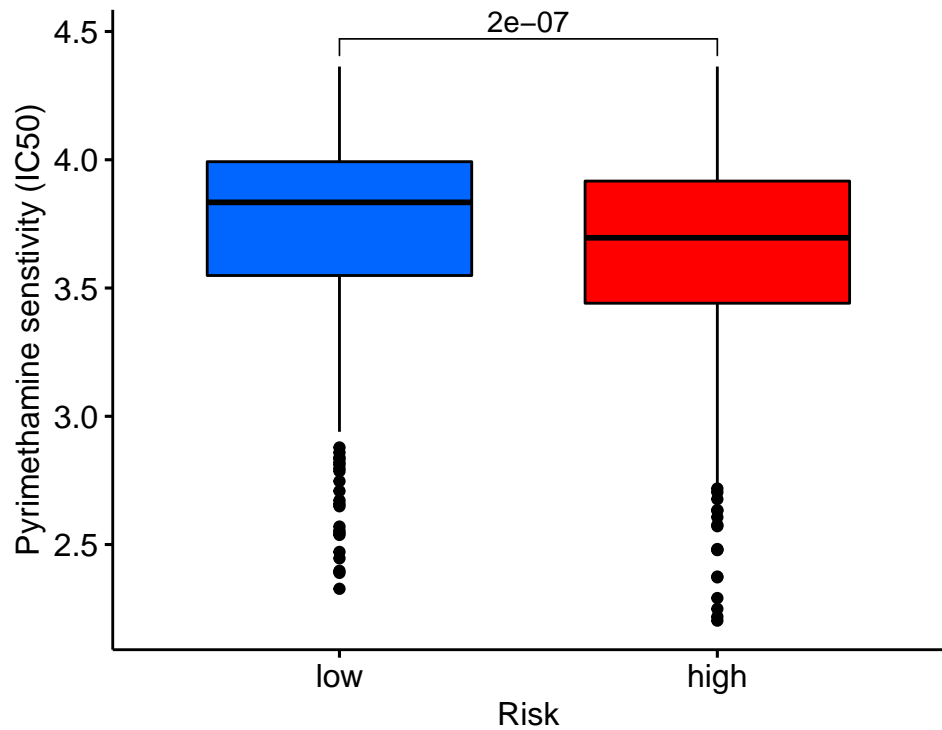

Supplement: Supplementary file 2 [file DataSheet_2.zip › durgSenstivity.Pyrimethamine.pdf]

Risk 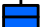 low 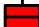 high

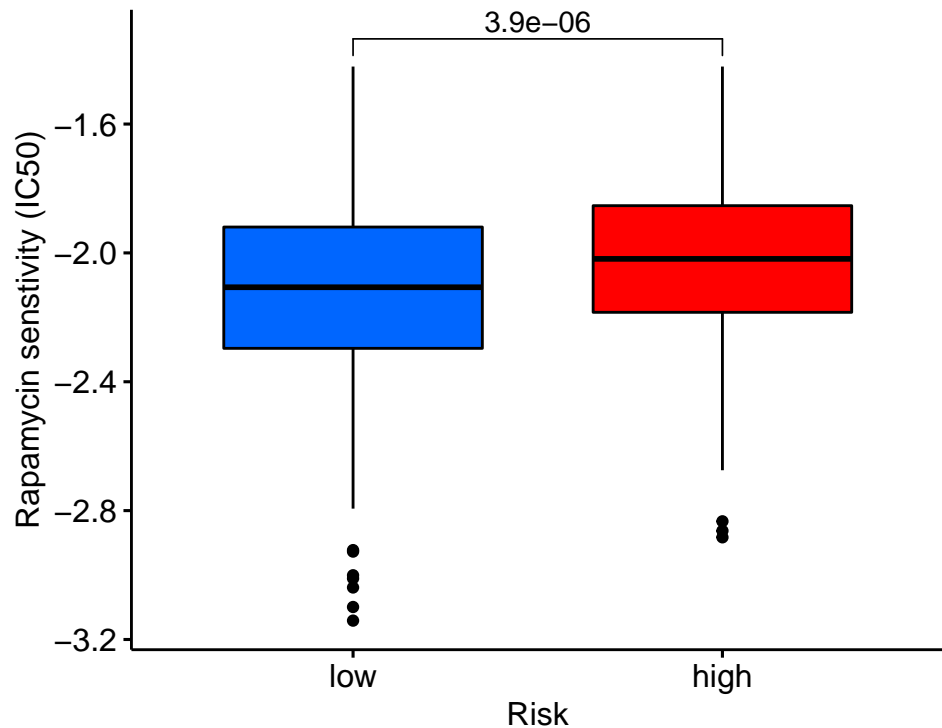

Supplement: Supplementary file 2 [file DataSheet_2.zip › durgSenstivity.Rapamycin.pdf]

Risk 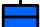 low 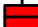 high

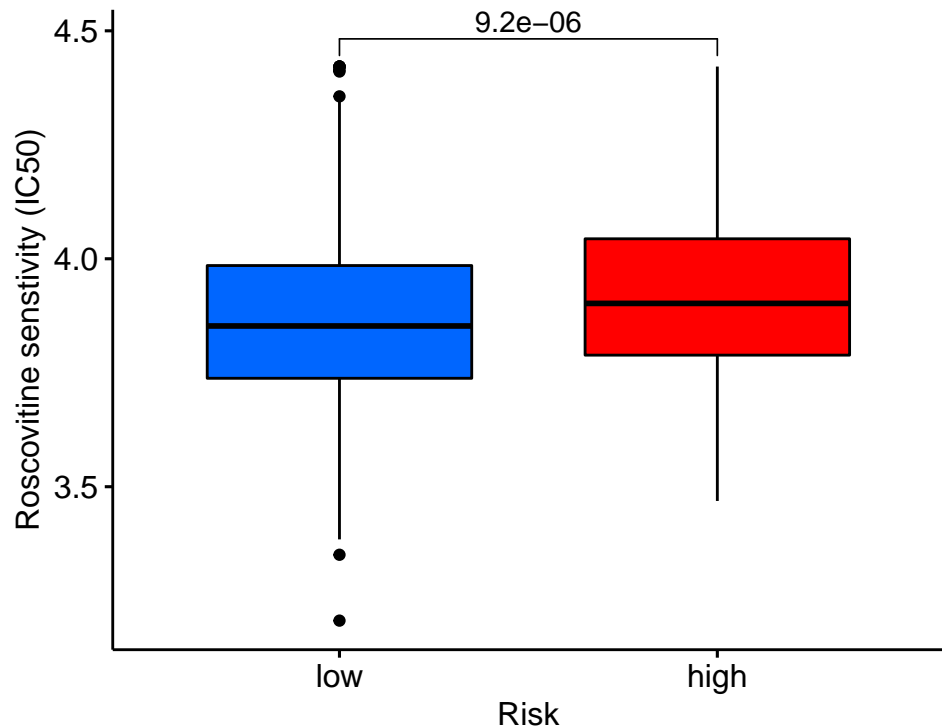

Supplement: Supplementary file 2 [file DataSheet_2.zip › durgSenstivity.Roscovitine.pdf]

Risk 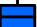 low 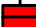 high

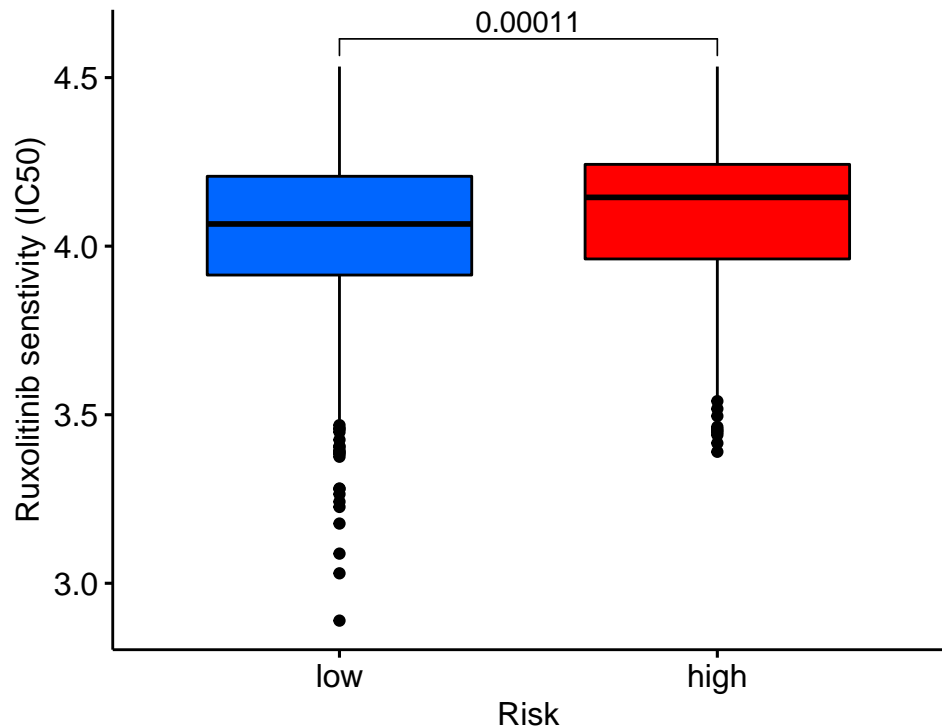

Supplement: Supplementary file 2 [file DataSheet_2.zip › durgSenstivity.Ruxolitinib.pdf]

Risk 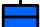 low 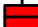 high

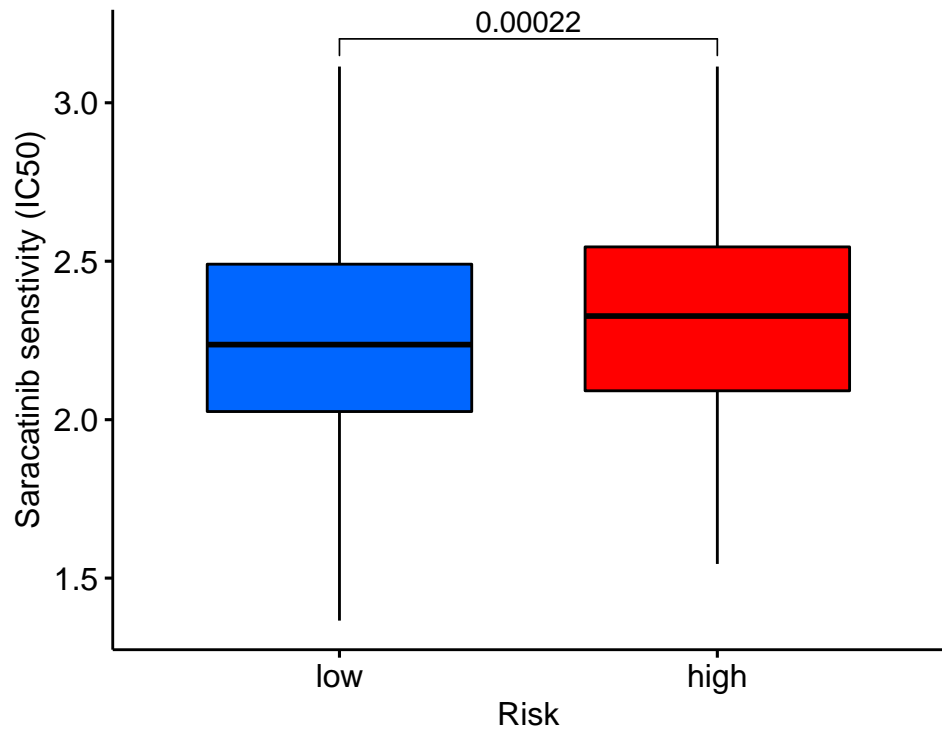

Supplement: Supplementary file 2 [file DataSheet_2.zip › durgSenstivity.Saracatinib.pdf]

Risk 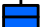 low 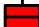 high

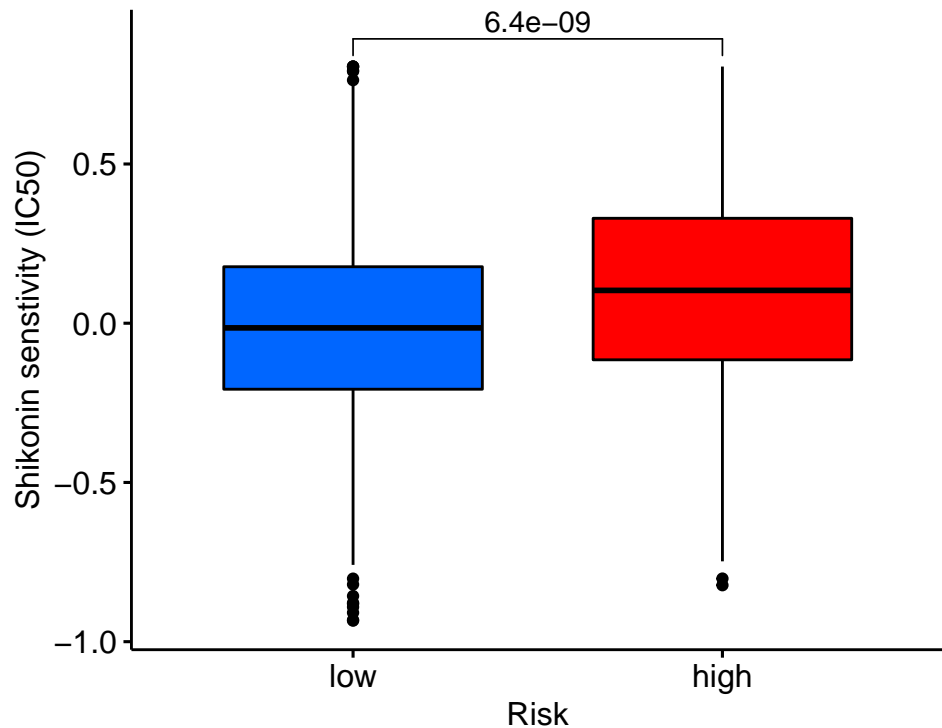

Supplement: Supplementary file 2 [file DataSheet_2.zip › durgSenstivity.Shikonin.pdf]

Risk 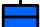 low 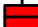 high

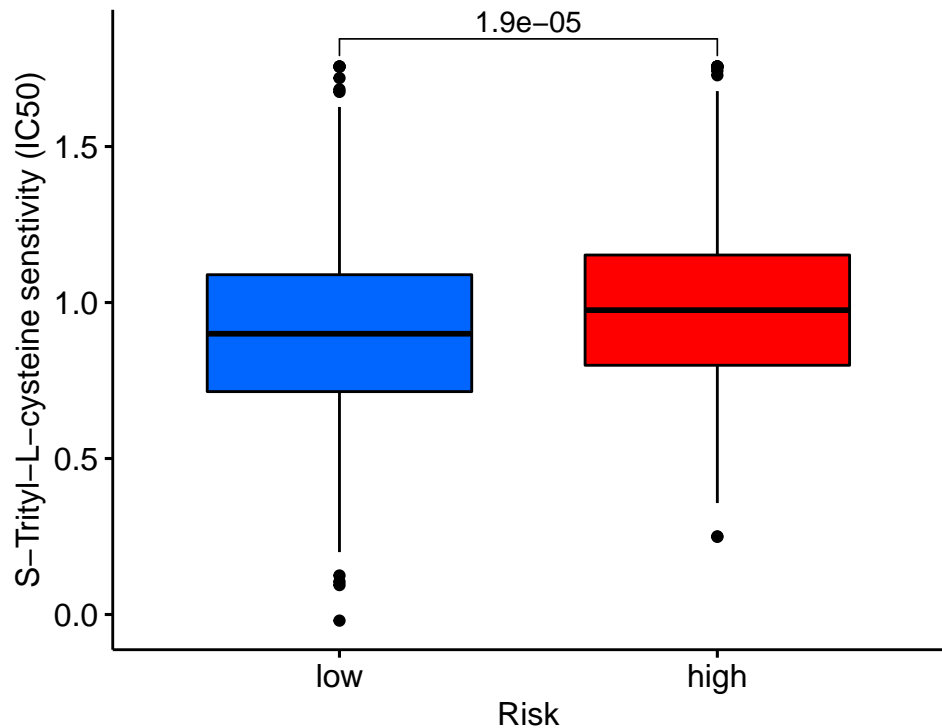

Supplement: Supplementary file 2 [file DataSheet_2.zip › durgSenstivity.S-Trityl-L-cysteine.pdf]

Risk 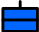 low 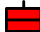 high

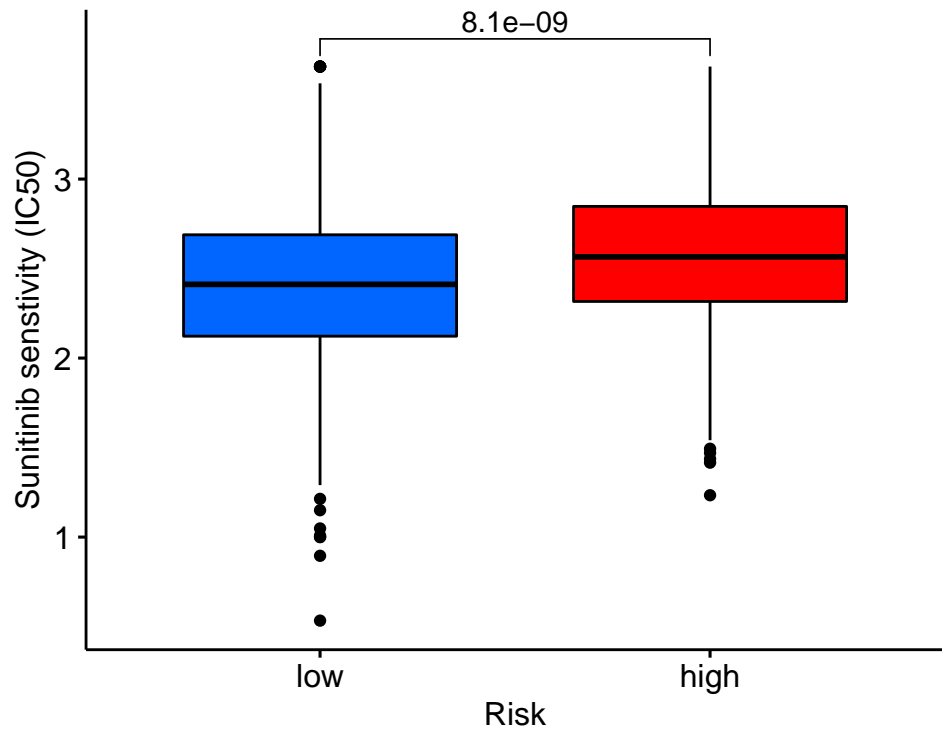

Supplement: Supplementary file 2 [file DataSheet_2.zip › durgSenstivity.Sunitinib.pdf]

Risk 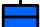 low 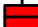 high

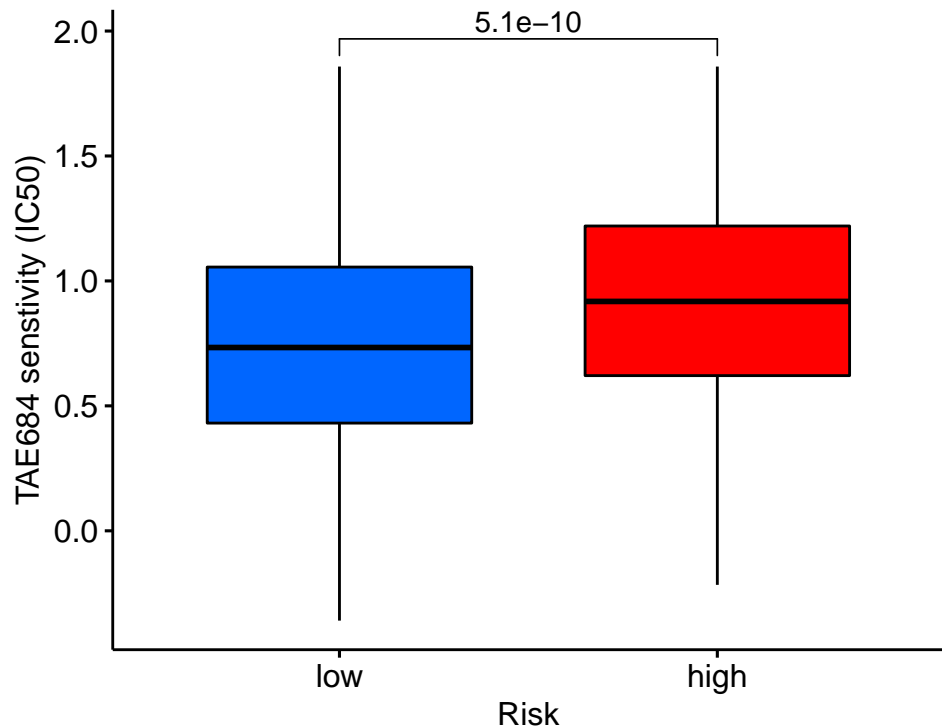

Supplement: Supplementary file 2 [file DataSheet_2.zip › durgSenstivity.TAE684.pdf]

Risk 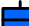 low 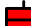 high

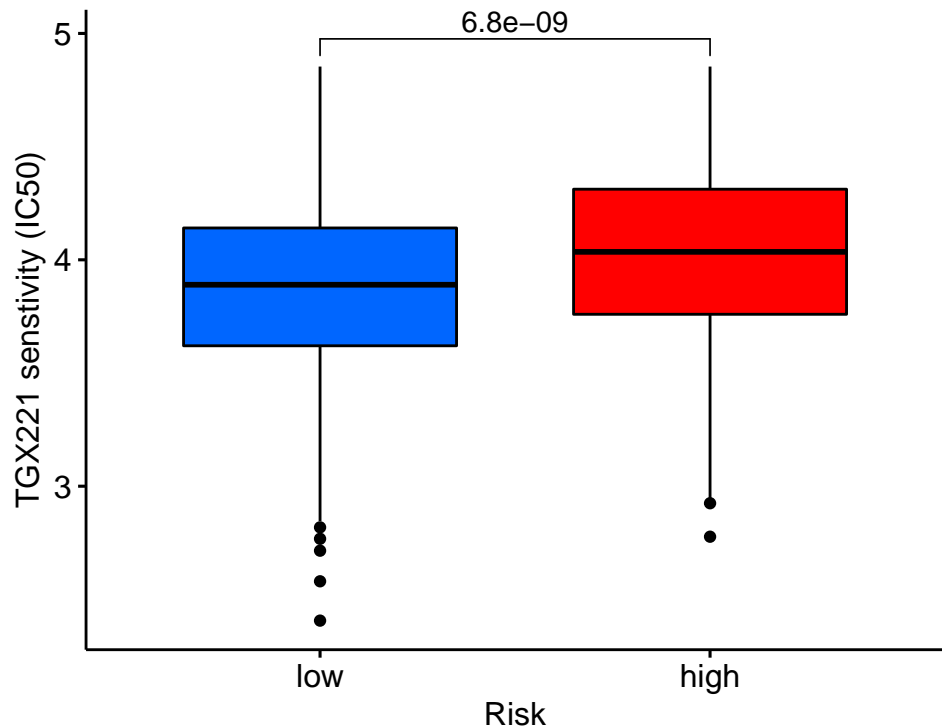

Supplement: Supplementary file 2 [file DataSheet_2.zip › durgSenstivity.TGX221.pdf]

Risk 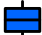 low 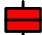 high

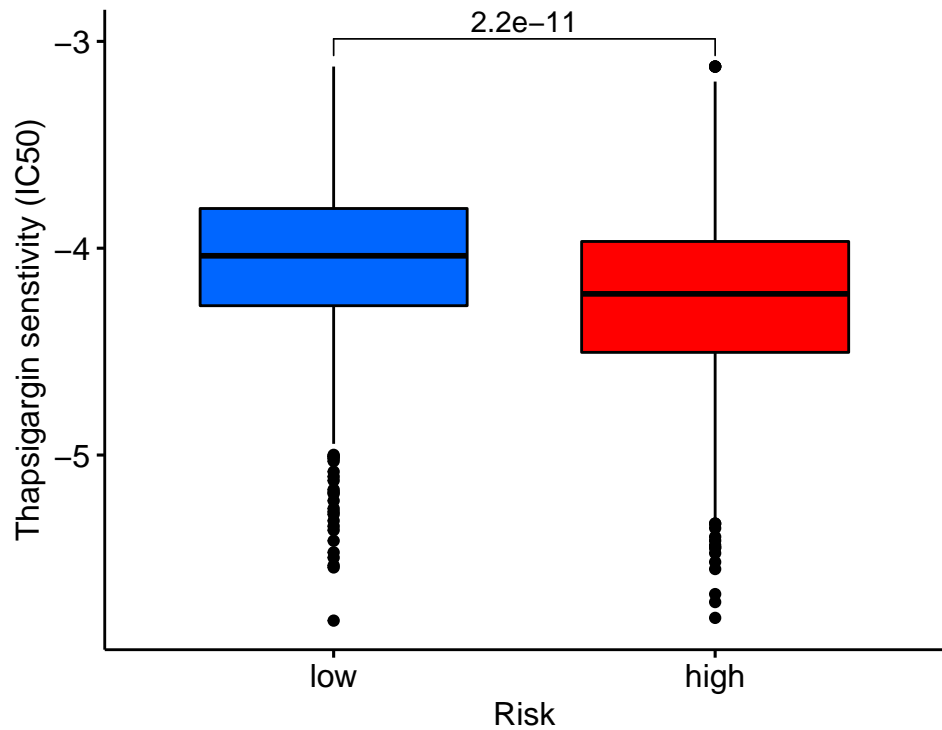

Supplement: Supplementary file 2 [file DataSheet_2.zip › durgSenstivity.Thapsigargin.pdf]

Risk 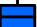 low 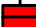 high

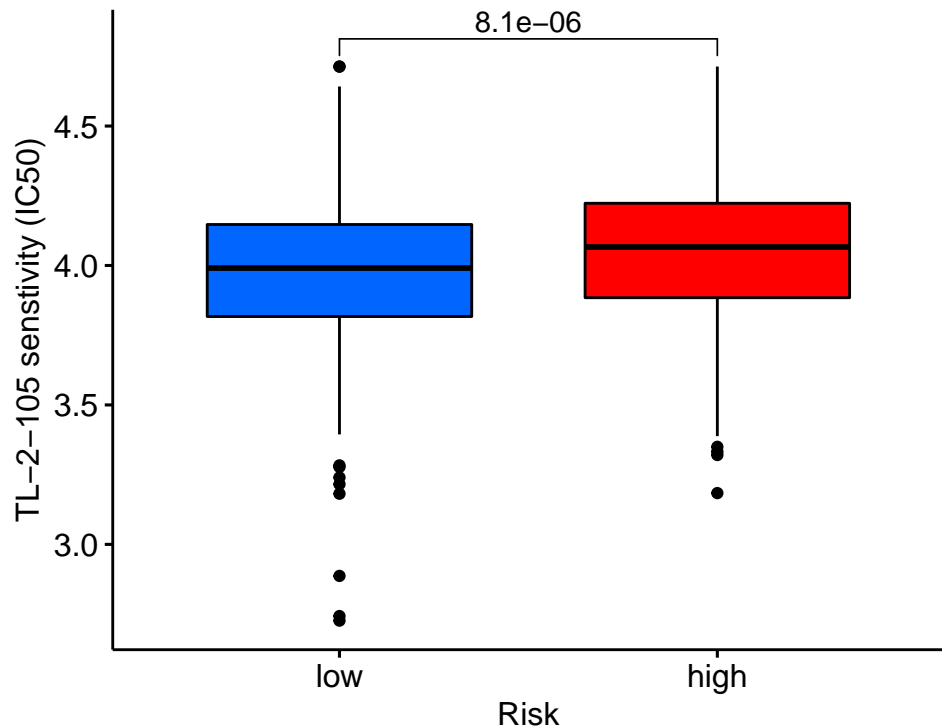

Supplement: Supplementary file 2 [file DataSheet_2.zip › durgSenstivity.TL-2-105.pdf]

Risk 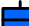 low 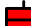 high

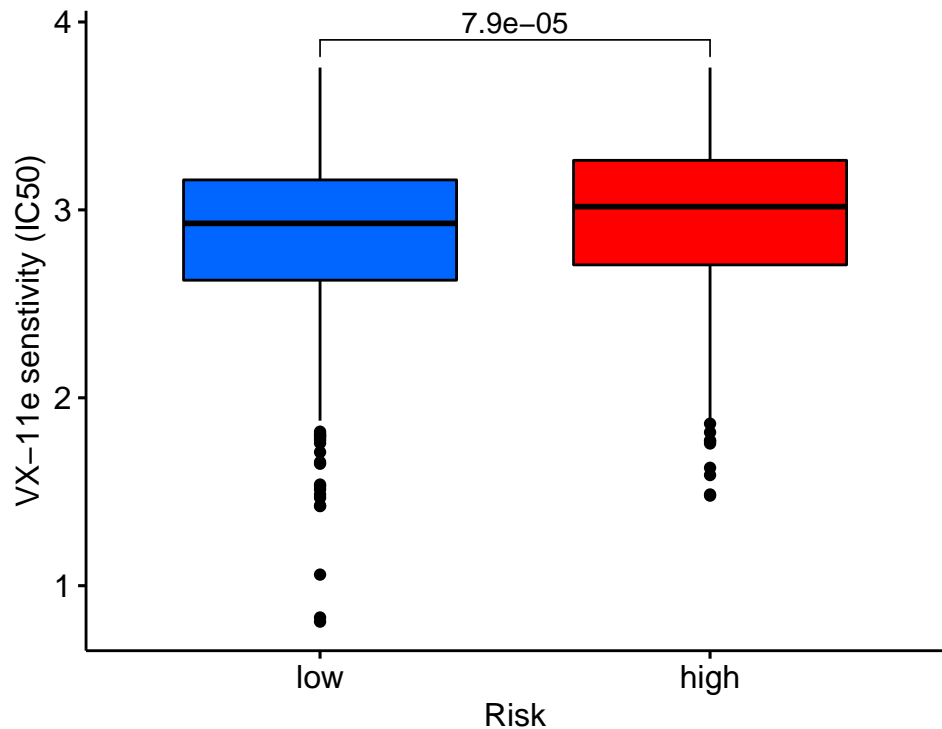

Supplement: Supplementary file 2 [file DataSheet_2.zip › durgSenstivity.VX-11e.pdf]

Risk 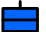 low 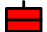 high

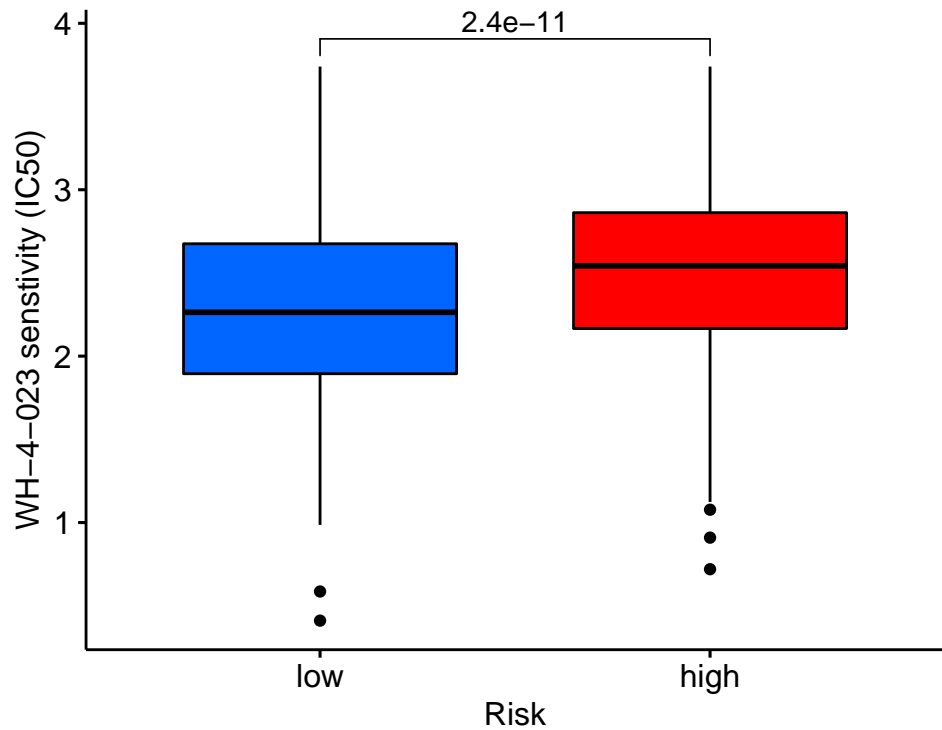

Supplement: Supplementary file 2 [file DataSheet_2.zip › durgSenstivity.WH-4-023.pdf]

Risk 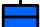 low 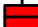 high

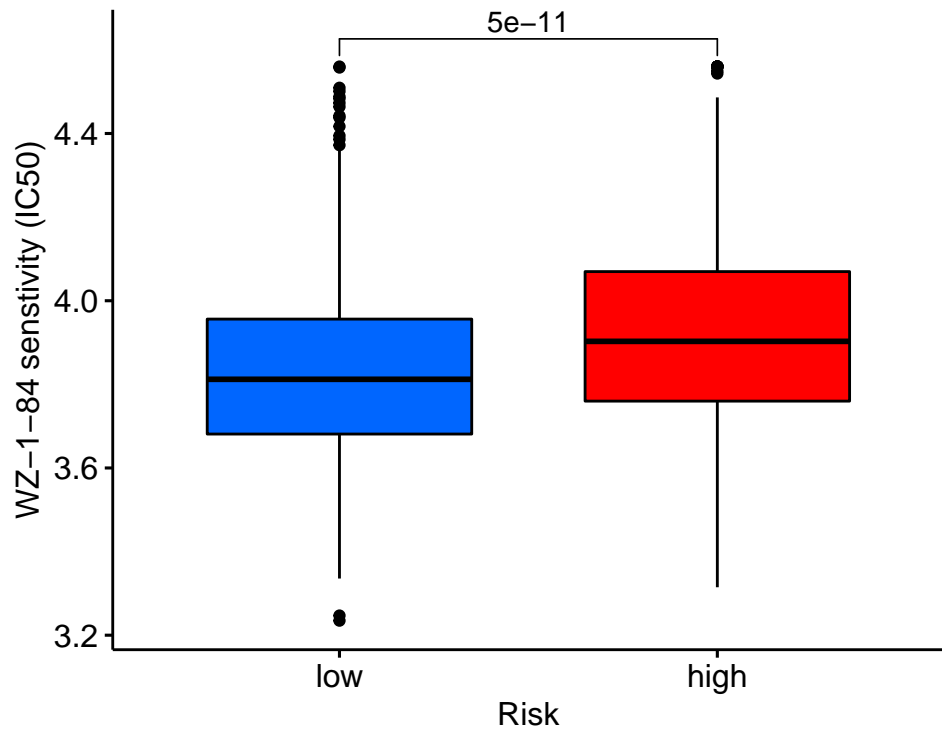

Supplement: Supplementary file 2 [file DataSheet_2.zip › durgSenstivity.WZ-1-84.pdf]

Risk 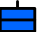 low 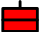 high

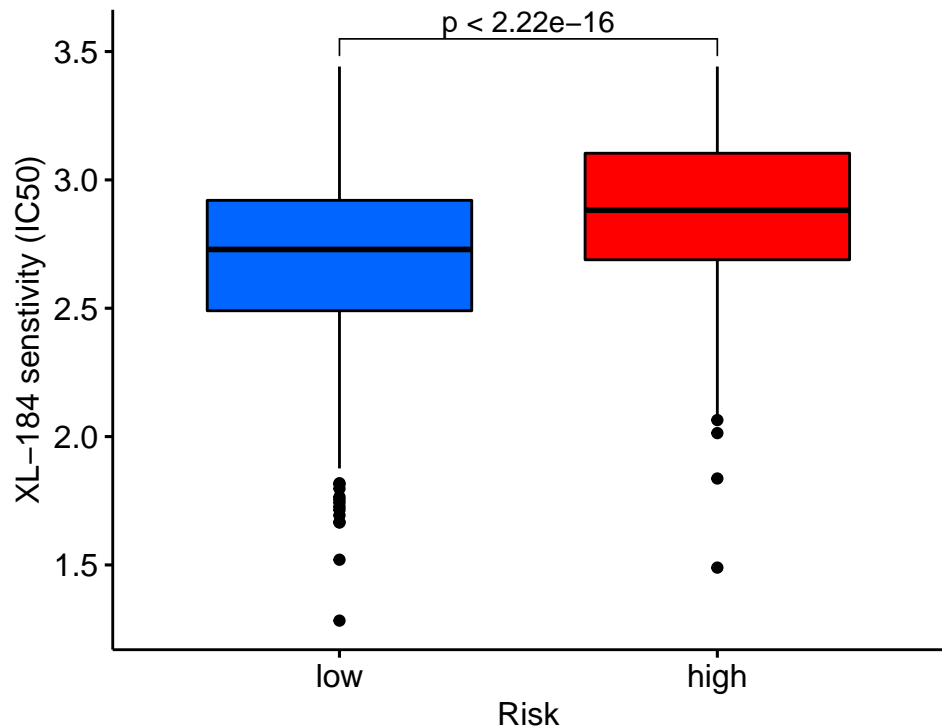

Supplement: Supplementary file 2 [file DataSheet_2.zip › durgSenstivity.XL-184.pdf]

Risk 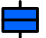 low 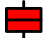 high

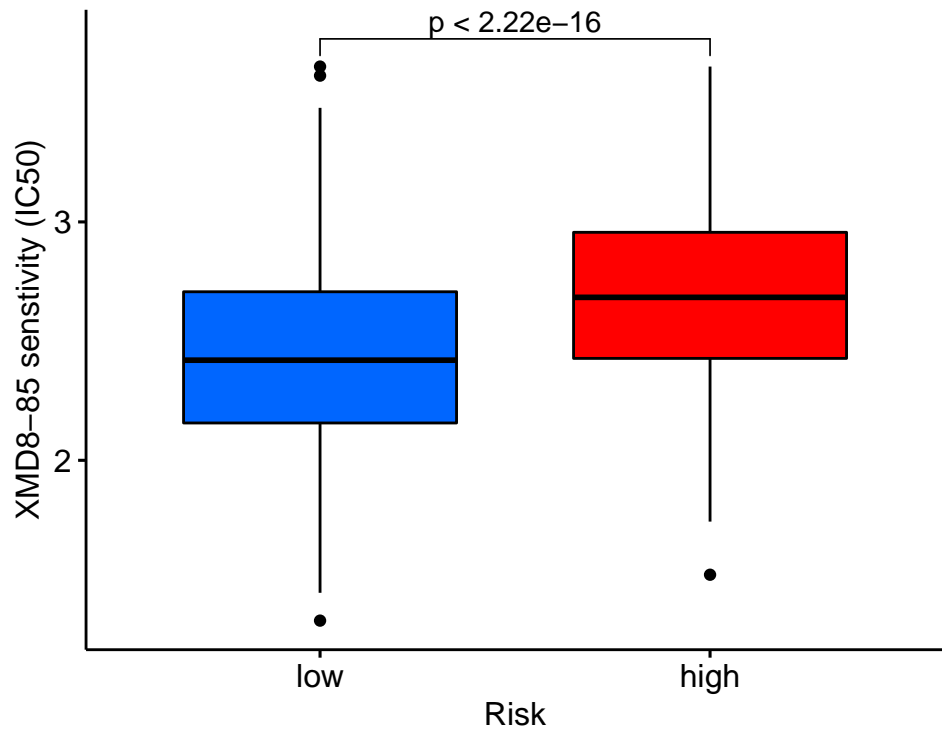

Supplement: Supplementary file 2 [file DataSheet_2.zip › durgSenstivity.XMD8-85.pdf]

Risk 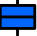 low 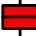 high

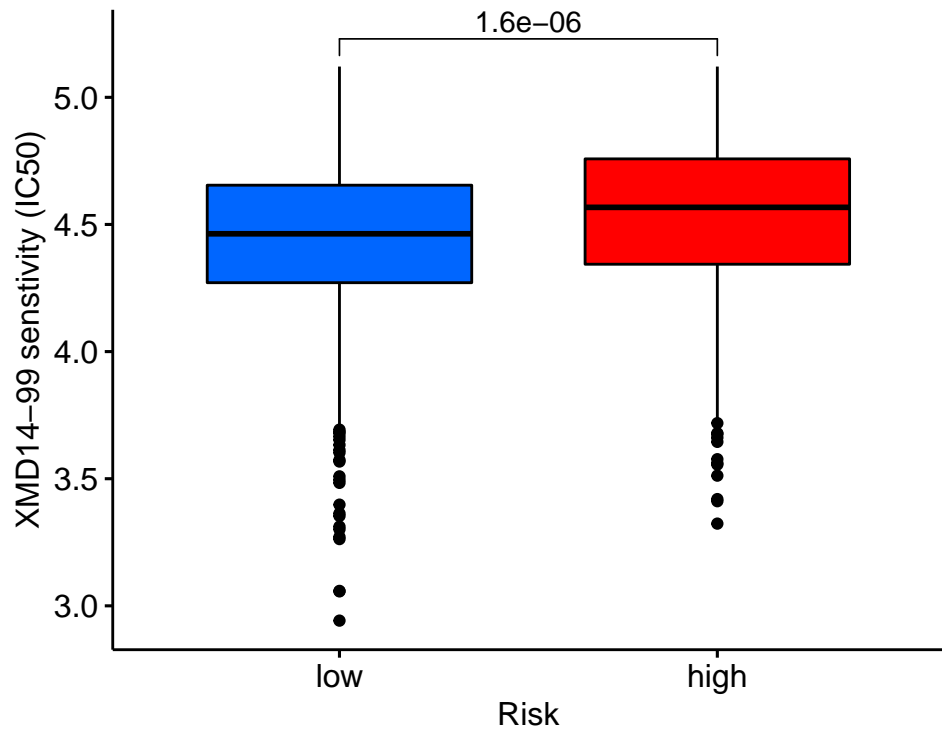

Supplement: Supplementary file 2 [file DataSheet_2.zip › durgSenstivity.XMD14-99.pdf]

Risk 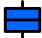 low 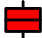 high

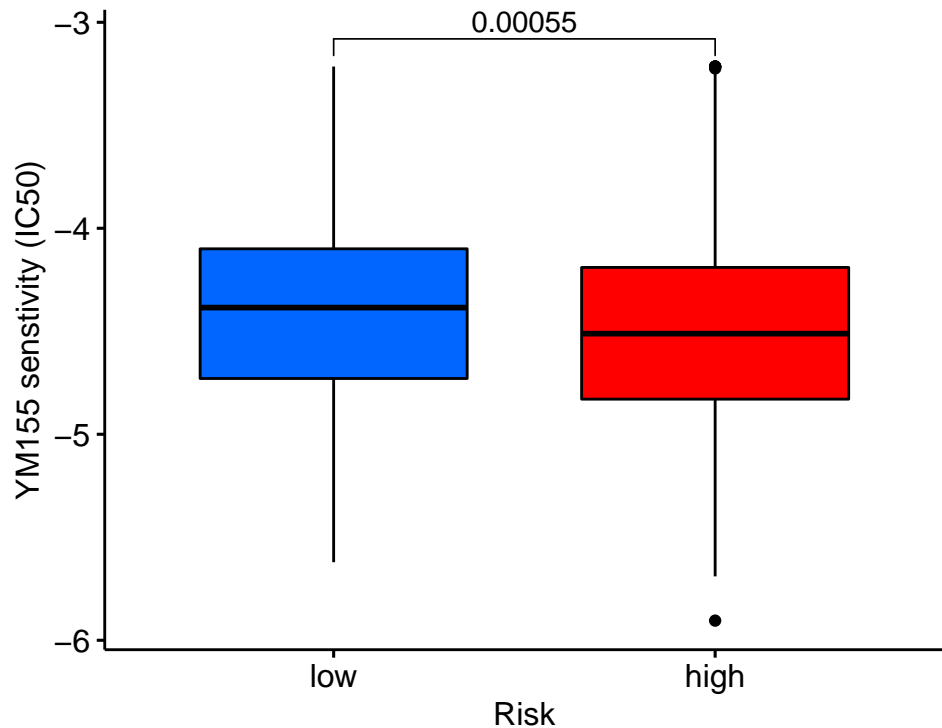

Supplement: Supplementary file 2 [file DataSheet_2.zip › durgSenstivity.YM155.pdf]

Risk 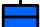 low 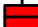 high

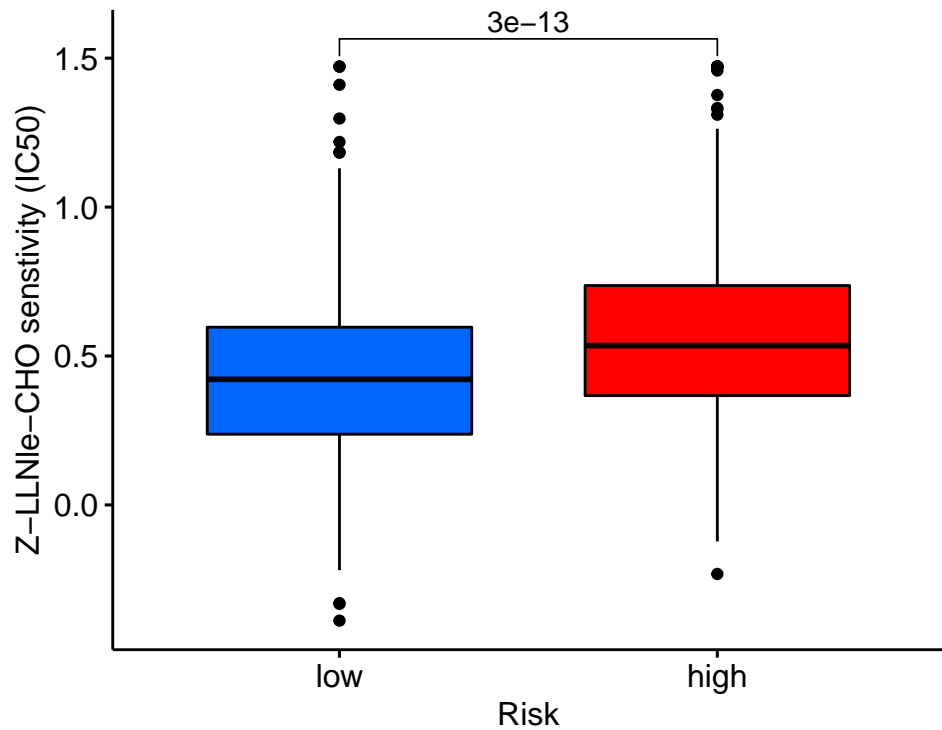

Supplement: Supplementary file 2 [file DataSheet_2.zip › durgSenstivity.Z-LLNle-CHO.pdf]

Risk 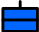 low 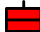 high

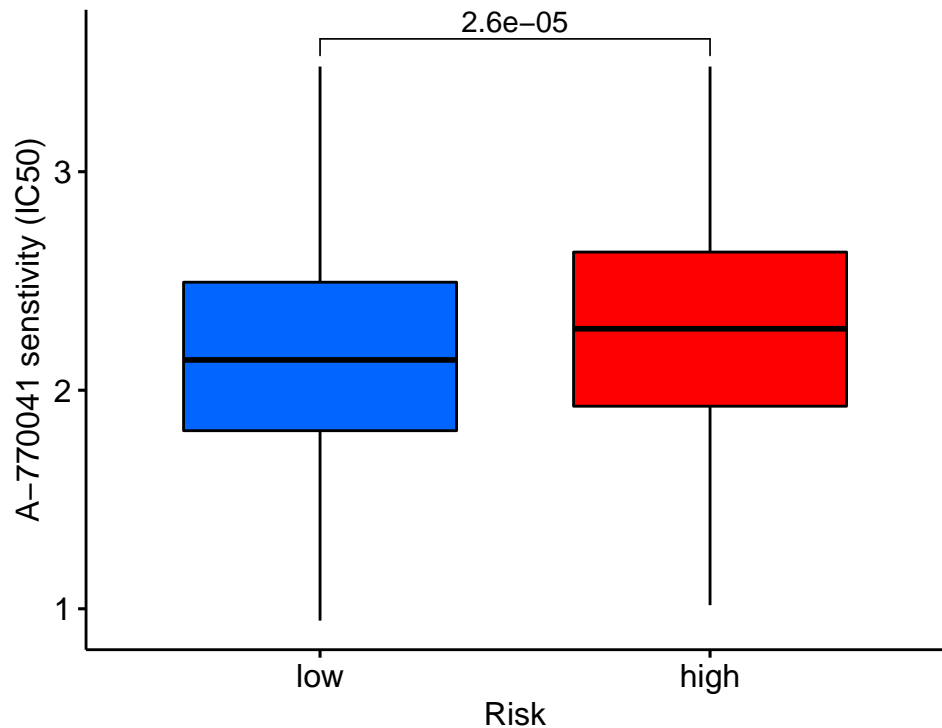

Supplement: Supplementary file 2 [file DataSheet_2.zip › durgSenstivity.A-770041.pdf]

Risk 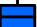 low 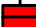 high

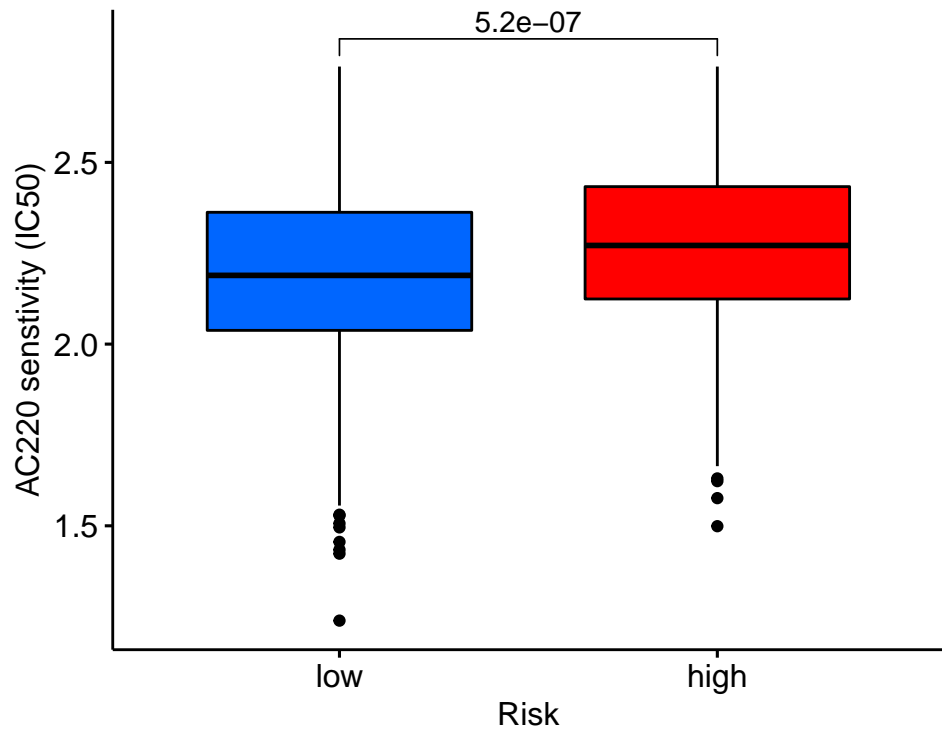

Supplement: Supplementary file 2 [file DataSheet_2.zip › durgSenstivity.AC220.pdf]

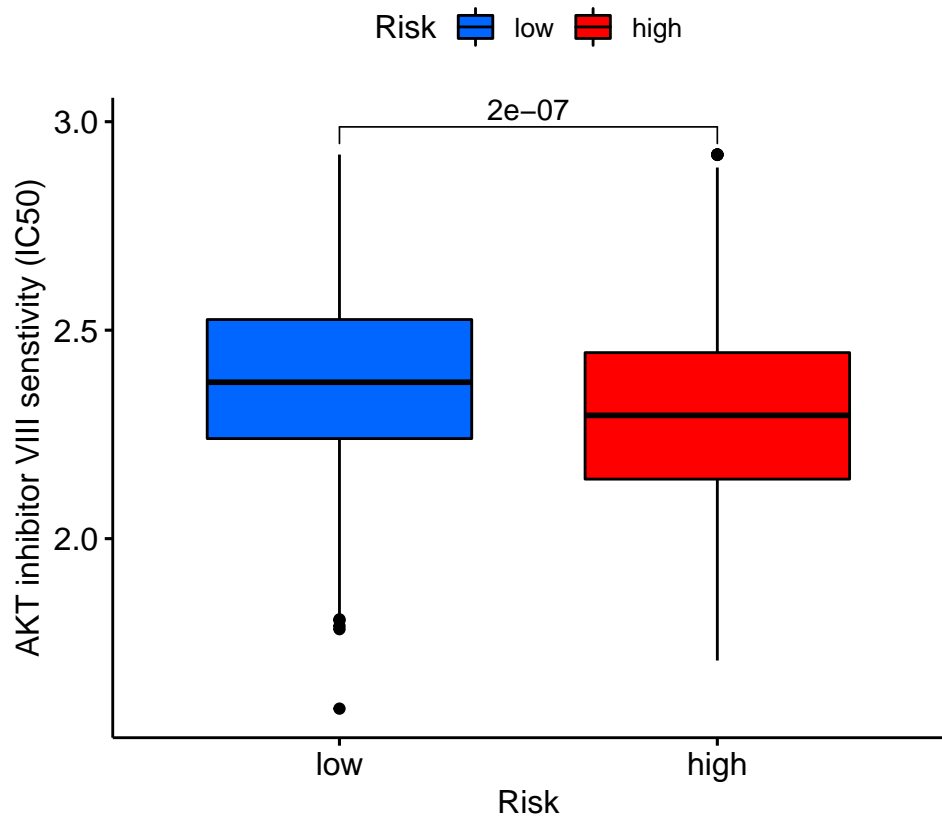

Supplement: Supplementary file 2 [file DataSheet_2.zip › durgSenstivity.AKT inhibitor VIII.pdf]

Risk 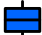 low 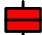 high

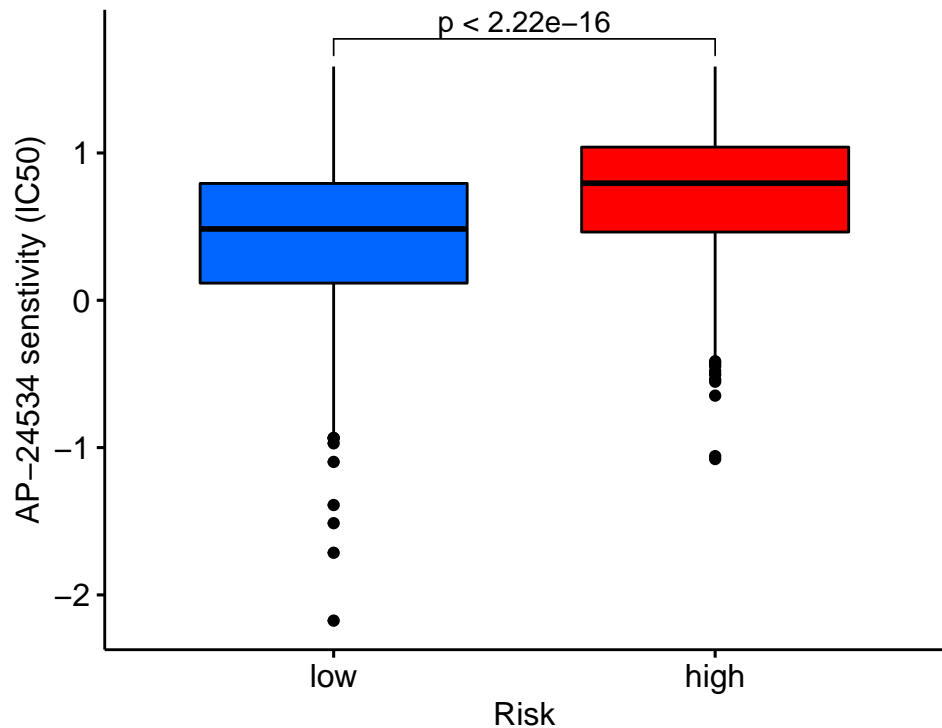

Supplement: Supplementary file 2 [file DataSheet_2.zip › durgSenstivity.AP-24534.pdf]

Risk 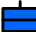 low 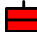 high

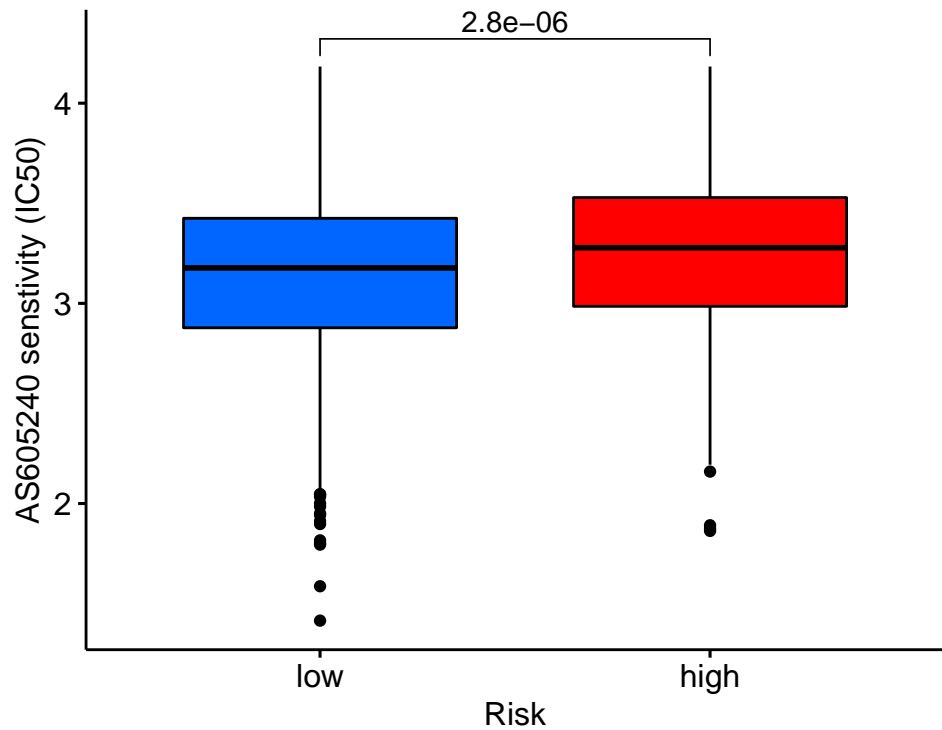

Supplement: Supplementary file 2 [file DataSheet_2.zip › durgSenstivity.AS605240.pdf]

Risk 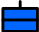 low 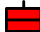 high

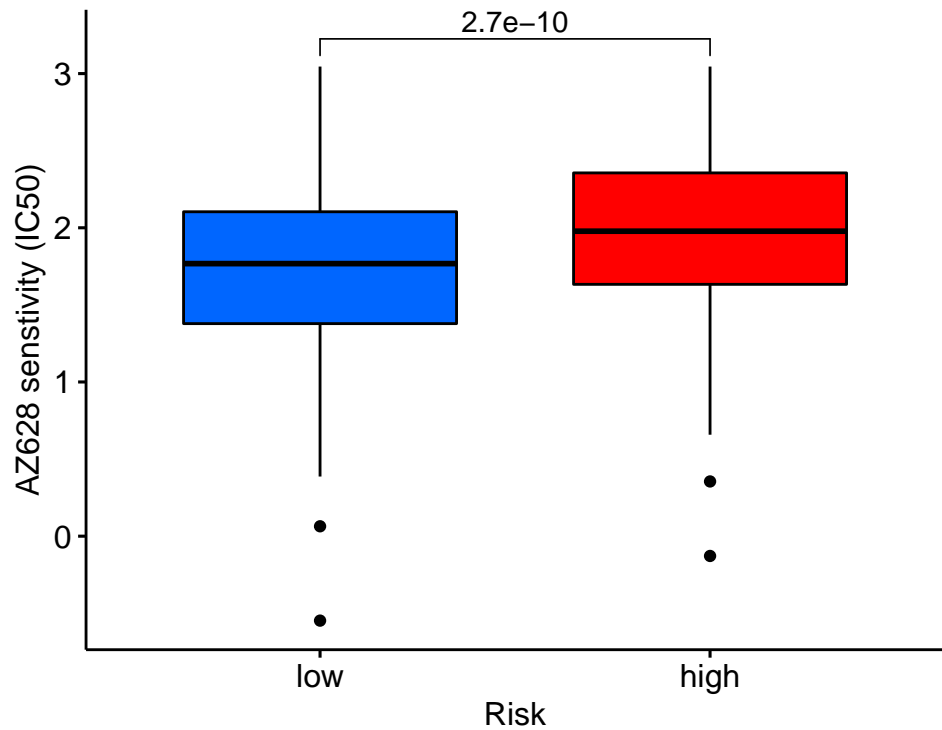

Supplement: Supplementary file 2 [file DataSheet_2.zip › durgSenstivity.AZ628.pdf]

Risk 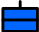 low 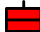 high

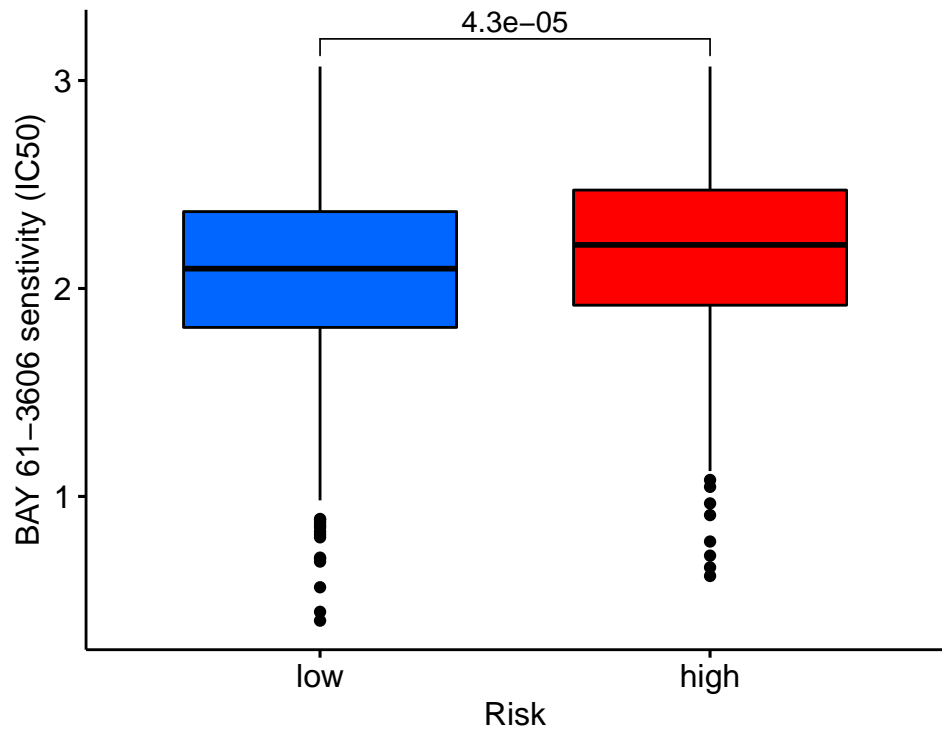

Supplement: Supplementary file 2 [file DataSheet_2.zip › durgSenstivity.BAY 61-3606.pdf]

Risk 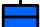 low 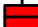 high

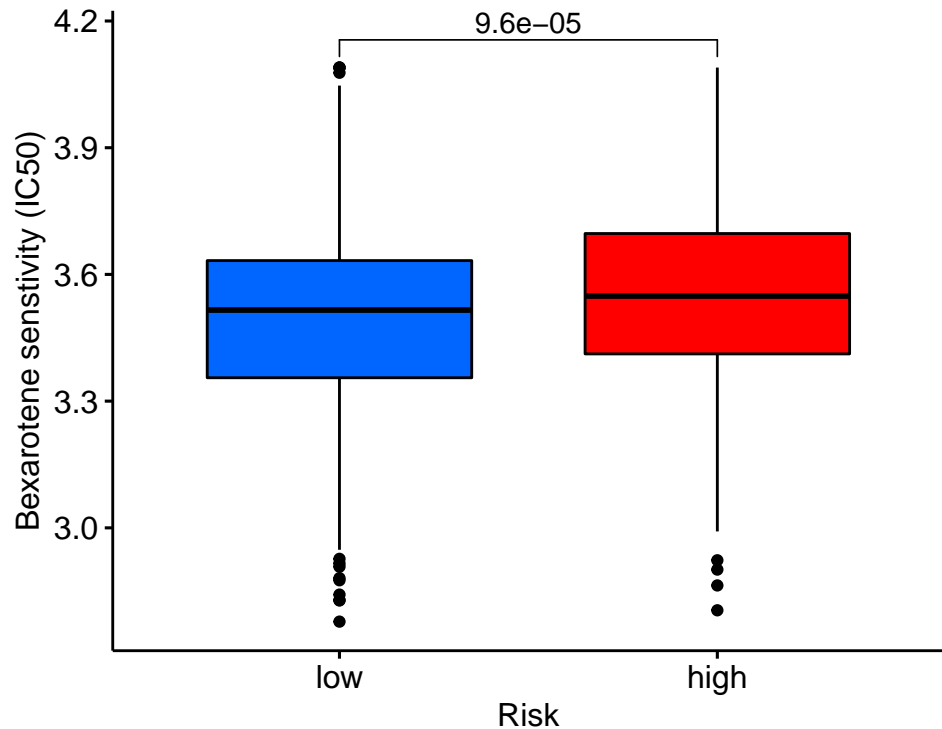

Supplement: Supplementary file 2 [file DataSheet_2.zip › durgSenstivity.Bexarotene.pdf]

Risk 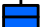 low 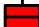 high

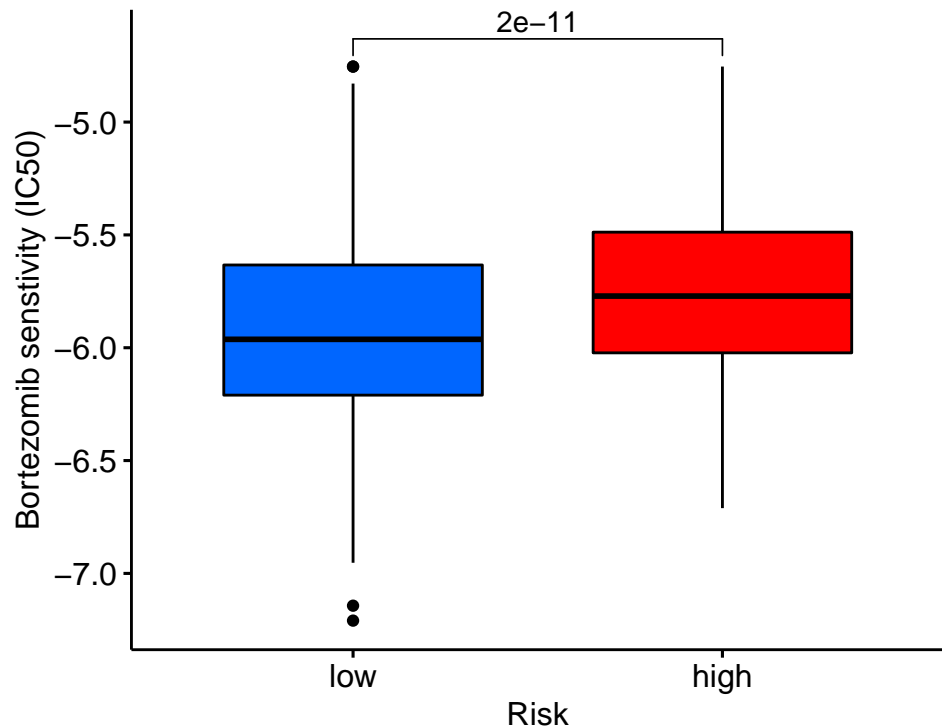

Supplement: Supplementary file 2 [file DataSheet_2.zip › durgSenstivity.Bortezomib.pdf]

Risk 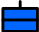 low 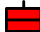 high

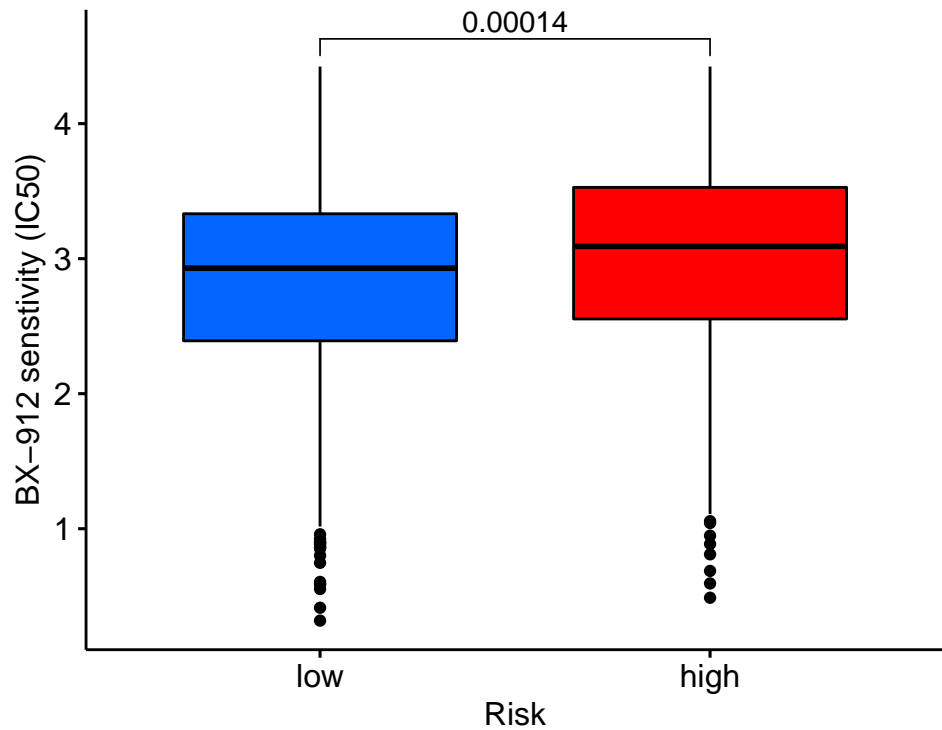

Supplement: Supplementary file 2 [file DataSheet_2.zip › durgSenstivity.BX-912.pdf]

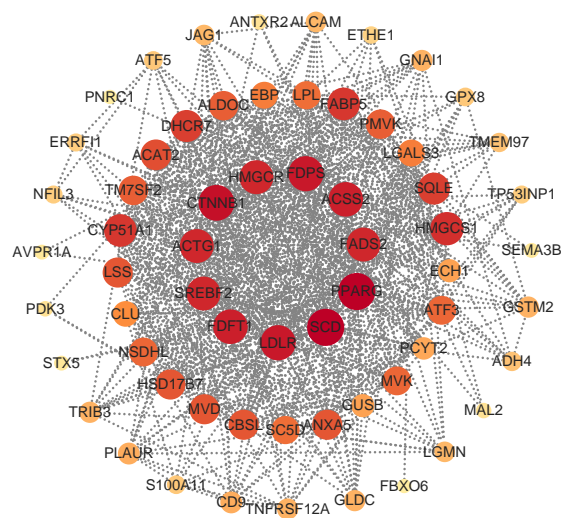

Supplement: Supplementary file 3 [file Image_1.pdf]

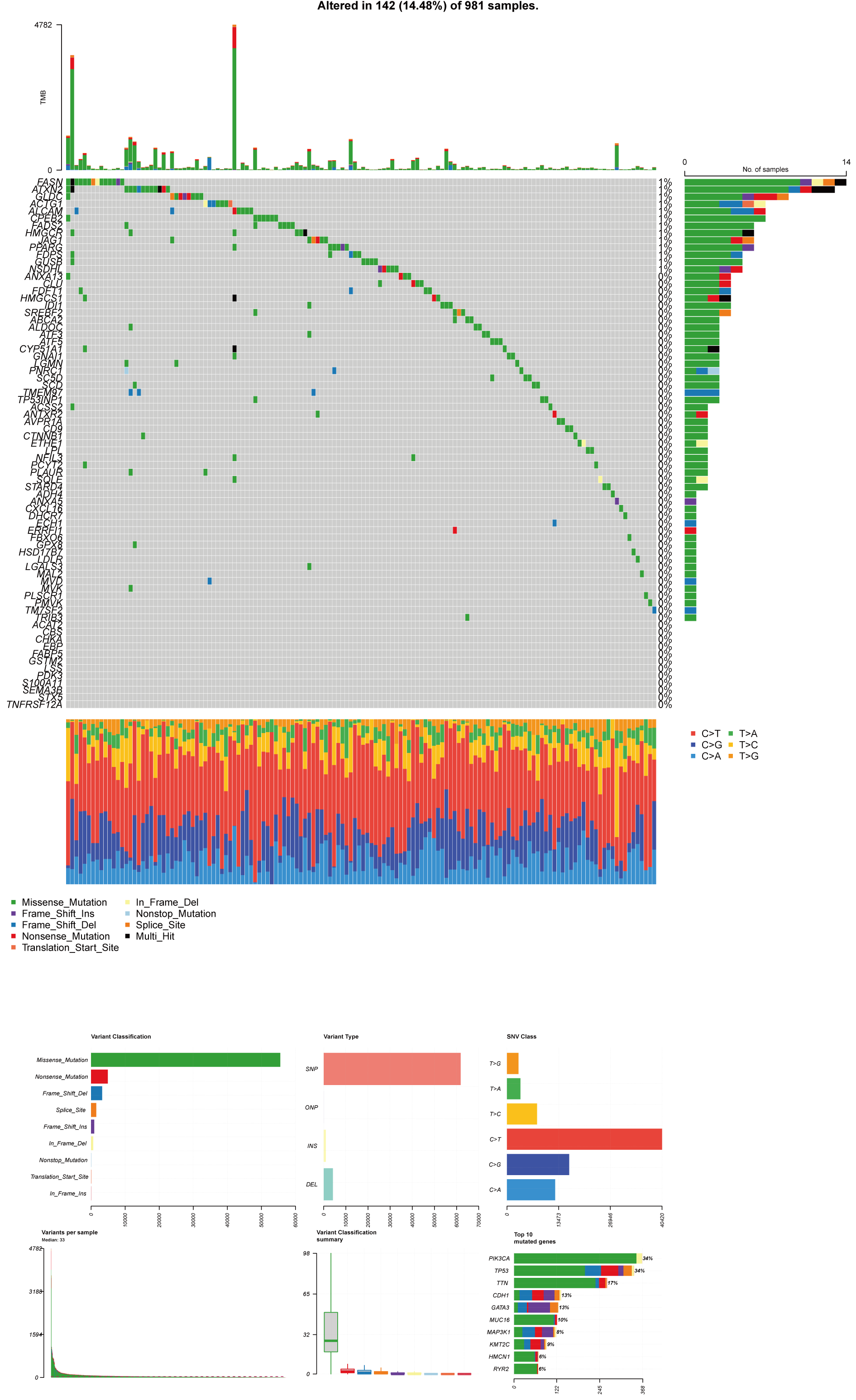

Supplement: Supplementary file 4 [file Image_2.tif]

consensus matrix k=2

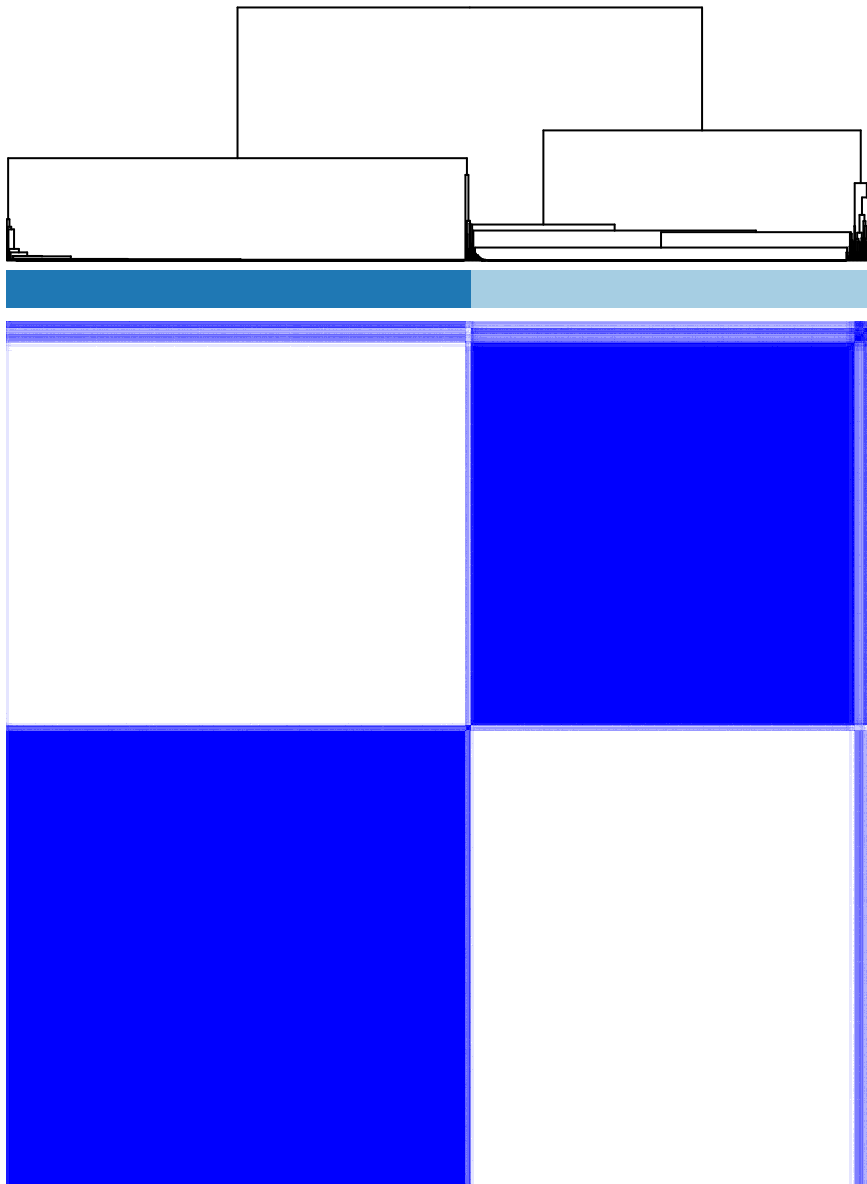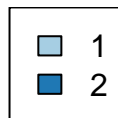

Supplement: Supplementary file 5 [file Image_3.pdf]

Expression

Type Nomal Tumor

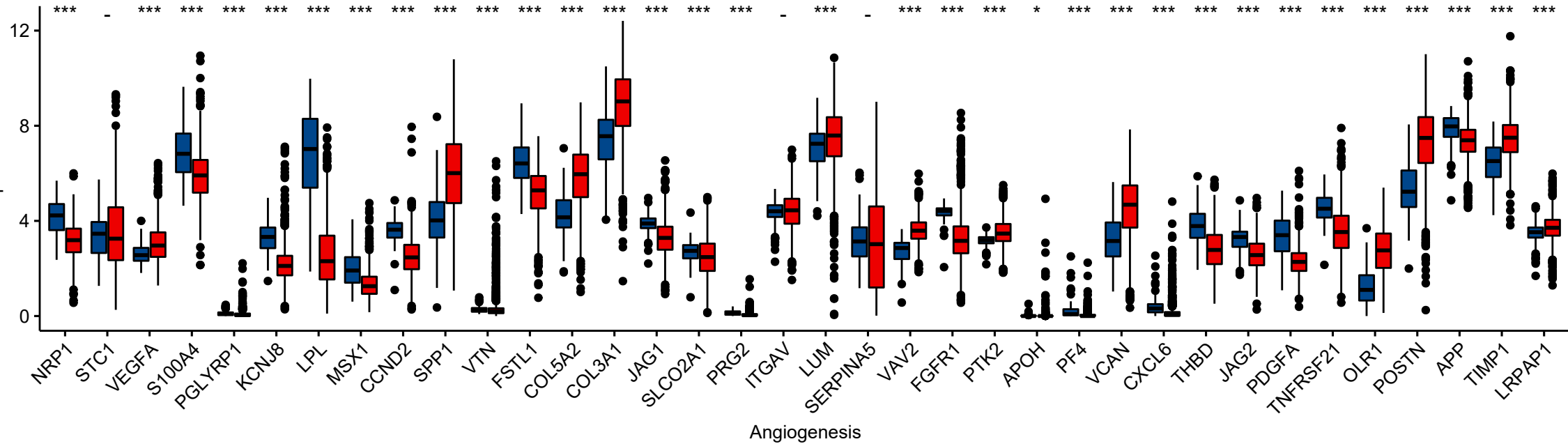

Supplement: Supplementary file 6 [file Image_4.pdf]

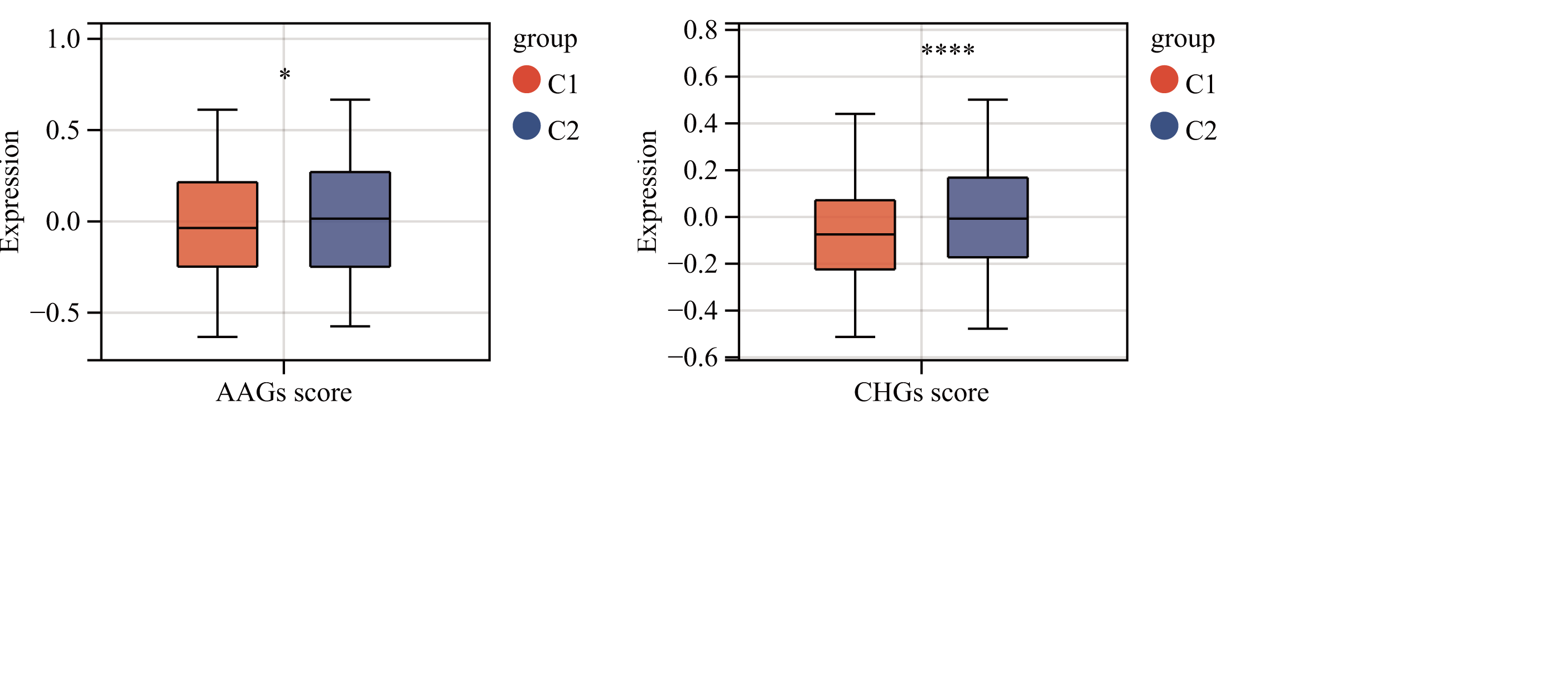

Supplement: Supplementary file 7 [file Image_5.tif]

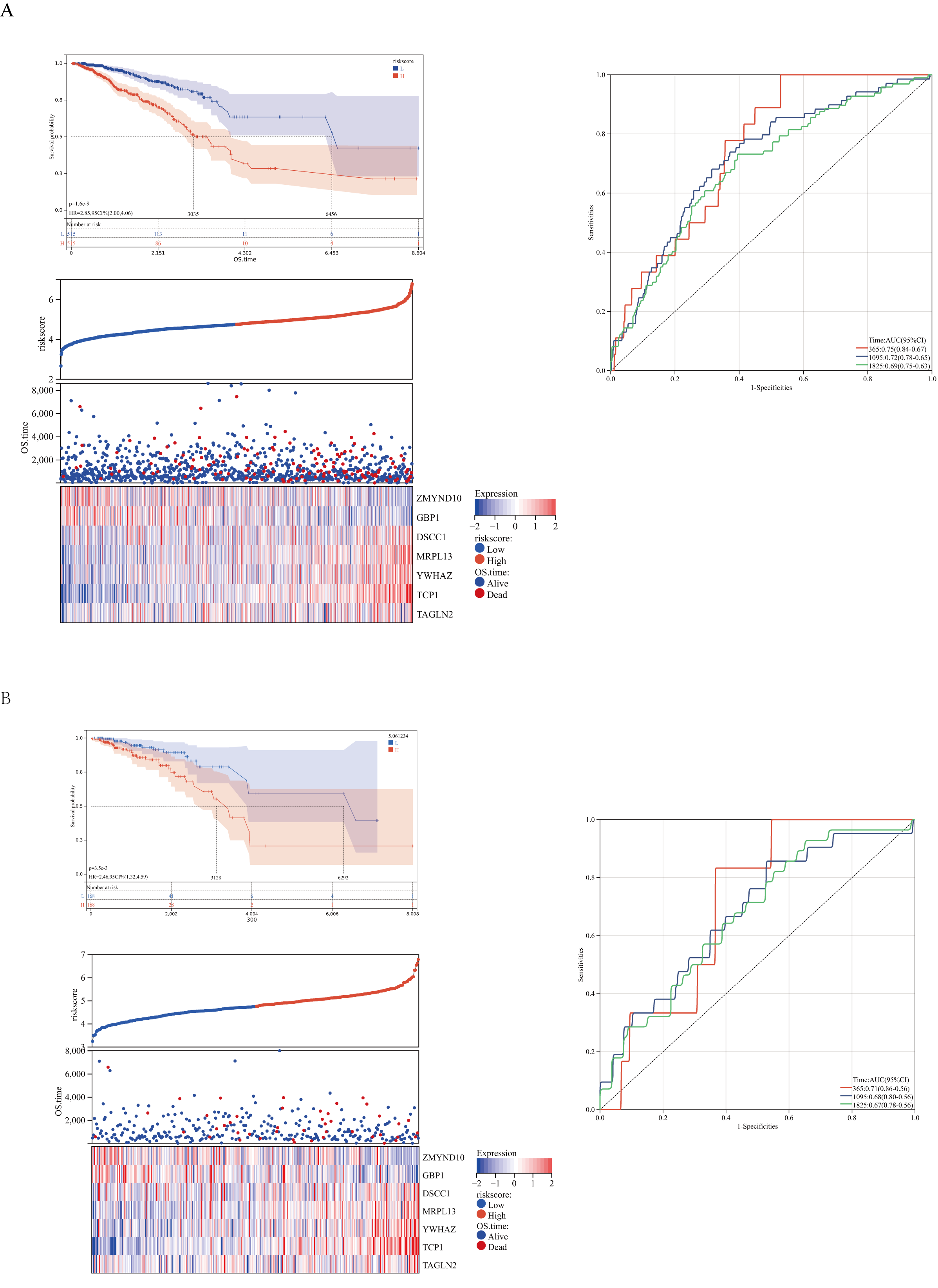

Supplement: Supplementary file 8 [file Image_6.tif]

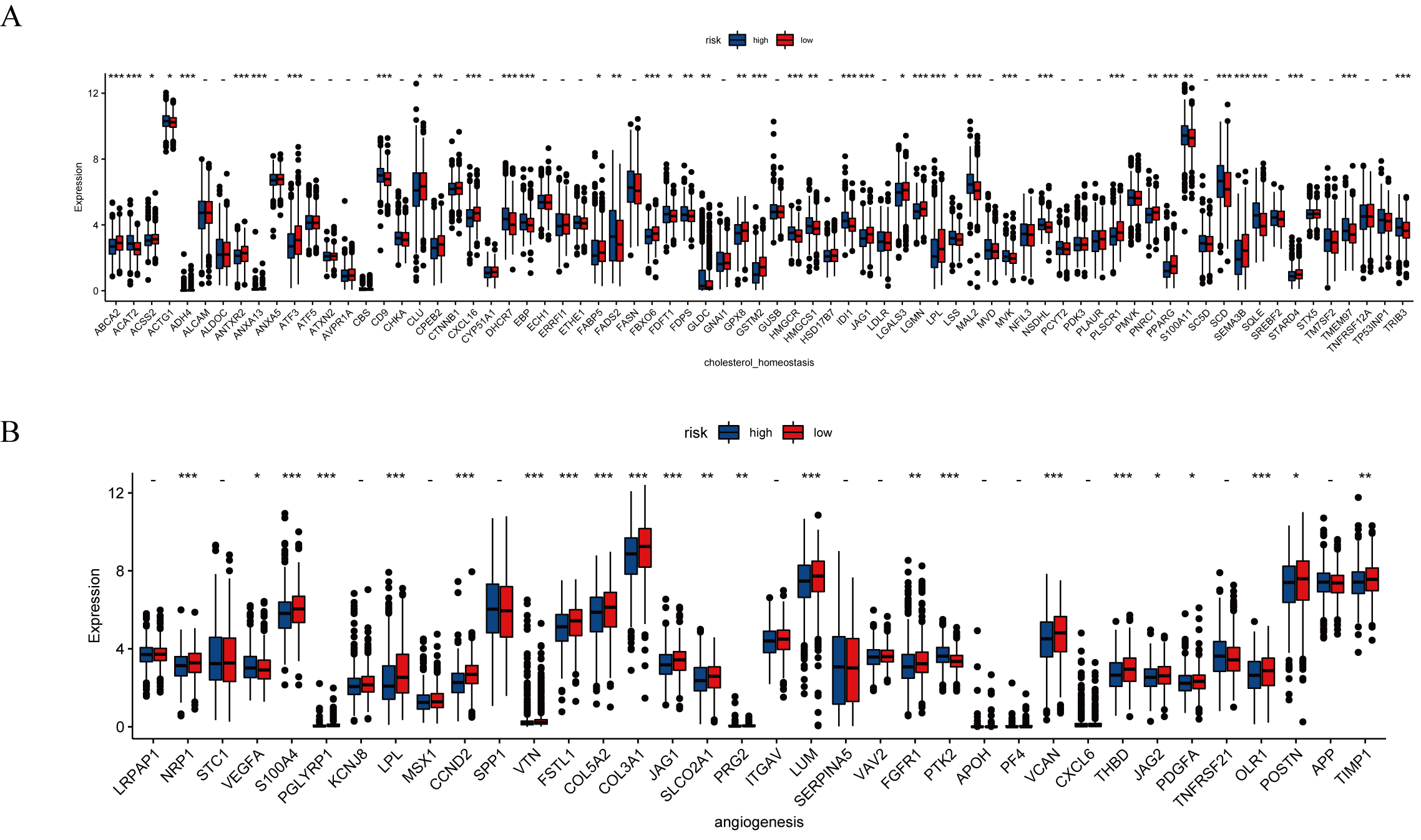

Supplement: Supplementary file 9 [file Image_7.tif]

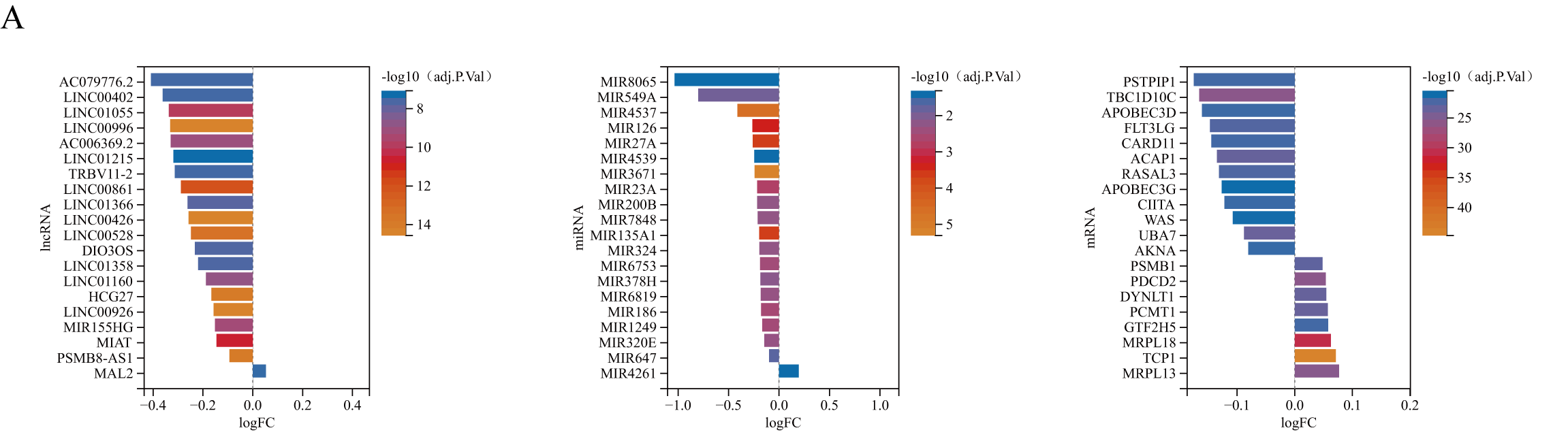

Supplement: Supplementary file 10 [file Image_8.tif]
