# Supplementary material for: Prognostic value analysis of cholesterol and cholesterol homeostasis related genes in breast cancer by Mendelian randomization and multi-omics machine learning
Source: Front Oncol. 2023 Nov 7;13:1246880. doi: 10.3389/fonc.2023.1246880 (PMC10661325; doi:10.3389/fonc.2023.1246880)
Supplement: Supplementary file 11 [file Table_1.docx]

| Cholesterol homeostasis genes | Angiogenesis genes |
| --- | --- |
| ABCA2 |  |
| ACAT2 | APOH |
| ACSS2 | APP |
| ACTG1 | CCND2 |
| ADH4 | COL3A1 |
| ALCAM | COL5A2 |
| ALDOC | CXCL6 |
| ANTXR2 | FGFR1 |
| ANXA13 | FSTL1 |
| ANXA5 | ITGAV |
| ATF3 | JAG1 |
| ATF5 | JAG2 |
| ATXN2 | KCNJ8 |
| AVPR1A | LPL |
| CBS | LRPAP1 |
| CD9 | LUM |
| CHKA | MSX1 |
| CLU | NRP1 |
| CPEB2 | OLR1 |
| CTNNB1 | PDGFA |
| CXCL16 | PF4 |
| CYP51A1 | PGLYRP1 |
| DHCR7 | POSTN |
| EBP | PRG2 |
| ECH1 | PTK2 |
| ERRFI1 | S100A4 |
| ETHE1 | SERPINA5 |
| FABP5 | SLCO2A1 |
| FADS2 | SPP1 |
| FASN | STC1 |
| FBXO6 | THBD |
| FDFT1 | TIMP1 |
| FDPS | TNFRSF21 |
| GLDC | VAV2 |
| GNAI1 | VCAN |
| GPX8 | VEGFA |
| GSTM2 | VTN |
| GUSB |  |
| HMGCR |  |
| HMGCS1 |  |
| HSD17B7 |  |
| IDI1 |  |
| JAG1 |  |
| LDLR |  |
| LGALS3 |  |
| LGMN |  |
| LPL |  |
| LSS |  |
| MAL2 |  |
| MVD |  |
| MVK |  |
| NFIL3 |  |
| NIBAN1 |  |
| NSDHL |  |
| PCYT2 |  |
| PDK3 |  |
| PLAUR |  |
| PLSCR1 |  |
| PMVK |  |
| PNRC1 |  |
| PPARG |  |
| S100A11 |  |
| SC5D |  |
| SCD |  |
| SEMA3B |  |
| SQLE |  |
| SREBF2 |  |
| STARD4 |  |
| STX5 |  |
| TM7SF2 |  |
| TMEM97 |  |
| TNFRSF12A | |
| TP53INP1 |  |
| TRIB3 |  |
