# Supplementary material for: Prognostic value analysis of cholesterol and cholesterol homeostasis related genes in breast cancer by Mendelian randomization and multi-omics machine learning
Source: Front Oncol. 2023 Nov 7;13:1246880. doi: 10.3389/fonc.2023.1246880 (PMC10661325; doi:10.3389/fonc.2023.1246880)
Supplement: Supplementary file 12 [file Table_2.docx]

| GENE | direction | | prodSize | | Sequence | |
| --- | --- | --- | --- | --- | --- | --- |
| human ZMYND10 | | FORWARD | | 132 | | AGAAGCTGAACATGCAAGCC |
| human ZMYND10 | | REVERSE | | 132 | | TCTGCTTCCACATCTCCACTG |
| human GBP1 | | FORWARD | | 150 | | AGTGGAACGTGTGAAAGCTG |
| human GBP1 | | REVERSE | | 150 | | ACCCTGTCGTTCTCCATCTTC |
| human DSCC1 | | FORWARD | | 140 | | ACCATTGGAGCCAGAGGAAATG |
| human DSCC1 | | REVERSE | | 140 | | CCGCATTCTGAAGTAGCATTCG |
| human MRPL13 | | FORWARD | | 80 | | ACTCTTCGCATACTGGCTACC |
| human MRPL13 | | REVERSE | | 80 | | AATTGCCACTGGATCCCTCAG |
| human YWHAZ | | FORWARD | | 91 | | GGTGCACAAGATTACCTTCCTG |
| human YWHAZ | | REVERSE | | 91 | | TCATGCGGCCTTTTTCCAAG |
| human TCP1 | | FORWARD | | 106 | | TTGTGGAGGCTGGTGCTATG |
| human TCP1 | | REVERSE | | 106 | | TCCAAATTGGCCAGGGTTGA |
| human TAGLN2 | | FORWARD | | 121 | | ATGGGCTCTTCTCTGGGGAT |
| human TAGLN2 | | REVERSE | | 121 | | TTGGTGCCCATCTGTAACCC |
