# Supplementary material for: Prognostic value analysis of cholesterol and cholesterol homeostasis related genes in breast cancer by Mendelian randomization and multi-omics machine learning
Source: Front Oncol. 2023 Nov 7;13:1246880. doi: 10.3389/fonc.2023.1246880 (PMC10661325; doi:10.3389/fonc.2023.1246880)
Supplement: Supplementary file 14 [file Table_4.docx]

Table S4. Analysis of the difference of clinical features between C1 and C2 groups

| **Characteristics** | **C2(N=455)** | **C1(N=310)** | **Total(N=765)** | **pvalue** | **FDR** |
| --- | --- | --- | --- | --- | --- |
| **group** |  |  |  | 1.70E-167 | 1.60E-166 |
| C1 | 0(0.0e+0%) | 310(40.52%) | 310(40.52%) |  |  |
| C2 | 455(59.48%) | 0(0.0e+0%) | 455(59.48%) |  |  |
| age |  |  |  | 0.49 | 0.49 |
| <50 | 119(15.56%) | 89(11.63%) | 208(27.19%) |  |  |
| >50 | 336(43.92%) | 221(28.89%) | 557(72.81%) |  |  |
| **ER_Status** |  |  |  | 9.00E-23 | 7.20E-22 |
| Indeterminate | 2(0.27%) | 0(0.0e+0%) | 2(0.27%) |  |  |
| Negative | 155(21.15%) | 11(1.50%) | 166(22.65%) |  |  |
| Positive | 283(38.61%) | 282(38.47%) | 565(77.08%) |  |  |
| **HER2_Status** |  |  |  | 0.03 | 0.14 |
| Equivocal | 6(0.80%) | 4(0.53%) | 10(1.33%) |  |  |
| Negative | 362(48.14%) | 268(35.64%) | 630(83.78%) |  |  |
| Positive | 79(10.51%) | 33(4.39%) | 112(14.89%) |  |  |
| **PR_Status** |  |  |  | 3.90E-22 | 2.70E-21 |
| Indeterminate | 3(0.41%) | 1(0.14%) | 4(0.55%) |  |  |
| Negative | 204(27.87%) | 34(4.64%) | 238(32.51%) |  |  |
| Positive | 232(31.69%) | 258(35.25%) | 490(66.94%) |  |  |
| **Tumor** |  |  |  | 6.40E-03 | 0.04 |
| T1 | 100(13.07%) | 101(13.20%) | 201(26.27%) |  |  |
| T2 | 284(37.12%) | 176(23.01%) | 460(60.13%) |  |  |
| T3 | 54(7.06%) | 24(3.14%) | 78(10.20%) |  |  |
| T4 | 17(2.22%) | 9(1.18%) | 26(3.40%) |  |  |
| **Node** |  |  |  | 0.14 | 0.28 |
| N0 | 229(29.93%) | 142(18.56%) | 371(48.50%) |  |  |
| N1 | 138(18.04%) | 118(15.42%) | 256(33.46%) |  |  |
| N2 | 64(8.37%) | 34(4.44%) | 98(12.81%) |  |  |
| N3 | 24(3.14%) | 16(2.09%) | 40(5.23%) |  |  |
| **Metastasis** |  |  |  | 0.08 | 0.25 |
| M0 | 443(57.98%) | 307(40.18%) | 750(98.17%) |  |  |
| M1 | 12(1.57%) | 2(0.26%) | 14(1.83%) |  |  |
| **pathologic_stage** |  |  |  | 0.03 | 0.14 |
| Stage I | 67(8.76%) | 65(8.50%) | 132(17.25%) |  |  |
| Stage II | 267(34.90%) | 179(23.40%) | 446(58.30%) |  |  |
| Stage III | 109(14.25%) | 64(8.37%) | 173(22.61%) |  |  |
| Stage IV | 12(1.57%) | 2(0.26%) | 14(1.83%) |  |  |
