# Supplementary material for: Prognostic value analysis of cholesterol and cholesterol homeostasis related genes in breast cancer by Mendelian randomization and multi-omics machine learning
Source: Front Oncol. 2023 Nov 7;13:1246880. doi: 10.3389/fonc.2023.1246880 (PMC10661325; doi:10.3389/fonc.2023.1246880)
Supplement: Supplementary file 2 [file durgSenstivity.Zibotentan.pdf › durgSenstivity.Zibotentan.pdf]

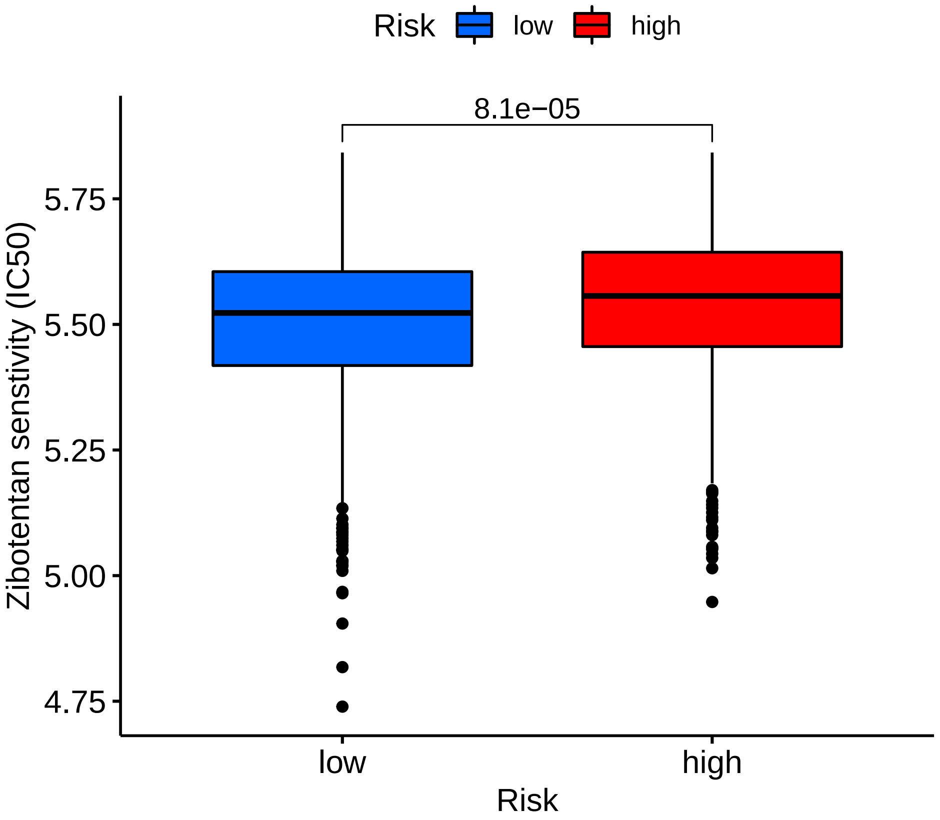
Risk
low
high
8.1e−05
Zibotentan senstivity (IC50)
5.75
5.50
5.25
5.00
4.75
low
high
Risk
